# Bypassing Schottky constraints via defect-mediated hydrogen transfer in hydrodechlorination

**DOI:** 10.1038/s41467-026-74751-0

**Published:** 2026-06-22

**Authors:** Shunjie Zhu, Haoran Chen, Zhongjie Cui, Shiwei Wang, Li-Zhi Huang

**Affiliations:** 1 School of Civil Engineering, Wuhan University, Wuhan, PR China; 2 School of Environmental Science and Engineering, Huazhong University of Science and Technology, Wuhan, PR China; 3 Key Laboratory of Resource Biology and Biotechnology in Western China, Ministry of Education, Provincial Key Laboratory of Biotechnology, College of Life Sciences, Northwest University, Xi’an, PR China

**Keywords:** Environmental chemistry, Chemical engineering, Heterogeneous catalysis

## Abstract

Hydrogen-mediated transformations at heterointerfaces are often limited by a fundamental kinetic mismatch: the need for strong interfacial polarization to stabilize active hydrogen (H*) often creates high Schottky barriers that impede the necessary electron supply. Here, using a defect-engineered MoS_2_/Fe(OH)_2_ heterojunction for chloroform (CF) hydrodechlorination, we resolve this trade-off by engineering a defect-band hybridization mechanism. We observe that while increasing sulfur-vacancy density in MoS_2_ upshifts its conduction-band minimum—nominally raising the barrier for electron injection—it paradoxically enhances interfacial electron transport. Electronic structure analysis reveals that interfacial electric-field coupling drives strong hybridization between vacancy-derived states and the MoS_2_ conduction band. This forms quasi-continuous, defect-assisted tunneling channels that allow electrons to bypass the energetic barrier. The resulting electronic reconstruction stabilizes interfacial H* in a reactive yet mobile state, shifting the reaction regime from an Fe(II)-dominated single-electron transfer to a precise H*-mediated hydrogen atom transfer pathway. This mechanism suppresses hydrogen evolution and enables highly selective deep hydrodechlorination, providing a generalizable blueprint for manipulating electron–proton coupling at mismatched heterointerfaces.

## Abstract (web summary)

This study shows that sulfur vacancies in MoS_2_/Fe(OH)_2_ open defect-assisted electron pathways that bypass Schottky barriers, mobilize active hydrogen and drive selective chloroform hydrodechlorination.

## Introduction

Hydrogen-mediated catalytic reactions, including hydrogenation and hydrodehalogenation^1,2^, critically depend on the formation, stabilization, and delivery of active hydrogen (H*)^3^. In heterogeneous catalysts, these processes are intrinsically coupled to proton-coupled electron transfer (PCET)^4^, which requires coordinated electron redistribution and proton activation at interfaces. As a result, hydrogen spillover has been widely recognized as a key route to spatially separate hydrogen generation from substrate conversion by transporting adsorbed hydrogen species from hydrogen-producing sites to spatially separated reaction sites^5,6^. By extending the availability of H* beyond the original generation domains, spillover can deliver active hydrogen to locations with more favorable substrate adsorption and thereby enable selectivity that is difficult to achieve on a single-phase surface.

However, cross-interface hydrogen transport is not intrinsically efficient and is highly sensitive to interfacial coupling strength and energy-level alignment. Unfavorable level alignment (e.g., large Fermi-level/band-edge offsets) and pronounced differences in hydrogen adsorption properties between the two phases can increase the kinetic resistance for H* transfer^7^. Additionally, in practical catalysts, H* spillover typically proceeds through a continuous, multistep transfer process that traverses multiple interfaces before reaching the reactive sites^8–10^. Disruption of any interfacial segment by structural reconstruction or loss of intimate contact can increase the effective migration barrier and render hydrogen delivery intermittent^11^.

More importantly, much of the current literature treats hydrogen spillover in a yes/no manner. The actual migration pathways within heterojunction interfaces, as well as their response to interfacial charge accumulation and band bending, remain poorly resolved. Notably, under such misaligned and transport-limited conditions, electrons tend to electrons tend to localize and accumulate on one side of the junction, often near semiconductor defect-derived states, leading to higher interfacial H* coverage but stronger H* binding^12,13^. This hydrogen trapping scenario hampers H* desorption, surface diffusion, and interfacial spillover, such that an apparently higher H* population does not translate into a higher flux of turnover-ready active hydrogen.

These limitations motivate a central question in hydrogen-involving catalysis: how can one engineer a heterointerface to retain a high population of active hydrogen while maintaining a high turnover flux, given that increasing interfacial charge separation often raises the effective barrier for carrier delivery^14–17^ and promotes hydrogen trapping at the acceptor side?

To address this challenge, we employ chloroform (CF) hydrodechlorination as a model reaction and construct a defect-engineered MoS_2_/Fe(OH)_2_ heterojunction, in which Fe(OH)_2_ serves as the hydrogen-generating phase while MoS_2_ functions as a hydrogen-accepting^18–20^ and reaction-directing surface^21,22^. We show that increasing the S-vacancy concentration in MoS_2_ upshifts its conduction-band minimum and nominally increases the interfacial Schottky barrier (SBH), yet paradoxically enhances interfacial electron delivery and the effective flux of active hydrogen across the heterointerface. Through a combination of electronic-structure analysis, hydrogen-species probing, and kinetic mechanistic experiments, we demonstrate that strong coupling between vacancy-derived states and the MoS_2_ conduction band establishes a defect-assisted electron-transport pathway that stabilizes and mobilizes interfacial H*, thereby shifting the dominant reaction pathway from Fe(II)-driven single-electron transfer (SET) toward H*-mediated hydrogen atom transfer (HAT) and enabling highly selective deep hydrodechlorination.

## Results and discussion

### Structural and electronic reconstruction

**Fig. 1:**
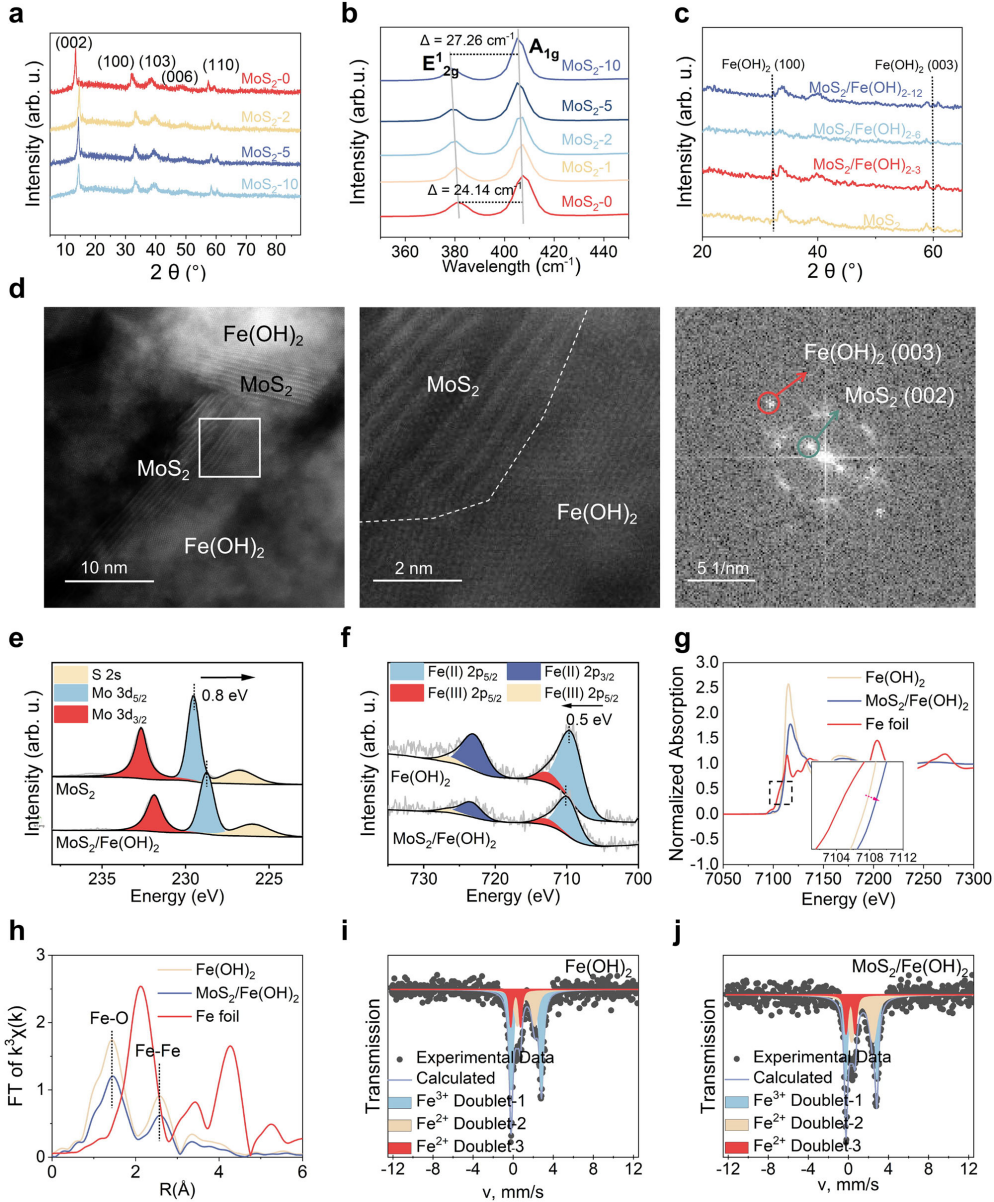
Structural and spectroscopic characterization of the MoS2/Fe(OH)2 heterojunction. **a** XRD patterns of MoS_2_ treated with different H_2_O_2_ concentrations. **b** Raman spectra of MoS_2_ treated with different H_2_O_2_ concentrations. **c** XRD patterns of the MoS_2_/Fe(OH)_2_ heterojunction. **d** HRTEM image showing the intimate interfacial contact between MoS_2_ (002) and Fe(OH)_2_ (003). **e** Mo 3 d XPS spectra. **f** Fe 2p XPS spectra. **g** Fe K-edge XANES spectra. **h** Fourier-transformed k^3^-weighted EXAFS spectra. **i** ^57^Fe Mössbauer spectrum of pristine Fe(OH)_2_. **j** ^57^Fe Mössbauer spectrum of MoS_2_/Fe(OH)_2_. Source data are provided in Source Data 1.

We prepared the MoS_2_/Fe(OH)_2_ heterojunction and characterized its interfacial coupling characteristics as well as electronic structure evolution starting from the microstructure. Constructing an active interface with efficient electron transport channels is a prerequisite for achieving superior catalytic performance, which primarily relies on the heterojunction construction and the precise modulation of surface defects on the substrate. We employed H_2_O_2_ as a specific surface etchant to induce sulfur vacancies on the MoS_2_ basal plane and achieved controllable modulation of the defect density by finely regulating the H_2_O_2_ concentration. We show that with increasing H_2_O_2_ treatment concentration, the diffraction peak intensities of the MoS_2_ (002) and (006) planes exhibited only slight attenuation (Fig. 1a), and the peak positions remained unchanged. This result indicated that this treatment strategy primarily acts on the crystal basal plane without disrupting the long-range ordered structure of the MoS_2_ bulk phase, thereby preserving the crystallinity of bulk MoS_2_. Raman spectra (Fig. 1b) showed subtle changes in the local coordination environment. As the treatment concentration increased, the frequency difference between the E^1^_2g_ (~383 cm^−1^) and A_1g_ (~408 cm^−1^) modes showed a slight increasing trend, rising from 24.1 to 27.6 cm^−1^. Distinct from the frequency decrease typically caused by interlayer exfoliation^23^, this minor increase was attributed to the local lattice stress and distortion induced by the introduction of S-vacancies. XPS characterization supported the fine etching mechanism of H_2_O_2_ on the MoS_2_ surface chemistry. Analysis by XPS was conducted to investigate the surface structural changes of MoS_2_ during the etching process. As shown in Table S1, with increasing H_2_O_2_ concentration, the S/Mo ratio gradually decreased, and the concentration of S-vacancies increased from 0.00 to 9.05%. XPS spectra (Fig. S2) revealed that in the initial state, MoS_2_ contained a certain proportion of oxidized S^6+^, which gradually decreased and eventually disappeared as the H_2_O_2_ concentration increased. This surface activation induced by defect engineering, through the in-situ exposure of abundant coordinatively unsaturated Mo sites, effectively modulated the surface electronic state, thereby significantly improving the intrinsic hydrophilicity of the material. Contact angle measurements (Fig. S3) showed that the contact angle significantly decreased with increasing H_2_O_2_ concentration. Additionally, FTIR spectra (Fig. S4) displayed enhanced O-H and H-O-H characteristic vibration peaks at 3437 cm^−1^ and 1640 cm^−1^, respectively.

Based on the surface-activated MoS_2_ substrate, the MoS_2_/Fe(OH)_2_ heterojunction was constructed via a co-precipitation synthesis method. Due to the good lattice matching between MoS_2_ (space group P6_3_/mmc, *a* = 3.15 Å) and Fe(OH)_2_ (space group P-3m1, *a* = 3.26 Å), the low lattice mismatch of only 3.3% effectively alleviated interfacial lattice stress, enabling Fe(OH)_2_ nanosheets to be firmly anchored onto the MoS_2_ basal plane, forming a stable heterogeneous interface. XRD patterns (Fig. 1c) showed that after introducing Fe(OH)_2_, distinct characteristic peaks appeared at 2θ ≈ 32.3° and 60.2°, assigned to the Fe(OH)_2_ (100) and (003) planes^24^. High-resolution transmission electron microscopy (HRTEM) resolves lattice fringes corresponding to MoS_2_ (002) and Fe(OH)_2_ (003) in close contact at the interface (Fig. 1d), indicating intimate interfacial integration. This observation is further supported by EDS mapping (Fig. S13), which shows Fe and O signals distributed in regions adjacent to and contacting the MoS_2_ nanosheets (Mo and S), consistent with the formation of a MoS_2_/Fe(OH)_2_ heterojunction. To deeply reveal the electronic driving mechanism behind this interfacial coupling, we conducted systematic analyses combining DFT calculations. Work function calculations (Fig. S5) indicated that the large work function difference of 3.43 eV between Fe(OH)_2_ and MoS_2_ generates a strong thermodynamic driving force, compelling electrons to spontaneously flow across the interface from Fe(OH)_2_ to MoS_2_ until the Fermi levels reach equilibrium, thereby establishing a robust built-in electric field at the interface^25,26^.

High-resolution spectroscopic characterization strongly corroborated this interfacial electron transfer mechanism. XPS analysis (Fig. 1e, f) showed the energy level evolution before and after heterojunction formation. The binding energy of Fe^2+^ 2p_3/2_ in Fe(OH)_2_ shifted positively from 710.3 to 710.8 eV, while the Mo^4+^ 3d_5/2_ peak in MoS_2_ exhibited the opposite trend, shifting to lower energy from 229.8 to 229.0 eV. This significant and opposing binding energy shift strongly suggested the process of electron injection from Fe(OH)_2_ across the interface into MoS_2_ driven by the built-in electric field^27,28^. Fe K-edge XANES spectra (Fig. 1g) further revealed that relative to pristine Fe(OH)_2_, the absorption edge of the heterojunction shifted noticeably toward higher energy, indicating an increased average valence state of Fe^2+^ species^29^, which was highly consistent with the electron loss of Fe observed in XPS. Furthermore, EXAFS spectra (Fig. 1h) showed a significant attenuation in the intensity of Fe-O and Fe-Fe coordination peaks in the heterojunction. To elucidate the structural origin of this attenuation, we performed quantitative EXAFS fitting for pristine Fe(OH)_2_ and MoS_2_/Fe(OH)_2_ (Fig. S22), with the fitting parameters summarized in Table S2. The fitting results indicate a reduced effective Fe-O coordination number (CN from 6.72 to 5.26), suggesting enhanced local structural under-coordination upon coupling with MoS_2_. This phenomenon implied the inhibitory effect of the MoS_2_ substrate on the independent bulk growth of Fe(OH)_2_, prompting Fe species to participate in the construction of the heterogeneous interface in a more highly dispersed form.

To further resolve the evolution of the active center electronic configuration induced by charge redistribution at the atomic scale, we performed high-sensitivity Mössbauer spectroscopy characterization (Fig. 1i, j). Peak fitting results indicated that the Fe species are primarily composed of octahedrally coordinated Fe^3+^ (Doublet 1, area ratio 14–15.9%) and two groups of high-spin Fe^2+^ with varying degrees of distortion. Among them, Fe^2+^ Doublet 2 exhibited a large quadrupole splitting (QS) value, corresponding to strong Jahn-Teller distortion. In the pristine Fe(OH)_2_ lattice, asymmetric electron occupation of degenerate orbitals induced strong lattice distortion to eliminate degeneracy^30,31^. However, after coupling with MoS_2_, the formation of the interfacial built-in electric field extracted electrons from the Fe(OH)_2_ side. This directional electron transfer effectively alleviated the electronic asymmetry of the Fe^2+^ 3*d* orbitals, thereby triggering a deep electronic reconstruction. This reconstruction was specifically manifested as a significant reduction in QS values (Doublet 2 from 3.09 to 3.03 mm s^−1^, Doublet 3 from 2.15 to 1.74 mm s^−1^), indicating that lattice distortion was effectively inhibited and Fe^2+^ transformed into a more catalytically active, high-symmetry octahedral configuration^32^. Concurrently, the narrowing of the line width for Doublet 3 (Γ: 0.87 → 0.71 mm s^−1^) and the decrease in isomer shift (IS: 1.39 → 1.28 mm s^−1^) collectively revealed a trend toward homogenization of electron density around the Fe nucleus^33^. This low-distortion, highly ordered Fe^2+^ active center, optimized via interfacial electronic coupling, possessed more favorable orbital symmetry, laying the structural foundation for the subsequent efficient generation of H*.

### Barrier-bypass mechanism via orbital hybridization

**Fig. 2:**
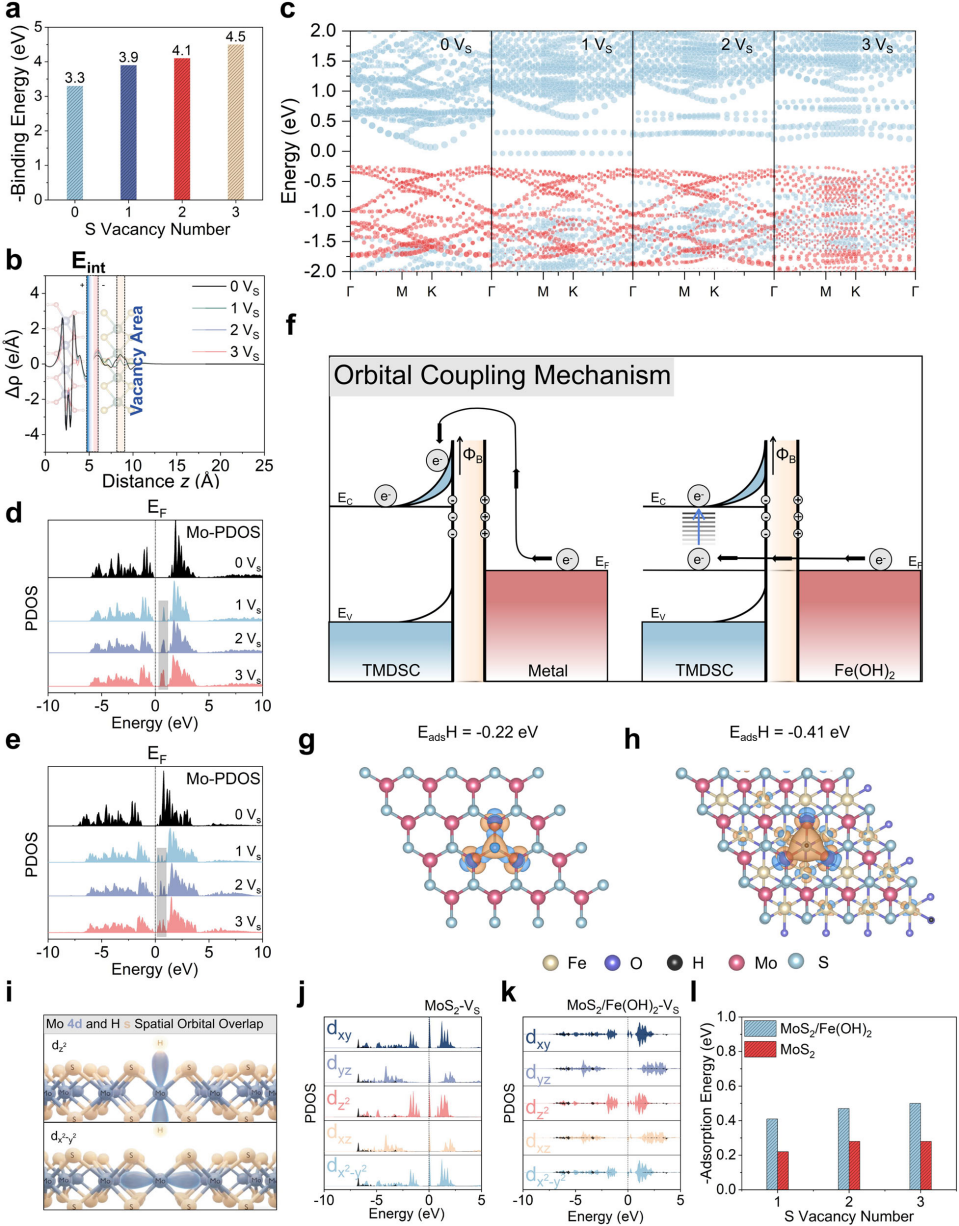
Vacancy-regulated electronic structure and hydrogen adsorption on the MoS2/Fe(OH)2 heterojunction. **a** Calculated binding energies of MoS_2_/Fe(OH)_2_ heterojunctions with increasing S-vacancy concentrations of 0%, 3.1%, 6.3% and 9.4%. **b** Planar-averaged charge density difference along the z-axis of the heterojunction. **c** Evolution of the MoS_2_ band structure in the heterojunction as a function of S-vacancy concentration. **d**, **e** Projected density of states (PDOS) of isolated defective MoS_2_ and the MoS_2_/Fe(OH)_2_ heterojunction, respectively; the gray shaded regions indicate defect states. **f** Schematic illustration of the electron-transport mechanism. **g**, **h** Charge density difference maps of H* adsorbed at the S-vacancy site in isolated MoS_2_ and the MoS_2_/Fe(OH)_2_ heterojunction, respectively, where yellow and blue regions indicate electron accumulation and depletion, respectively. **i** Schematic representation of the orbital interaction between the out-of-plane Mo ${d}_{{z}^{2}}$ orbital and the H 1*s* orbital. **j**, **k** PDOS analysis of the hybridization between Mo d orbitals and H 1*s* orbitals in MoS_2_-V_S_ and MoS_2_/Fe(OH)_2_-V_S_, respectively. **l** Calculated H adsorption energies on MoS_2_ and MoS_2_/Fe(OH)_2_ surfaces with different S-vacancy concentrations. In the crystal models, yellow, purple, black, red and blue represent Fe, O, H, Mo and S atoms, respectively. Source data are provided in Source Data 1.

We constructed four models with varying S-vacancy concentrations (0%, 3.1%, 6.3%, and 9.4%) and performed density functional theory (DFT) calculations to quantify how S-vacancy concentration modulates charge redistribution and band alignment at the MoS_2_/Fe(OH)_2_ heterojunction interface. Increasing S-vacancy concentration strengthened interfacial binding (Fig. 2a) and increased interlayer electron transfer flux, as evidenced by the significant decrease in heterojunction binding energy and the monotonic rise in electron transfer flux from 4.15 × 10^−3^ e/Å^2^ to 7.28 × 10^−3^ e/Å^2^ with increasing vacancy concentration (Fig. S6). The planar-averaged charge density difference along the z-axis confirmed that with increasing S-vacancy concentration, charge depletion on the Fe(OH)_2_ side became more pronounced, while charge accumulation on the MoS_2_ side increased (Fig. 2b), indicating that carrier directional migration intensifies with the proliferation of S-vacancies.

This increase in electron injection was accompanied by a notable energetic trade-off: band structure analysis showed that as the S-vacancy concentration rises, the intensified electron accumulation on the MoS_2_ side induced an upshift in the intrinsic conduction band minimum (CBM) of MoS_2_ (Fig. 2c), leading to an increase in the interfacial SBH^34^. In the classical Schottky junction model, a rise in SBH typically implies hindered carrier transport^14,15,35^. Moreover, excessive charge accumulation at the interface often strengthens hydrogen binding and promotes hydrogen trapping, thereby hindering subsequent migration^16^.

Subsequently, we compared the projected density of states (PDOS) of isolated MoS_2_ and the MoS_2_/Fe(OH)_2_ heterojunction to reveal the essential differences in their electron transport mechanisms. For the isolated MoS_2_ system (Fig. 2d), after introducing S-vacancies, the defect levels appeared as highly localized deep-level states. Due to the lack of modulation by external fields or heterogeneous interfaces, these isolated defect states cannot undergo effective wavefunction overlap or hybridization with the CBM, resulting in electrons being strongly confined at vacancy sites and unable to participate in long-range transport. In this case, the vacancy states were deep and localized, with limited overlap with the CBM, consistent with trap-like behavior and poor electronic connectivity.

In contrast, the MoS_2_/Fe(OH)_2_ heterojunction (Fig. 2e) exhibited distinct electronic structure characteristics. First, significant defect levels emerged near the Fermi level. Unlike the localized deep traps observed in the isolated system, these induced states exhibited delocalized features near the conduction band edge. This transformation markedly increased the density of low-energy states, consistent with enhanced electronic accessibility by providing quasi-continuous channels for electron transport^36,37^.

Crucially, this orbital coupling effect demonstrated significant concentration dependence. At low vacancy concentration (3.1%), the defect levels only weakly coupled with the CBM; whereas at high concentrations (6.3% and 9.4%), the coupling interaction was markedly enhanced. At this stage, the defect levels underwent deep hybridization with the CBM, transforming the originally localized deep levels into continuous “delocalized electron transport channels” within the bandgap.

Strong hybridization between vacancy-derived states and the MoS_2_ conduction band led to the emergence of delocalized electronic features near the Fermi level, which was consistent with facilitated electron delivery to surface-active sites despite the presence of an elevated interfacial SBH (Fig. 2f). This deep coupling reduces the energy barrier effects caused by SBH, preventing electron trapping and enhancing electron availability for reaction turnover. It also alleviates excessive charge accumulation at the interface, facilitating H* turnover. Together, these electronic-structure trends provided a basis for the experimentally observed enhancement in interfacial H* utilization and hydrogen-transfer reactivity discussed below.

### Orbital-driven H* stabilization mechanism

To further unravel this “heterojunction-defect” synergistic coupling mechanism, we analyzed the microscopic electronic structure by combining charge density difference and PDOS analyses. The charge density difference map (Fig. S7) showed that after the formation of the MoS_2_/Fe(OH)_2_ heterojunction, charge accumulation was localized around the sulfur-vacancy site, exhibiting a distinct distribution characteristic oriented “toward the defect center”. This was consistent with vacancy-associated states acting as electron-accepting sites under the built-in field. Accordingly, the local electron density at the defect sites was augmented, further enhancing the Mo-H interaction^38^. As depicted in Fig. 2g, h, the hydrogen adsorbed at the S-vacancies in the heterojunction system exhibited an enhanced charge accumulation process, signifying a substantially intensified degree of Mo-H electron transfer.

Detailed PDOS analysis of the Mo d orbitals (Figs. S8 and S9) revealed the fundamental differences in orbital interactions between the heterojunction and the isolated MoS_2_ system. In isolated MoS_2_, the defect levels near the Fermi level were dominated by the in-plane extended $d_{{\rm{xy}}} \, {{\rm{and}}} \, d_{x^{2}{-y^{2}}}$ orbitals. Intriguingly, in the MoS_2_/Fe(OH)_2_ heterojunction, the orbital composition of the defect states underwent a fundamental reversal. The primary contribution to the defect levels shifted to the $d_{{{\rm{z}}}^{2}}$ orbital perpendicular to the basal plane, and its intensity was significantly higher than the corresponding value in isolated MoS_2_. Acting as the primary orbital bridging the conduction band, the $d_{{{\rm{z}}}^{2}}$ orbital constituted an electron transport bridge across the interface. Compared to the in-plane $d_{{\rm{xy}}} \, {{\rm{and}}} \, d_{x^{2}{-y^{2}}}$ orbitals, the $d_{{{\rm{z}}}^{2}}$ orbital possessed a unique out-of-plane orientation, pointing directly outward toward the surface-active sites (Fig. 2i). The enhanced Mo-H interaction was also intuitively illustrated by the hybridization between Mo d orbitals and H 1*s* orbitals in the PDOS (Figs. 2j, k and S10). Compared to isolated MoS_2_, the heterojunction exhibited a more robust Mo-H orbital overlap region below the Fermi level. This precise stereochemical matching maximized the overlap integral between the Mo 4*d* and H 1*s* orbitals, significantly promoting H* adsorption^20,39–41^.

Subsequent H* adsorption energy calculations (Fig. 2l) supported the effectiveness of this orbital modulation mechanism. In the isolated MoS_2_ system, as the S-vacancy concentration increases from 3.1 to 9.4%, the H* adsorption energies remained at a relatively low level of −0.22 to −0.28 eV, indicating that although defects expose metal sites, their H* capture capability remained limited due to the unfavorable orbital orientation. In contrast, in the MoS_2_/Fe(OH)_2_ heterojunction system, benefiting from the defect electron filling and the dominant contribution of the Mo *d*_z_^2^ orbital, the H* adsorption energy showed a significantly enhancing trend, decreasing sequentially to −0.41 eV (3.1%), −0.47 eV (6.3%), and −0.50 eV (9.4%) with increasing vacancy concentration. This elevation in adsorption strength indicated that the heterojunction activated the originally low-activity defect sites, endowing them with higher reactivity. This supported that the $d_{{{\rm{z}}}^{2}}$-dominated electronic states significantly strengthened the electronic coupling between the active site and the H* intermediate, providing a plausible basis for hydrogen transfer steps.

### Interfacial H* reservoir formation and spillover kinetics

**Fig. 3:**
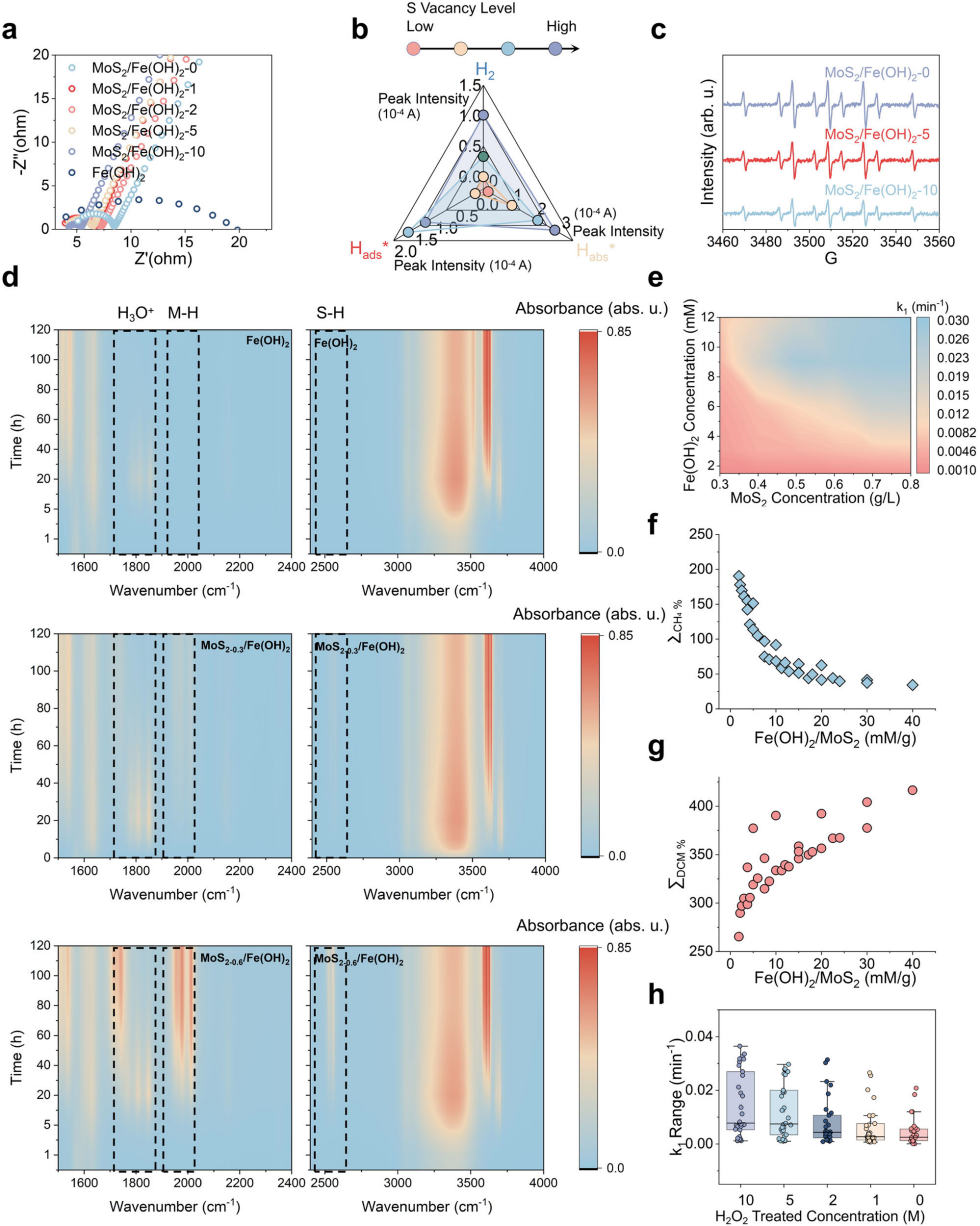
Electrochemical, spectroscopic and kinetic evidence for S-vacancy-dependent hydrogen activation. **a** Electrochemical impedance spectroscopy (EIS) Nyquist plots of MoS_2_/Fe(OH)_2_ heterojunctions prepared with different H_2_O_2_ treatment concentrations. **b** Radar chart of cyclic voltammetry (CV) peak intensities for different hydrogen species; values are plotted in units of 10^−4^ A, and the shaded area represents the CV peak-intensity profiles of different catalysts. **c** Electron paramagnetic resonance (EPR) spectra of DMPO-H*, with the x-axis shown as magnetic field, mT/G. **d** In situ infrared spectra of Fe(OH)_2_ and MoS_2_/Fe(OH)_2_ heterojunctions; the color scale represents the relative intensity of infrared peaks (abs. u.). **e** Degradation rate constant k_1_ of CF under different MoS_2_ and Fe(OH)_2_ loadings; the color scale represents k_1_ (min^−1^). **f** CH_4_ selectivity as a function of the Fe(OH)_2_/MoS_2_ ratio. **g** DCM selectivity as a function of the Fe(OH)_2_/MoS_2_ ratio. **h** Box plots of the CF degradation rate constant k_1_ grouped by H_2_O_2_ treatment concentration. For each H_2_O_2_ concentration, all experimental groups prepared under the same H_2_O_2_ treatment condition were pooled to generate the box plot. The center line indicates the median, box limits indicate the 25th and 75th percentiles, whiskers indicate 1.5 times the interquartile range, and points indicate individual measurements. Source data are provided in Source Data 1.

To connect interfacial charge dynamics with hydrogen-species evolution, we combined electrochemical impedance spectroscopy (EIS), cyclic voltammetry (CV), and electron paramagnetic resonance (EPR) to systematically investigate the impact of charge dynamics within the heterojunction on the evolution of hydrogen species. EIS (Fig. 3a) showed that as the S-vacancy concentration increased, the high-frequency semicircle radius of the MoS_2_/Fe(OH)_2_ heterojunction in the Nyquist plot exhibited a monotonic shrinking trend. This significant reduction in impedance directly indicated that the strong interfacial coupling effect induced by S-vacancies effectively weakened the interfacial contact resistance. This observation was consistent with improved electronic communication across the interface, confirming that the modified interface created a thermodynamically favorable electronic environment for hydrogen-related interfacial turnover. Building on this, CV measurements (Fig. S16) further quantified the surface hydrogen species concentration. In a substrate-free environment, with the optimization of S-vacancy concentration, the gaseous H_2_ oxidation peak representing the ineffective hydrogen evolution side reaction gradually disappeared, replaced by a significant enhancement of signals for surface-adsorbed (H_ads_*) and lattice-absorbed (H_abs_*) states^42,43^. This trend (Fig. 3b) suggests that vacancy-rich interfaces favor retention of active hydrogen as surface/lattice species rather than as molecular H_2_, consistent with the formation of an interfacial H reservoir.

Upon introducing CF (Fig. S17), the oxidation peak intensities of both H_ads_* and H_abs_* decayed dramatically or even vanished, consistent with these species being consumed during CF reduction, rather than being consumed in hydrogen evolution side reactions. This conclusion was further corroborated by DMPO trapping EPR experiments (Figs. 3c and S18). As the Fe(OH)_2_ content increased, the DMPO-H* signal in the solution intensified, confirming that Fe(II) is the primary reductive source for H* generation. However, as the MoS_2_ surface S-vacancy concentration increased, the H* signal in the bulk solution continuously weakened. This was not a reduction in H* generation but indicated that a vast amount of H* was effectively intercepted by S-vacancies and transformed from a liquid-phase free state to a surface-adsorbed state, thereby inhibiting its ineffective escape into the bulk solution. This synergistic mechanism of “Fe(II) as generator, S-vacancies as stabilizer” provided a mechanistic basis for H-mediated hydrodechlorination discussed below.

To directly investigate the nature of the trapped hydrogen mentioned in the EPR results, we performed in situ infrared spectroscopy under reaction conditions (Fig. 3d). Several distinct features were observed in the experiment that support the stabilization of hydrogen at the MoS_2_/Fe(OH)_2_ interface. Specifically, at ~1800 cm^−1^, we observed a peak corresponding to hydrated protons (H_3_O^+^)^5^, with its intensity increasing as the MoS_2_ loading was increased. This indicates the role of solvated protons in facilitating proton transfer, thereby enhancing interfacial hydrogen flux and accelerating reaction kinetics. Additionally, at ~2000 cm^−1^, a new peak appeared in the heterojunction samples that was absent in pure Fe(OH)_2_. This peak corresponds to metal-hydrogen (Mo-H) interactions^5,44^, suggesting that H* is stabilized at Mo sites associated with S vacancies in MoS_2_. The increasing intensity of this peak with MoS_2_ loading further confirms that more H* is trapped at these defect sites. Simultaneously, at ~2500 cm^−1^, we observed a weaker absorption corresponding to S-H vibrations^45,46^, indicating that sulfur sites on MoS_2_ provide additional adsorption sites for H*. In summary, these in situ infrared spectroscopy results, combined with the EPR findings, provide direct spectroscopic evidence for the presence of surface-trapped hydrogen, thus supporting the proposed mechanism of effective H* migration and utilization, particularly in deep hydrodechlorination reactions.

### Interfacial geometry and S-vacancies govern the fate of H*

Control experiments showed that pristine Fe(OH)_2_ alone exhibited negligible CF degradation activity (Fig. S47), while a physically mixed Fe(OH)_2_/MoS_2_ sample, prepared with identical composition and dosage as the heterojunction, displayed substantially suppressed CF conversion and almost no CH_4_ formation (Fig. S50). These results establish that simple component addition is insufficient and that tight interfacial coupling is essential for enabling selective deep hydrodechlorination.

Guided by this insight, we systematically investigated how the composition and structural characteristics of the MoS_2_/Fe(OH)_2_ interface govern H* pathways and electron selectivity. In particular, we focus on two independent yet synergistic factors: the interfacial contact geometry, which dictates the spatial accessibility and connectivity of H* transfer pathways, and the S-vacancy density of MoS_2_, which determines the interface’s capacity to capture, stabilize, and mobilize transferable H* via continuous defect-derived electronic states. Together, these parameters define the productive interfacial hydrogen flux and thereby control both electron distribution and the selectivity toward deep CF hydrodechlorination products.

Increasing the loading of either component enhanced interfacial interactions and accelerated electron transfer (Fig. 3e), leading to an overall increase in CF degradation rate. In contrast, the product distribution exhibited pronounced sensitivity to the component ratio. At low MoS_2_/Fe(OH)_2_ ratios, TEM images revealed that Fe(OH)_2_ extensively covered the MoS_2_ surfaces and exhibited severe aggregation (Fig. S15), reflecting degraded interfacial contact geometry. Under these conditions, H* remained largely confined within aggregated Fe(OH)_2_ regions, where short H*-H* distances favored recombination to H_2_ rather than transfer to MoS_2_, leading to a shift in electron selectivity toward hydrogen evolution. When Fe(OH)_2_ loading was further increased, H_2_ electron selectivity remained above 90%, indicating that hydrogen evolution dominates and reduced interfacial coupling limits deep hydrodechlorination, even in the presence of abundant H*. In this scenario, due to the lack of effectively stabilized H*, deep hydrodechlorination was limited, and the products largely remained as the initial electron-transfer intermediates, dichloromethane (DCM), resulting in low methane (CH_4_) yield (Fig. 3f, g).

In contrast, at higher MoS_2_/Fe(OH)_2_ ratios, TEM images showed intimate contact between Fe(OH)_2_ and MoS_2_ surfaces, while MoS_2_ retained ample exposed surface area enriched with coordinatively unsaturated sites (Fig. S14). Correspondingly, post-reaction Fe^3+^ concentrations increased with MoS_2_ loading, suggesting that heterojunction formation enhances H* generation by Fe(OH)_2_, likely due to an elevated Fe^2+^ reduction potential toward protons. Under this configuration, H* could be effectively captured and stabilized by vacancy-rich MoS_2_. The increased population of stabilized H* facilitated interfacial hydrogen transfer, promoting deep hydrodechlorination and significantly boosting CH_4_ electron selectivity while maintaining low DCM selectivity (Fig. 3f, g).

Having established the geometric requirements for efficient H* transfer, we next isolated the role of S-vacancy density at fixed Fe(OH)_2_ loading (1.5 mM). When varying MoS_2_ defect density, post-reaction Fe^3+^ concentrations remained nearly unchanged, indicating that the intrinsic H* generation capacity of Fe(OH)_2_ is essentially constant. Interestingly, as shown in Fig. S51, at low S-vacancy concentrations (0%), H_2_ production increased noticeably with MoS_2_ loading, whereas at high S-vacancy densities (6–9%), H_2_ evolution was largely insensitive to MoS_2_ content. This suggests that the excess H* is effectively stabilized by MoS_2_ S-vacancies. We attribute this behavior to two main factors: (i) at high MoS_2_ loading, Fe(OH)_2_ aggregation is greatly reduced, mitigating confinement of H* within aggregated domains where short H*-H* distances favor recombination; (ii) Increasing the S-vacancy density alleviates electron accumulation at the interface, facilitates the subsequent migration of H*, and provides stabilized H* sites.

**Fig. 4:**
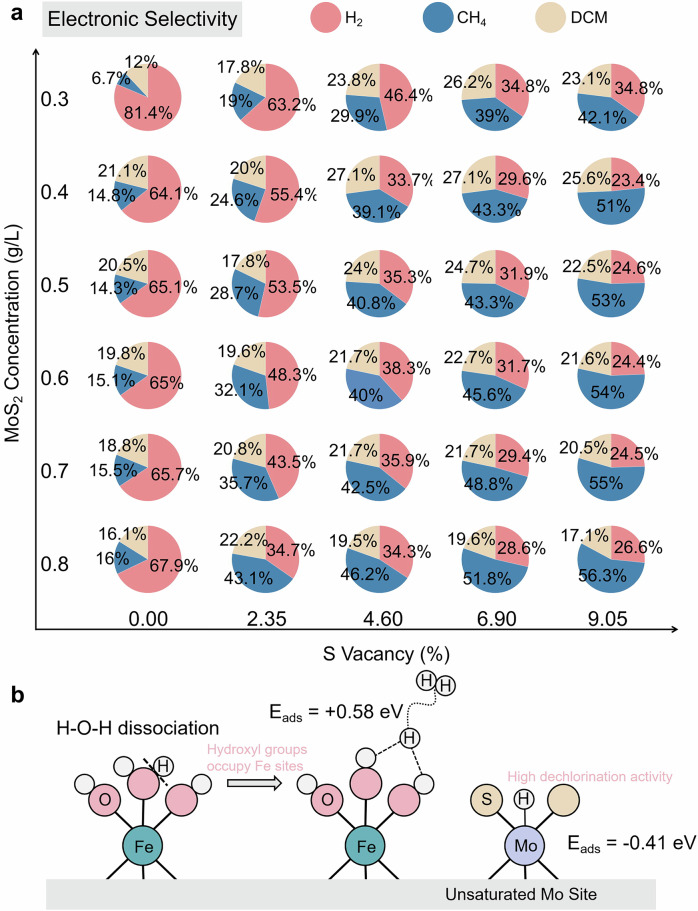
Product selectivity and site-dependent H* adsorption energetics. **a** Electron selectivity distributions for CH_4_, DCM and H_2_ under different reaction conditions, shown as pie charts. Each pie chart represents one reaction condition, and each sector indicates the electron selectivity toward the corresponding product. **b** Calculated H* adsorption energies at hydroxyl sites on Fe(OH)_2_ and S-vacancy sites on MoS_2_. Source data are provided in Source Data 1.

All experimental groups with the same sulfur-vacancy concentrations were plotted as a unified box plot, and the results (Fig. 3h) showed that increasing sulfur-vacancy density generally accelerates chloroform hydrodechlorination kinetics. The response plot of k_1_ as a function of sulfur-vacancy concentration under the same conditions (Tables S3–S7) further supported an overall monotonic dependence within the investigated vacancy range (0–9%), with a saturation-like tendency at higher vacancy densities in a small subset of experimental groups (Fig. S46). Electronic selectivity results showed that, when the MoS_2_ concentration is 0.8 g/L, as the sulfur-vacancy concentration increases, H_2_ electron selectivity suppressed from 67.9 to 26.6%, while CH_4_ selectivity increased from 16.0 to 56.3% and DCM selectivity showed only minor fluctuations, increasing slightly from 16.1 to 22.2% before decreasing to 17.1%. (Fig. 4a). These findings indicate that the primary function of S-vacancies is not merely to accelerate the overall reaction rate, but to inhibit electron diversion to hydrogen evolution, thereby redirecting reducing H* toward deep hydrodechlorination. This trend is further supported by adsorption-energy calculations (Fig. 4b). H* adsorption on OH sites of Fe(OH)_2_ is energetically unfavorable (+0.58 eV), whereas H* binds strongly at S-vacancy sites on MoS_2_ (−0.41 eV), providing a thermodynamic driving force for directional H* migration from Fe(OH)_2_ toward MoS_2_. The high affinity of H* for vacancy sites facilitates its stabilization and transfer at the interface, ensuring that reactive hydrogen is efficiently utilized for deep hydrodechlorination and contributing to the observed high CH_4_ electron selectivity.

Collectively, these observations demonstrate that the interplay between interfacial contact geometry and S-vacancy density governs the fate of interfacial H*, establishing the necessary conditions for selective H* transfer and deep CF dechlorination.

### Vacancy-induced shift from SET to HAT

Upon formation of the MoS_2_/Fe(OH)_2_ heterojunction, interfacial charge transfer, as well as the reduction of protons by Fe^3+^ during the reaction, can partially oxidize Fe^2+^ and may enable intervalence charge transfer between Fe^2+^ and Fe^3+^^32,47,48^, thereby enhancing the overall reducing capability of the Fe(OH)_2_ phase. Under these conditions, CF reduction can proceed via two mechanistically plausible routes: a Fe-associated SET pathway, which generates chlorinated radical intermediates (·CCl_3_, ·CHCl_2_) that may diffuse into the bulk solution, and an interfacial HAT pathway, in which stabilized H* exhibits continuous hydrogenation activity, enabling deep hydrodechlorination of CF.

We further demonstrated through mechanistic experiments that S-vacancies play a decisive role in determining the dominant CF reduction pathway. Mechanistic experiments were conducted at a well-dispersed Fe(OH)_2_ concentration of 1.5 mM and a MoS_2_ concentration of 0.8 g/L, ensuring strong heterojunction coupling and accessible S-vacancy sites.

Radical-trapping experiments provided the first indication of a vacancy-dependent shift. Following initial electron transfer, CF forms chlorinated radicals such as ·CCl_3_ and ·CHCl_2_. If these intermediates are not rapidly intercepted at the interface, they may escape into the bulk solution, where they can be captured by 2,3-dimethyl-2-butene (DMB)^49^. At high S-vacancy concentrations, the system contains a larger population of stabilized H*, resulting in lower concentrations of partially dechlorinated intermediates. Under these conditions, the HAT pathway dominates over SET, promoting sequential hydrogenation and deep CF dechlorination (Fig. S21).

**Fig. 5:**
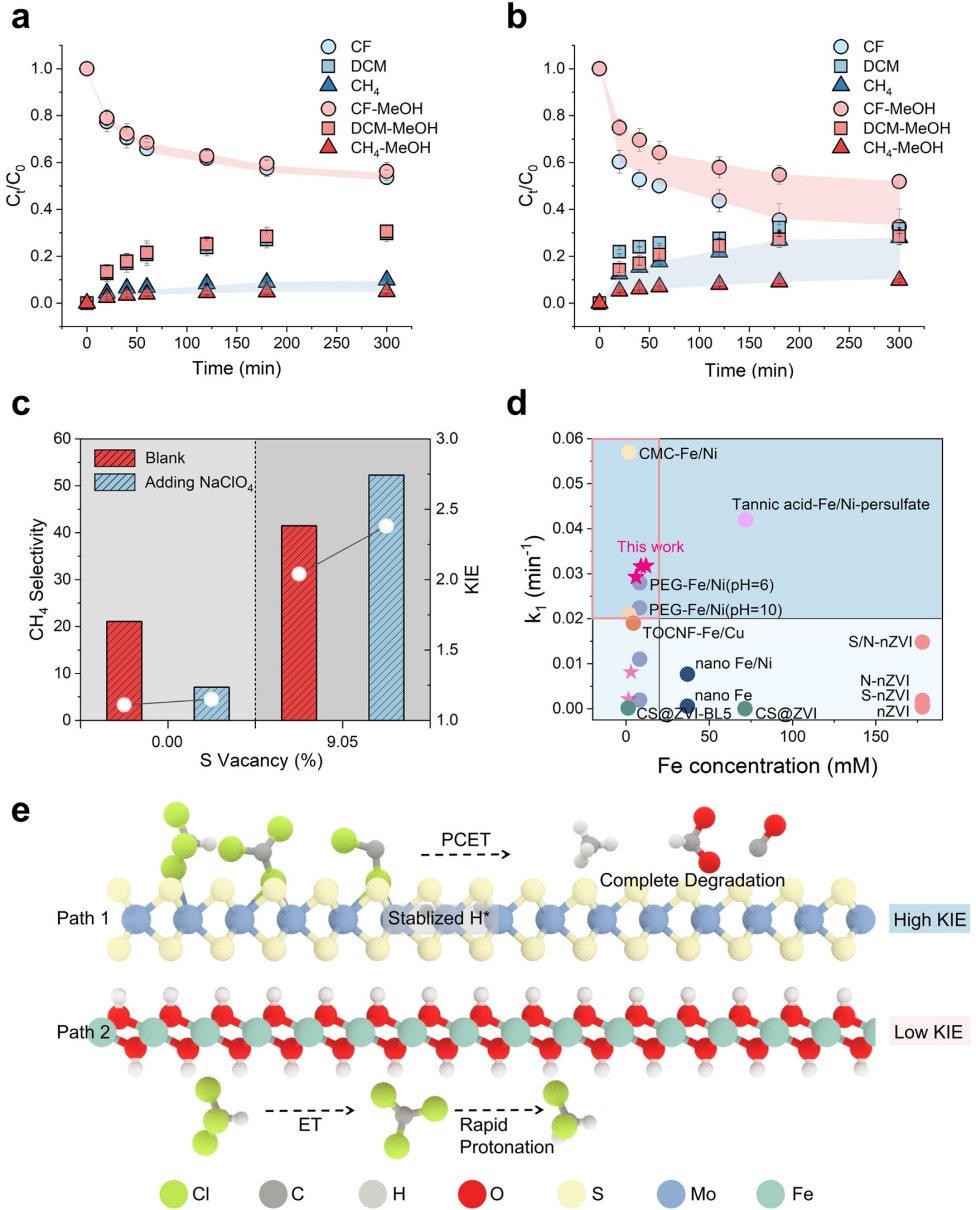
Dual-pathway regulation of CF hydrodechlorination on MoS_2_/Fe(OH)_2_. **a**, **b** Methanol quenching experiments over MoS_2_/Fe(OH)_2_ catalysts with low and high S-vacancy density, respectively. The red shaded areas indicate the decrease in CF degradation, and the blue shaded areas indicate the decrease in CH_4_ production after methanol addition. **c** CH_4_ selectivity and kinetic isotope effect (KIE) with and without NaClO_4_. **d** Performance comparison of the MoS_2_/Fe(OH)_2_ catalyst, marked by the red star, with previously reported iron-based systems. The red box denotes the performance-advantaged region, corresponding to lower Fe consumption and higher CF degradation rate constants (k_1_). Comparison data were taken from refs. ^55–61^. **e** Schematic diagram of the competitive dual pathways on the MoS_2_/Fe(OH)_2_ surface. In the structural model, grass green, dark gray, light gray, red, yellow, blue and teal represent Cl, C, H, O, S, Mo and Fe atoms, respectively. Source data are provided in Source Data 1.

Methanol-quenching experiments further probed the functional role of H*^50^. At low S-vacancy concentration (0.00%), the addition of 1 M methanol only slightly affected CF degradation and product distribution: CF degradation decreased from 46.4 to 43.6%, CH_4_ yield from 9.8 to 4.9% and selectivity from 21.1 to 11.2% (Fig. 5a, b). By contrast, at high S-vacancy concentration (9.05%), methanol addition caused a much stronger suppression, decreasing CF degradation from 67.4 to 48.2%, CH_4_ yield from 28.0 to 9.7% and selectivity from 41.5 to 20.0%. These results indicate that CF reduction in the high-vacancy system relies strongly on interfacial H*, particularly for the deep hydrodechlorination steps leading to CH_4_ formation. In contrast, under low-vacancy conditions, the limited availability of transferable H* leaves the reaction closer to an SET-dominated regime.

Electrolyte modulation and kinetic-isotope-effect (KIE) measurements further supported this mechanistic transition. NaClO_4_ perturbs the interfacial double-layer and hydrogen-bonding environment^51,52^, affecting interfacial charge transfer and solvent reorganization at the hydroxyl-terminated Fe(OH)_2_ surface. As a layered hydroxide, Fe(OH)_2_ relies on its surface hydrogen-bond network to regulate local reorganization energy and gate interfacial electron transfer^53,54^. Consequently, Fe(OH)_2_-dominated SET processes are expected to be particularly sensitive to NaClO_4_ addition. At low S-vacancy concentration, NaClO_4_ decreased the apparent rate constant (k_1_) from 0.00165 to 0.00120 min^−1^, while the KIE increased only slightly from 1.11 to 1.15 (Fig. 5c). This weak isotope sensitivity indicates that hydrogen transfer contributes little to the rate-limiting step, consistent with a regime dominated by SET. In contrast, at high S-vacancy concentration, NaClO_4_ also lowered the k_1_ (0.00298 to 0.00234 min^−1^), but the KIE increased markedly from 2.04 to 2.38 and was accompanied by enhanced CH_4_ selectivity. These observations suggest that electrolyte-induced perturbations preferentially weaken Fe(OH)_2_-associated electron transfer, whereas the H*-mediated pathway remains comparatively robust. As a result, although the overall rate decreased, the relative contribution of HAT increased, promoting deeper hydrodechlorination.

Taken together, radical trapping, methanol quenching and KIE analyses consistently reveal a vacancy-induced rebalancing of the reaction network. Increasing S-vacancy density does not merely accelerate CF degradation; rather, it promotes the capture, stabilization and utilization of interfacial H*, suppresses radical escape and hydrogen evolution, and progressively shifts the dominant reaction regime from Fe(II)-associated SET toward interfacial H*-mediated HAT (Fig. 5e). This mechanistic transition provides the basis for the unusually high CH_4_ selectivity of the MoS_2_/Fe(OH)_2_ heterojunction and underscores defect-regulated hydrogen management as a general design principle for selective reductive transformations in aqueous environments.

Finally, we compared MoS_2_/Fe(OH)_2_ with some previously reported iron-based materials^55–61^. Benchmarking (Fig. 5d) positions the MoS_2_/Fe(OH)_2_ heterojunction among catalysts that combined low Fe input with high apparent kinetics. Beyond activity, the interface/defect architecture improved electron utilization efficiency by suppressing hydrogen evolution and enhancing electron selectivity toward deep hydrodechlorination products, providing a transferable design principle for selective hydrogen-mediated transformations.

We have demonstrated that the long-standing kinetic trade-off between interfacial hydrogen stabilization and hydrogen mobility can be rationally overcome through defect-mediated electron-hydrogen coupling. By engineering sulfur vacancies to induce delocalized hybridized states rather than localized charge traps, efficient delivery and utilization of turnover-ready H* is sustained even in the presence of an elevated Schottky barrier. This barrier-bypassing interfacial transport enables a decisive shift in reaction selectivity from SET to HAT, while simultaneously suppressing hydrogen evolution. More broadly, this strategy of balancing active hydrogen stabilization with mobility establishes a general design principle for steering hydrogen-mediated pathways in complex heterogeneous catalytic systems. To further enhance the practical relevance of our findings, future work will focus on coupling defect-engineered MoS_2_ with more air-stable hydrogen-generating materials, expanding the applicability of this approach for real-world, large-scale catalytic processes.

## Methods

### Chemicals and Fe(II) stock solutions

CF, DCM, hydrochloric acid, sodium hydroxide and H_2_O_2_ were purchased from Sinopharm Chemical Reagent Co., Ltd. MoS_2_ and 2,3-dimethyl-2-butene were purchased from McLean, acetonitrile was purchased from Beijing Inoke Technology Co., Ltd., and deuterium oxide was purchased from Energy Chemical. Standard methane and carbon monoxide gases were obtained from Zhongxin Ruiyuan Gas Co., Ltd. Fe(II) stock solutions were prepared by adding approximately 14 g of iron powder to 400 mL of anoxic 1 M HCl. The bottle was sealed, briefly flushed with N_2_ and heated at 60 °C with gentle stirring until hydrogen evolution nearly ceased. After cooling, the Fe(II) solution was stored in an oxygen-free glovebox, and the Fe(II) concentration was determined by the 1,10-phenanthroline method.

### Catalyst preparation and defect engineering

Defect-engineered MoS_2_/Fe(OH)_2_ heterojunctions were prepared under oxygen-controlled conditions. MoS_2_ was first treated with H_2_O_2_ solutions of different concentrations (0, 1, 2, 5 and 10 M) for 60 s to introduce sulfur vacancies. The treated MoS_2_ samples were sequentially washed three times with absolute ethanol and ultrapure water, filtered and dried overnight. Fe(OH)_2_ was then deposited onto the MoS_2_ surface by adding the defect-engineered MoS_2_ powder to an Fe(II) solution, adjusting the suspension pH to 8.5 and stirring for 15 min. The resulting catalysts were denoted as MoS_2-x_/Fe(OH)_2-y_-z, where x, y and z represent the MoS_2_ concentration, Fe(OH)_2_ concentration and H_2_O_2_ treatment concentration, respectively.

### Catalytic reactions and kinetic measurements

Chloroform hydrodechlorination was used as a probe reaction to evaluate interfacial hydrogen delivery and utilization. Reactions were performed in airtight headspace glass vials under oxygen-free conditions. In a typical experiment, 5 mL of MoS_2_/Fe(OH)_2_ suspension was transferred into the vial inside a glovebox, followed by addition of 15 μL of CF-saturated aqueous solution at a concentration of 6.6 mM to give an initial CF concentration of 20 μM. The vials were immediately sealed, removed from the glovebox and placed on a rotary shaker at 40 rpm. All reactions were conducted in the dark at 25 °C. Samples were collected at 20, 40, 60, 120, 180 and 300 min for kinetic and product analysis. Pseudo-first-order rate constants were obtained by fitting the time-dependent CF concentration profiles.

### Product analysis and electron selectivity

CF degradation and product formation were quantified by chromatographic analysis using corresponding standards for calibration. Electron selectivity was calculated from the fraction of total electrons consumed in the formation of each product. The stoichiometric electron numbers used for methane, dichloromethane and hydrogen were 6, 2 and 2, respectively. Radical-trapping experiments were performed using 2,3-dimethyl-2-butene (DMB) as the spin trap for trichloromethyl radicals. For these experiments, the MoS_2_/Fe(OH)_2_ suspension was concentrated tenfold by centrifugation at 12,000 rpm for 5 min, and the supernatant was replaced with anaerobic acetonitrile. DMB and CF were then added at concentrations of 200 mM and 10 mM, respectively. After 1 h, the mixture was filtered through a 0.22 μm membrane and analyzed by GC/MS to detect the ·CCl_3_ adduct.

### Interfacial characterization and mechanistic probing

The structure, composition and interfacial properties of the catalysts were characterized by X-ray diffraction, zeta-potential analysis, transmission electron microscopy, Mössbauer spectroscopy, Fourier-transform infrared spectroscopy, Raman spectroscopy, EPR and X-ray absorption spectroscopy. X-ray diffraction patterns were collected using Cu Kα radiation. Transmission electron microscopy samples were prepared by drop-casting 20 μL of suspension onto copper grids and drying them in a glovebox. Electrochemical measurements were performed using a CHI 630 workstation with a glassy carbon working electrode, Pt plate counter electrode and Ag/AgCl reference electrode in 30 mL of deoxygenated 0.5 M Na_2_SO_4_ electrolyte. EPR measurements were conducted using DMPO as the spin-trapping agent, and the Q factor was kept constant for quantitative comparison. Fe K-edge XAFS spectra were collected at the Shanghai Synchrotron Radiation Facility and processed using Athena. Additional characterization details are provided in the Supplementary Information.

### Computational methods

DFT calculations were performed using the Vienna Ab initio Simulation Package with projector-augmented-wave potentials. The exchange-correlation interactions were described using the generalized-gradient approximation with the Perdew-Burke-Ernzerhof functional. The DFT + U method was used for Fe 3*d* electrons with an effective U_eff_ value of 4.0 eV. The plane-wave cutoff energy was set to 450 eV, and Brillouin-zone sampling was performed using a Γ-centered 3 × 3 × 1 k-point mesh. Gaussian smearing with a width of 0.05 eV was used for partial orbital occupancies. The convergence criteria for energy and force were 10^−5^ eV and 0.05 eV Å^−1^, respectively. Four-by-four supercells were constructed for monolayer MoS_2_ and Fe(OH)_2_, and sulfur-vacancy models were generated by selectively removing S atoms from the MoS_2_ surface. A 15 Å vacuum layer was applied along the z direction. Adsorption energies, binding energies, charge-density differences, PDOS and Schottky-barrier heights were calculated to evaluate the relationship between defect-mediated electronic reconstruction and hydrogen stabilization. Optimized DFT model coordinates are provided as Supplementary Data 1.

### Statistical analysis and reproducibility

All replicate measurements were performed in duplicate unless otherwise stated. Data are presented as the mean ± standard error (SE) from two independent measurements where applicable. Error bars indicate SE and were calculated as the standard deviation of the duplicate measurements divided by the square root of the number of measurements (*n* = 2). Box plots were generated by pooling all experimental groups prepared under the same H_2_O_2_ treatment condition; the center line indicates the median, box limits indicate the 25th and 75th percentiles, whiskers indicate 1.5 times the interquartile range and points indicate individual measurements. The number of measurements for each group is provided in the Source Data 1.

## Supplementary information

Supplemantary Information

Description of Additional Supplementary Files

Supplementary Data 1

Transparent Peer Review file

## Source data

Source Data 1

Source Data 2

## Data Availability

The data supporting the findings of this study are available within the article, its Supplementary Information and Source Data files. Optimized DFT model coordinates are provided as Supplementary Data 1. Additional raw data relevant to the study are available from the corresponding author upon request. Source data are provided with this paper.
