# Supplementary material for: Bypassing Schottky constraints via defect-mediated hydrogen transfer in hydrodechlorination
Source: Nat Commun. 2026 Jun 22;17:7861. doi: 10.1038/s41467-026-74751-0 (PMC13443187; doi:10.1038/s41467-026-74751-0)
Supplement: Supplementary file 1 [file 41467_2026_74751_MOESM1_ESM.pdf]

Supporting Information for

Bypassing Schottky Constraints via Defect-Mediated Hydrogen Transfer in Hydrodechlorination

Shunjie Zhu [1], Haoran Chen [2], Zhongjie Cui [1], Shiwei Wang [3], Li-Zhi Huang [1,*]

[1] *School of Civil Engineering, Wuhan University, No. 8, East Lake South Road, Wuhan, Hubei, 430074, China.*

[2] *School of Environmental Science and Engineering, Huazhong University of Science and Technology, No. 1037, Luoyu Road, Wuhan, Hubei, 430074, China.*

[3] *Key Laboratory of Resource Biology and Biotechnology in Western China, Ministry of Education, Provincial Key Laboratory of Biotechnology, College of Life Sciences, Northwest University, 229 Taibai North Road, Xi'an, Shaanxi 710069, China*

*Corresponding Author: Li-Zhi Huang

Email: lizhihuang@whu.edu.cn

Supporting Information Text

Text S1. Chemicals

Chloroform (CF), dichloromethane (DCM), hydrochloric acid, sodium hydroxide and hydrogen peroxide (H_2O_2) were purchased from Sinopharm Chemical Reagent Co., Ltd. (Shanghai, China). The standard methane and carbon monoxide gas was provided by Zhongxin Ruiyuan Gas Co., Ltd. (Wuhan, China). The initial concentration of CF was 20 μM. MoS_2 and 2,3-dimethyl-2-butene were purchased from McLean (Shanghai, China). Acetonitrile was purchased from Beijing inoke Technology Co., Ltd. (Shanghai, China). Deuterium oxide (D_2O) was purchased from Energy Chemical.

Text S2. Preparation of Fe(II) stock solutions[1]

Fe(II) stock solutions were prepared by adding about 14 g of solid iron powder to 400 mL 1 M of anoxic HCl solution. The bottle was capped tightly, briefly flushed with nitrogen (N_2), and then the N_2 inlet needle was removed. The glass bottle was then placed on a magnetic stirrer (stirring at a slow speed) in the fume cupboard and carefully heated at 60°C until the hydrogen almost stops (~2 h). After cooling, store in an oxygen-deprived glovebox. The Fe(II) concentration was determined by 1,10-phenanthroline method.

Text S3. Preparation of $MoS_{2\text{-}x}/Fe(OH)_{2\text{-}y}$-z

Within a strictly oxygen-controlled glovebox, after treating MoS_2 with different concentrations of H_2O_2 (0, 1, 2, 5, 10 M) for 60 seconds, the samples were sequentially washed three times with absolute ethanol and ultrapure water, filtered, and dried overnight in an oven. The MoS_2 samples were labeled as MoS_2-0, MoS_2-1, MoS_2-2, MoS_2-5, and MoS_2-10. A certain concentration of $FeCl_2$ solution was prepared, to which the above MoS_2 powder was added. The pH was adjusted to 8.5, followed by stirring for 15 minutes to deposit $Fe(OH)_2$ on the surface of MoS_2. The final catalyst was denoted as $MoS_{2\text{-}x}/Fe(OH)_{2\text{-}y}$-z, where x represents the concentration of MoS_2 (mM), y represents the concentration of $Fe(OH)_2$ (mM), and z represents the concentration of H_2O_2 (M).

Text S4. Reaction conditions

The reduction of CF by $MoS_2/Fe(OH)_2$ was carried out in airtight glass vials, each experiment was repeated at least twice. First, 5 mL of $MoS_2/Fe(OH)_2$ suspensions were added to headspace glass vials in the glovebox. Then, 15 μL of CF-saturated aqueous solution with a concentration of 6.6 mM was added to the reaction vials to obtain a CF concentration of 20 μM. The vials were immediately capped, taken out from the glovebox and put on a rotary shaker to initiate the reaction. The experiments were performed in darkness, and the rotation speed was 40 rpm. The sampling times were 20, 40, 60, 120, 180 and 300 minutes. All the CF degradation reactions were carried out at a constant temperature of 25 °C.

Text S5. Characterization methods

X-ray diffraction (XRD) patterns were acquired using a PANalytical X'Pert PROX diffractometer (The

Netherlands) with Cu Kα radiation (λ = 0.15418 nm). Suspensions were drop-cast onto clean glass slides and dried in an anoxic glovebox prior to analysis. Scans were performed from 5° to 90° (2θ) at a speed of 10° min^{-1}.

Zeta potential was measured using a Zetasizer (Malvern Panalytical, UK). The sample holder was washed sequentially with anhydrous ethanol and ultrapure water three times in the glovebox, then rinsed with the sample suspension to remove air bubbles, and finally sealed with Parafilm before immediate analysis.

Transmission electron microscopy (TEM) images were captured on a JEM-NEOARM or JEM-ARM200CF microscope (JEOL, Japan). Samples were prepared by drop-casting 20 μL of suspension onto a copper grid and drying in the glovebox.

Electrochemical measurements were performed using a CHI 630 workstation (CH Instruments, China). The sample suspension was coated onto a glassy carbon working electrode, with a Pt plate (2 × 2 cm) and an Ag/AgCl electrode serving as the counter and reference electrodes, respectively. The electrolyte was 30 mL of deoxygenated 0.5 M Na_2SO_4, and the anode-cathode distance was fixed at 10 mm. All tests were conducted inside a glovebox.

Mössbauer spectroscopy was performed in transmission geometry at 77 K using a Wissel MR-2500 spectrometer (Germany) with a 25 mCi $^{57}Co(Rh)$ source. Approximately 30 mg of dried powder was loaded into a holder, flattened, fixed with tape, and sealed in a vacuum bottle inside the glovebox. Spectra were fitted using Recoil software via Lorentzian Multiplet Analysis.

Fourier transform infrared (FTIR) spectra were recorded on a Nicolet 5700 spectrometer. Dried KBr was mixed with the sample powder and ground in a mortar inside the glovebox. The mixture was loaded into a mold, removed from the glovebox, and immediately pressed into pellets for analysis.

Raman spectroscopy was conducted using a HORIBA Jobin Yvon XploRA spectrometer (France) with a 532 nm laser (50–1000 cm^{-1}). Samples were dried in the glovebox and sealed in a vacuum cell to prevent oxidation during transfer.

Electron paramagnetic resonance (EPR) signals were detected using a Bruker A300 spectrometer (Germany). Samples were reacted with 5,5-Dimethyl-1-pyrroline N-oxide (DMPO) (100 mM) for 15 min inside the glovebox, and the mixture was transferred to a Bruker AquaX capillary cell. The Q factor was kept constant for quantitative comparison.

Radical Trapping Experiments

To identify intermediate radicals, 2,3-dimethyl-2-butene (DMB) was employed as a spin trap for trichloromethyl radicals ($\cdot CCl_3$). The $MoS_2/Fe(OH)_2$ suspension was concentrated tenfold by centrifugation (12,000 rpm, 5 min), and the supernatant was replaced with anaerobic acetonitrile. DMB (200 mM) and CF (10 mM) were added to the reaction vial. After 1 h, the mixture was filtered (0.22 μm) and analyzed by GC/MS to detect the $\cdot CCl_3$ adduct ($TCCl_3$, 4,4,4-trichloro-2,3,3-trimethyl-1-butene).

X-ray Absorption Spectroscopy (XAS) Analysis

Fe K-edge X-ray absorption fine structure (XAFS) spectra were acquired in transmission mode at Beamline of the Shanghai Synchrotron Radiation Facility (SSRF). A Si(220) double-crystal monochromator

was detuned to 50% of the maximum intensity to suppress higher-order harmonics. Incident and transmitted X-ray intensities were monitored using ionization chambers. The spectrum of a standard Fe foil was collected simultaneously for energy calibration. Multiple scans were averaged to improve the signal-to-noise ratio. Data processing, including energy calibration (referenced to the first inflection point of the Fe K-edge at 7,112 eV), background subtraction, and normalization, was performed using Athena.

Text S6. Density functional theory (DFT) calculation

All density functional theory (DFT) calculations were performed using the Vienna Ab-initio Simulation Package (VASP)[2] with the projector augmented-wave (PAW)[3, 4] potentials. The exchange-correlation interactions were described by the generalized gradient approximation (GGA)[5] with the Perdew-Burke-Ernzerhof (PBE[6]) functional. To correctly describe the strongly correlated 3d electrons of Fe atoms, the DFT+U method[7] was employed with an effective U_{eff} value of 4.0 eV. The kinetic energy cutoff for the plane-wave basis set was set to 450 eV. The Brillouin zone integration was sampled using a Gamma-centered 3 × 3 × 1 k-point mesh. Partial occupancies of the Kohn-Sham orbitals were allowed using the Gaussian smearing method with a width of 0.05 eV. The convergence criteria for energy and force were set to 10^{-5} eV and 0.05 eV $Å^{-1}$, respectively, during the geometrical optimization and electronic property calculations. For the structural models, 4 × 4 supercells were constructed for both monolayer MoS_2 and $Fe(OH)_2$ to minimize the periodic interaction. To investigate the effect of defect density, four distinct models with varying sulfur vacancy concentrations were created by selectively removing S atoms from the MoS_2 surface. A vacuum layer of 15 Å was applied along the z-direction to prevent interactions between adjacent images. The interaction between the ions and the valence electrons is described by the projector augmented wave method. Adsorption energies (E_{ad}) and binding energies (E_{bd}) were calculated as $E_{ad} = E_{ad/slab} - E_{ad} - E_{slab}$ and $E_{bd} = E_{slab1/slab2} - E_{slab1} - E_{slab2}$, where $E_{ad/slab}$, E_{ad} and E_{slab} and $E_{slab1/slab2}$, E_{slab1}, and E_{slab2} respectively represent total energies for optimized adsorbate/slab system, adsorbate alone and clean substrate in the associated structure and total energies for optimized slab1/slab2 system, slab1 alone and slab2 alone in the heterojunction structure.

Text S7. Calculation of Electron Selectivity

In this study, electron selectivity is defined as the fraction of the total electrons directed towards each product, which is calculated based on the electron consumption of each product (chloroform to methane, chloroform to dichloromethane, and hydrogen). The electron selectivity for each product is then calculated using the following formula:

$$S_i = \frac{Y_i \times n_i}{\sum_i (Y_i \times n_i)}$$

Where:

S_i = Electron selectivity for Product i;

Y_i = Yield of Product i;

n_i = Electrons consumed per molecule of Product i, $n_{methane} = 6$, $n_{dichloromethane} = 2$, $n_{hydrogen} = 2$.

Text S8. The determination of Fe(II) concentration using a phenanthroline method

For the determination of total Fe(II), 1 mL of supernatant of MoS_2/$Fe(OH)_2$ was dissolved in 2 mL HCl (1 M) in the glove box. 100 μL of this solution was transferred to a volumetric flask containing 1.9 mL of phenanthroline solution and diluted 10 times by deionized water, and the total Fe(II) was then determined.

Supporting Information Figures

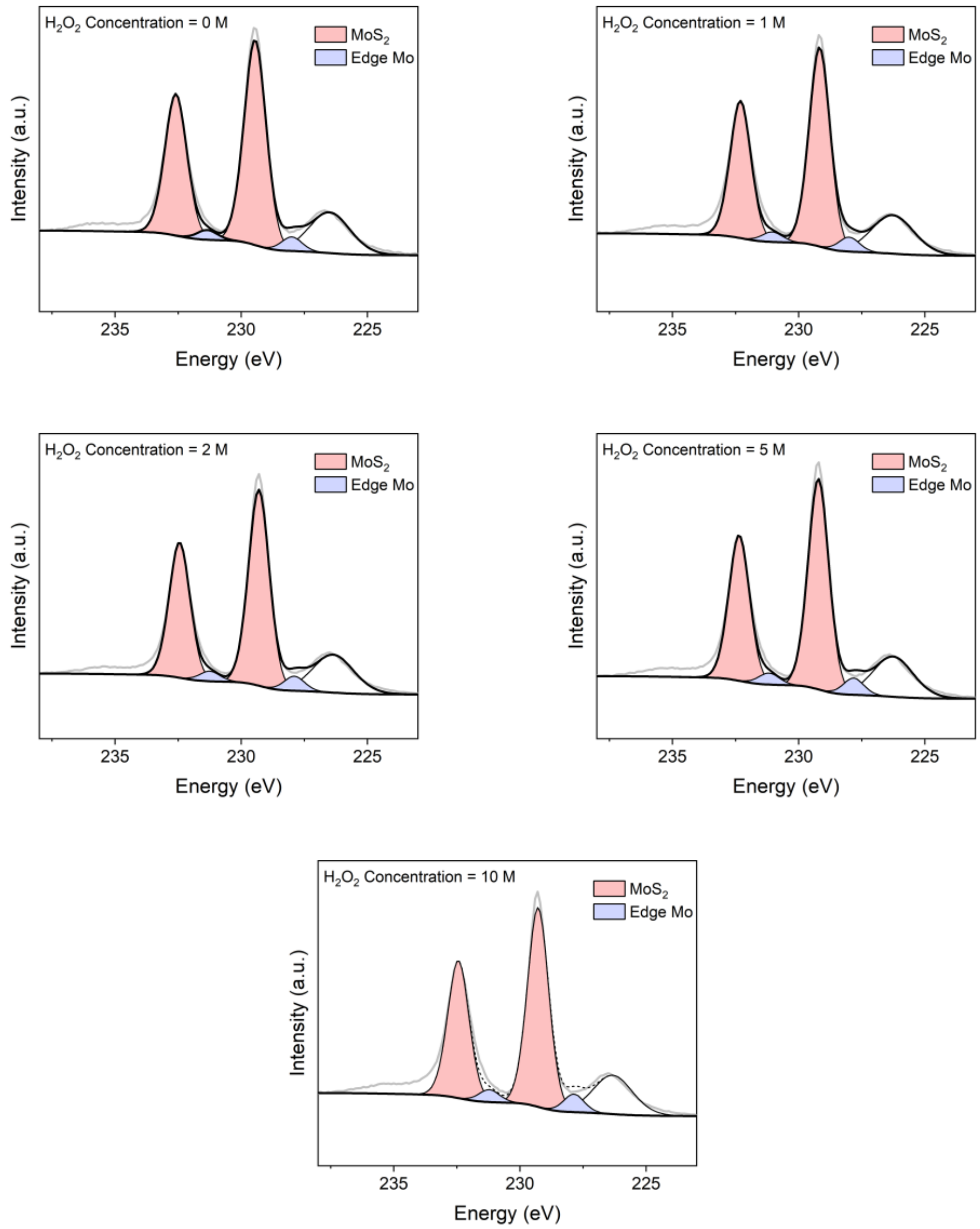

Figure S1. Mo 3d X-ray photoelectron spectroscopy (XPS) spectra of MoS_2 with H_2O_2 etching. The spectra show the evolution of Mo-related chemical states, including Mo^{4+} and edge Mo species [8], after different H_2O_2 treatments.

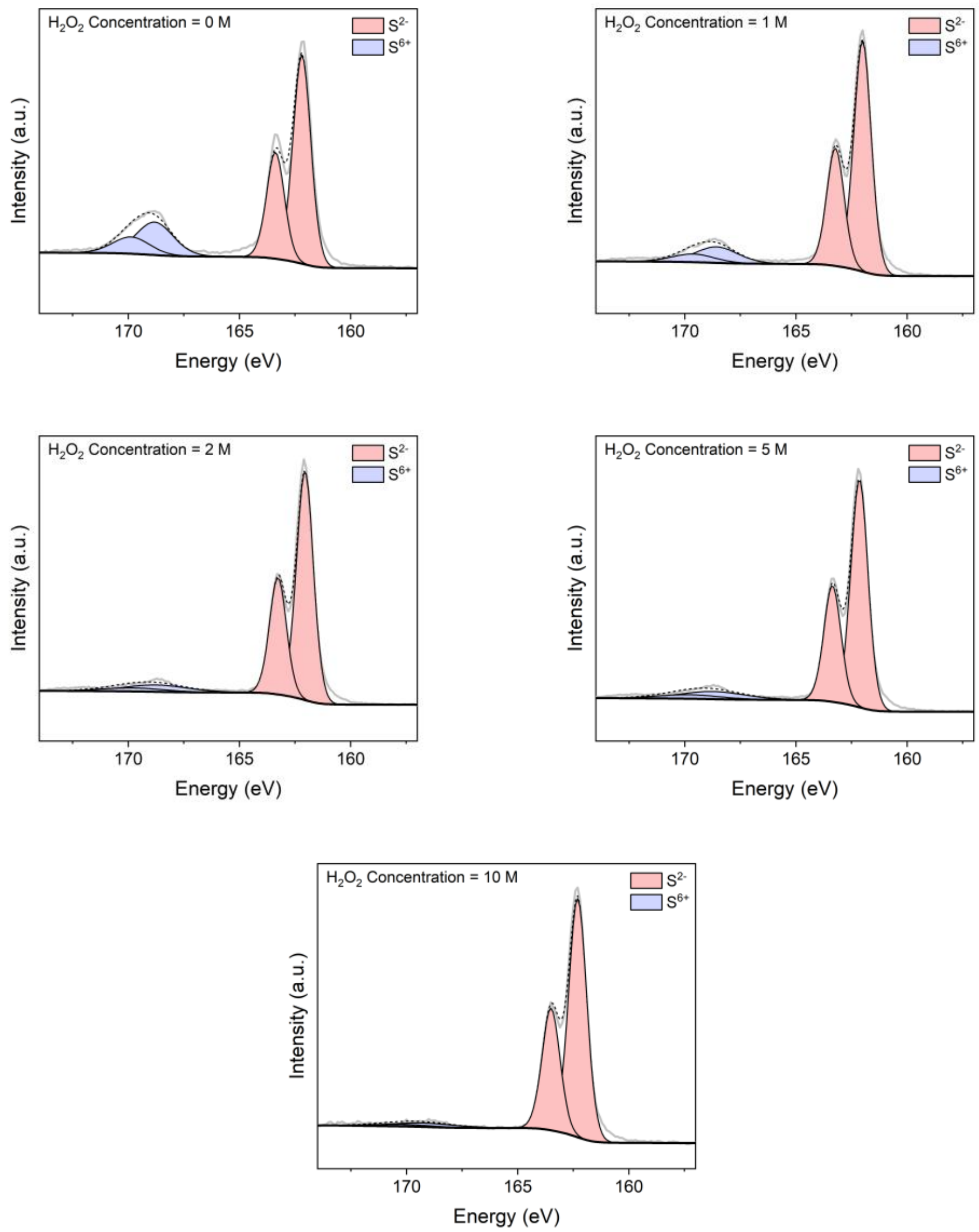

Figure S2. S 2p X-ray photoelectron spectroscopy (XPS) spectra of MoS_2 with H_2O_2 etching (including S^{2-} and edge S^{6+}). The spectra show changes in sulfur-related species, including S^{2-} and oxidized/edge sulfur species, induced by H_2O_2 etching.

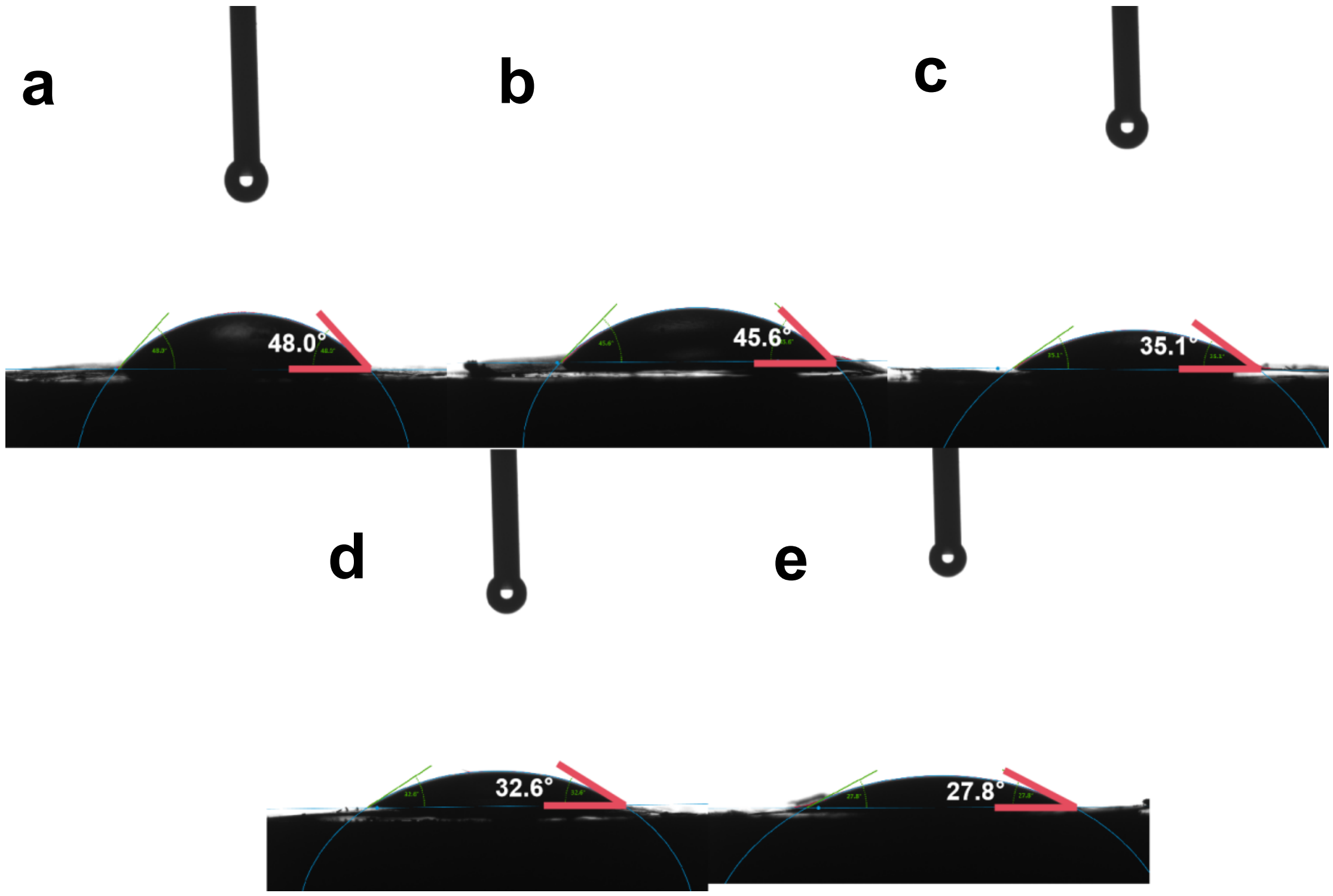

Figure S3. Contact angle images of MoS_2 with increasing H_2O_2 treatment concentration (M). (a) 0, (b) 1, (c) 2, (d) 5 and (e) 10.

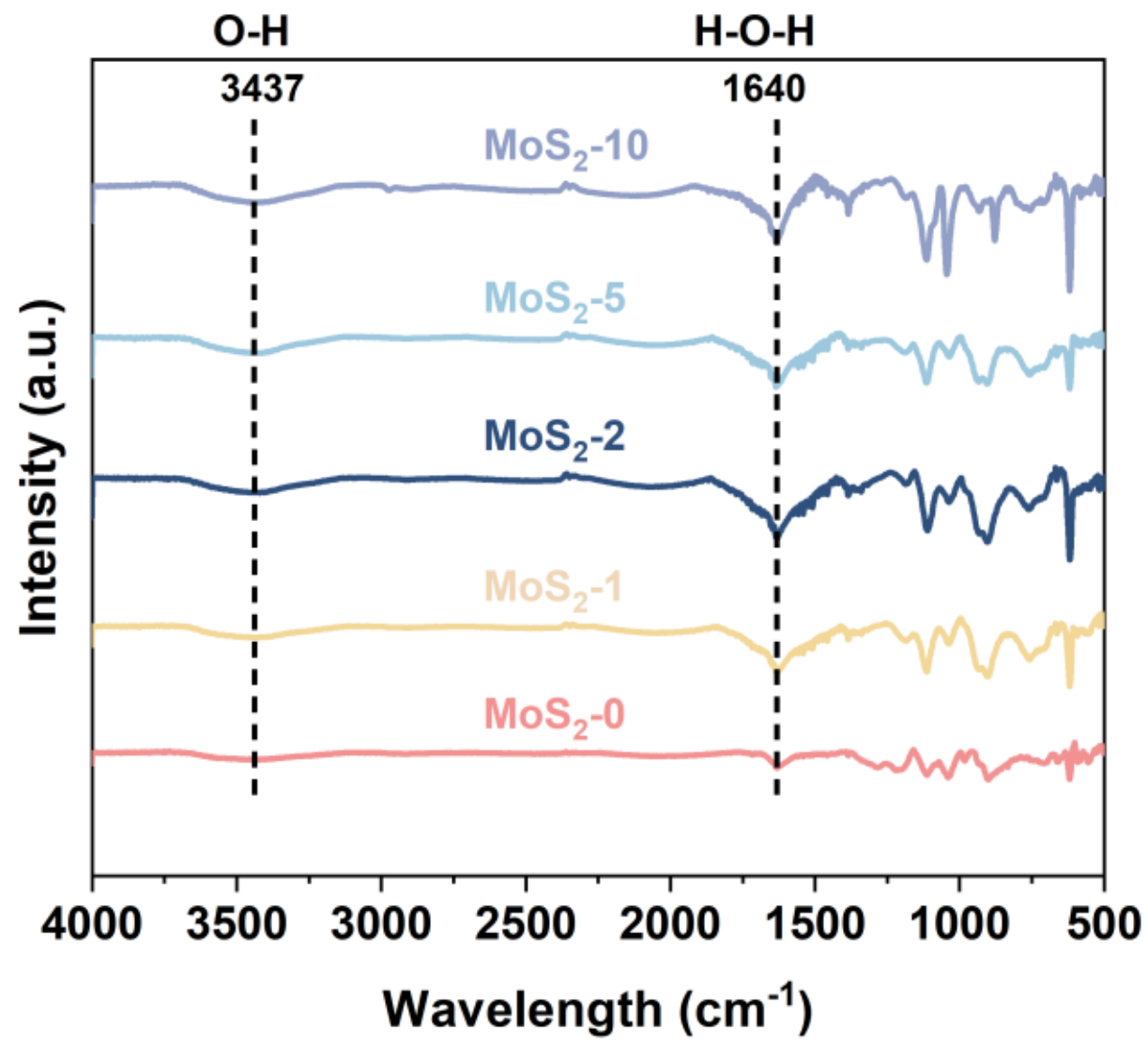

Figure S4. FTIR spectra of MoS_2 with increasing H_2O_2 treatment concentration. The spectra were used to evaluate changes in surface functional groups after progressive oxidative etching.

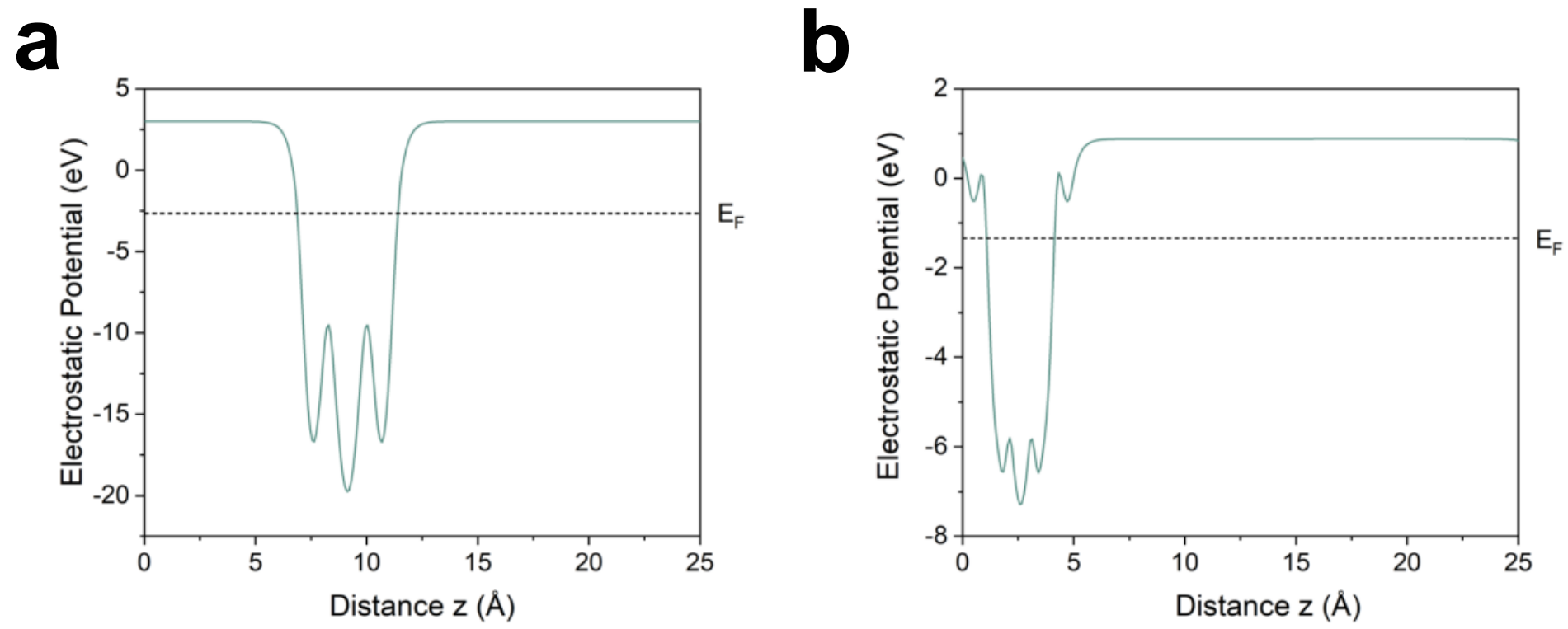

Figure S5. Work function of MoS_2 and $Fe(OH)_2$. Panels a and b show the calculated work functions of MoS_2 and $Fe(OH)_2$, respectively, which were used to evaluate interfacial electronic alignment.

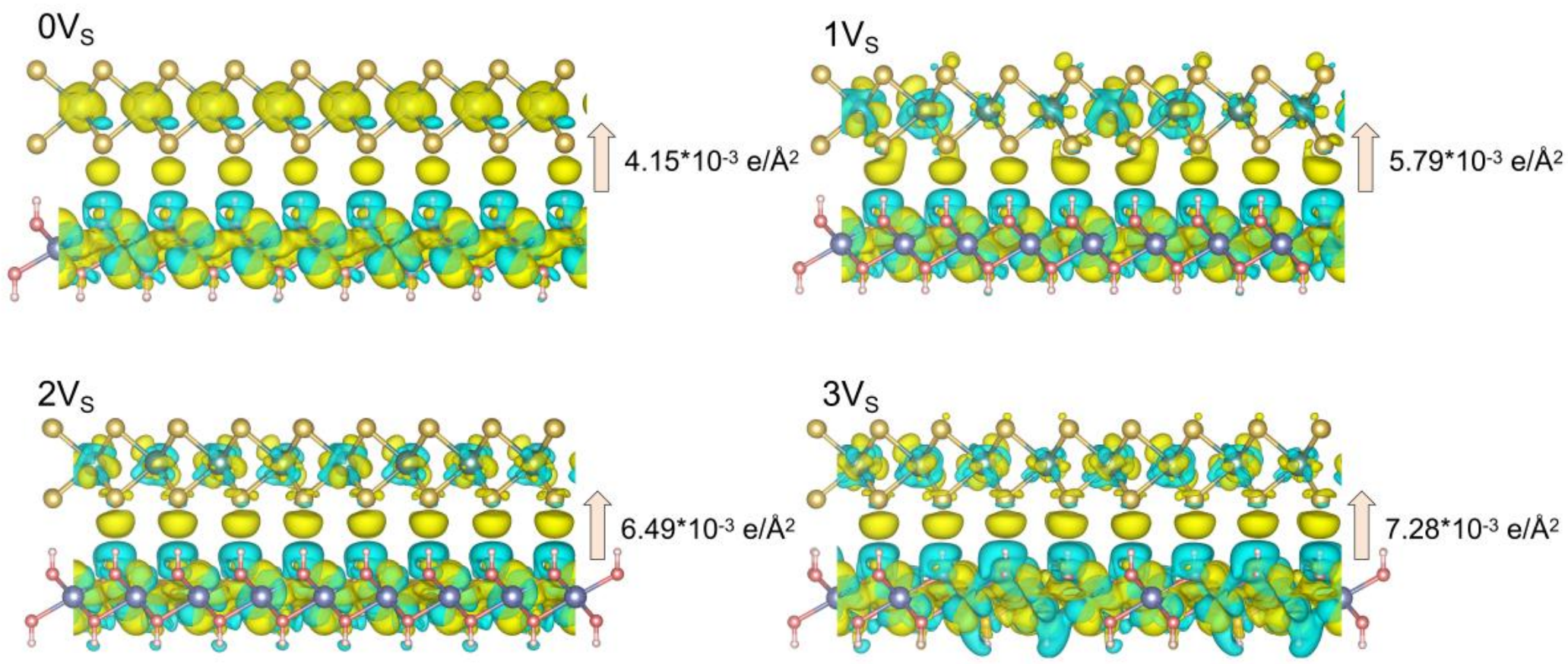

Figure S6. Diagrams of the differential charge density before and after heterojunction formation with different S-vacancy concentrations. Yellow/blue regions indicate electron accumulation and depletion, respectively, revealing interfacial charge redistribution induced by coupling between MoS_2 and $Fe(OH)_2$.

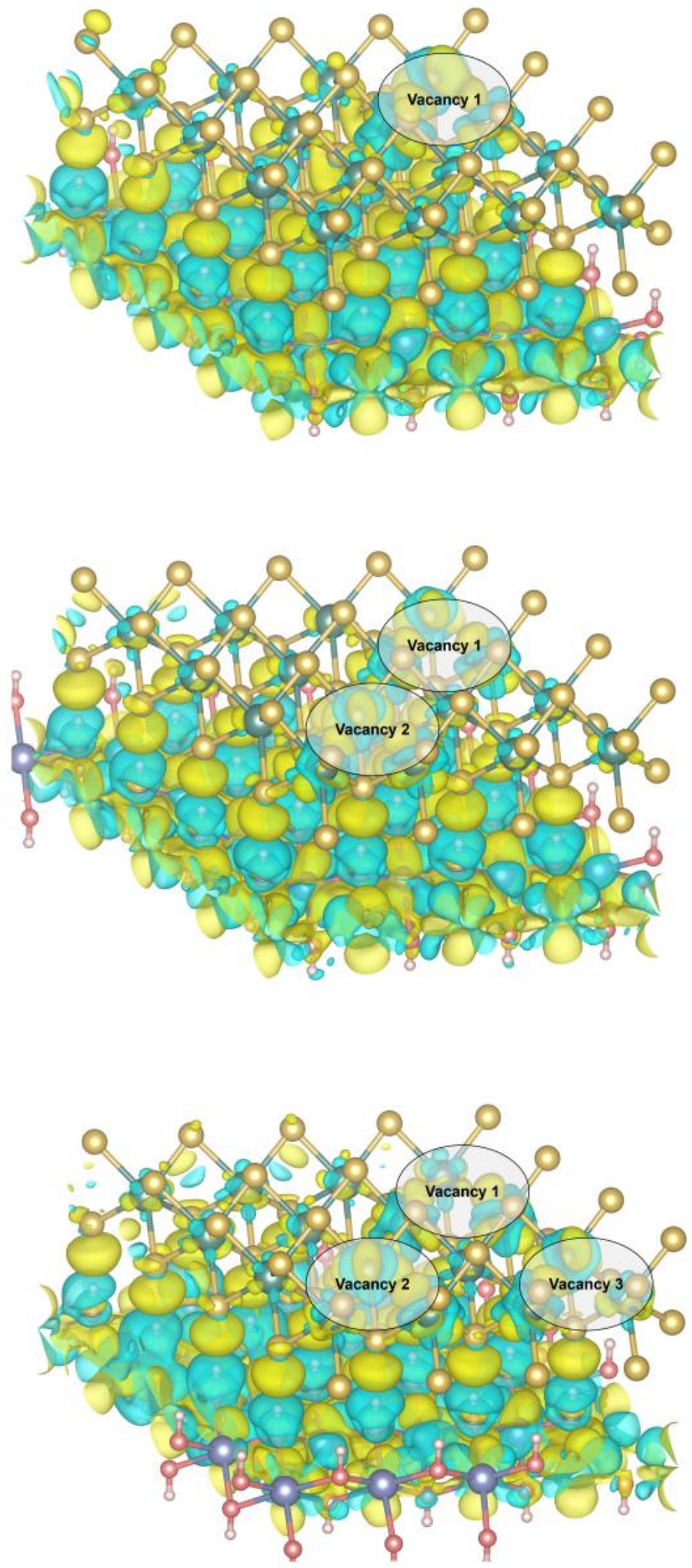

Figure S7. Charge accumulation at the S vacancies after heterojunction formation. Yellow/blue regions indicate electron accumulation and depletion, respectively, showing that S vacancies serve as localized sites for interfacial charge accumulation.

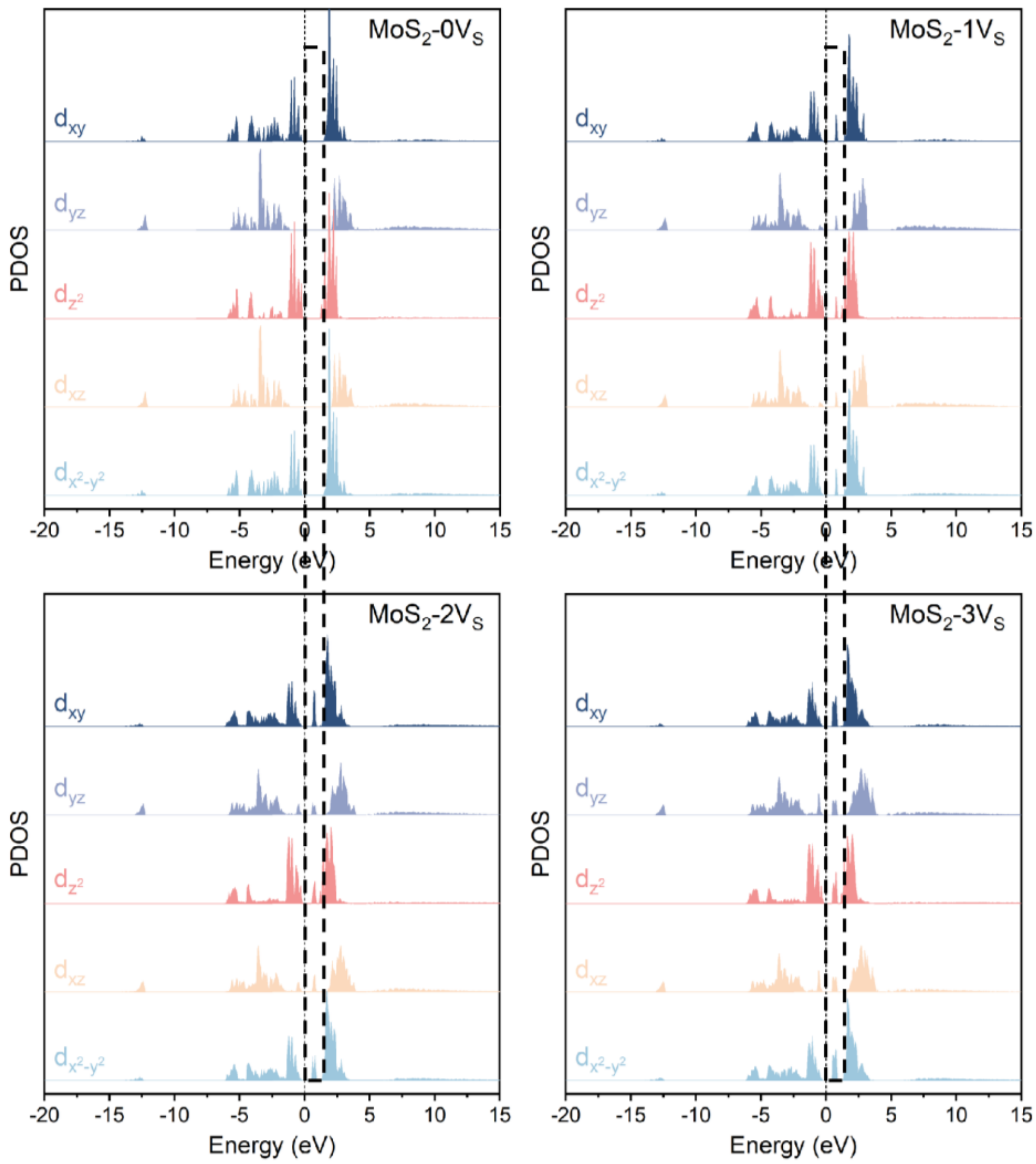

Figure S8. Projected density of states (PDOS) of MoS_2 with different concentrations of S vacancies. The boxed region indicates the defect-level region, highlighting vacancy-induced electronic states near the band-edge region.

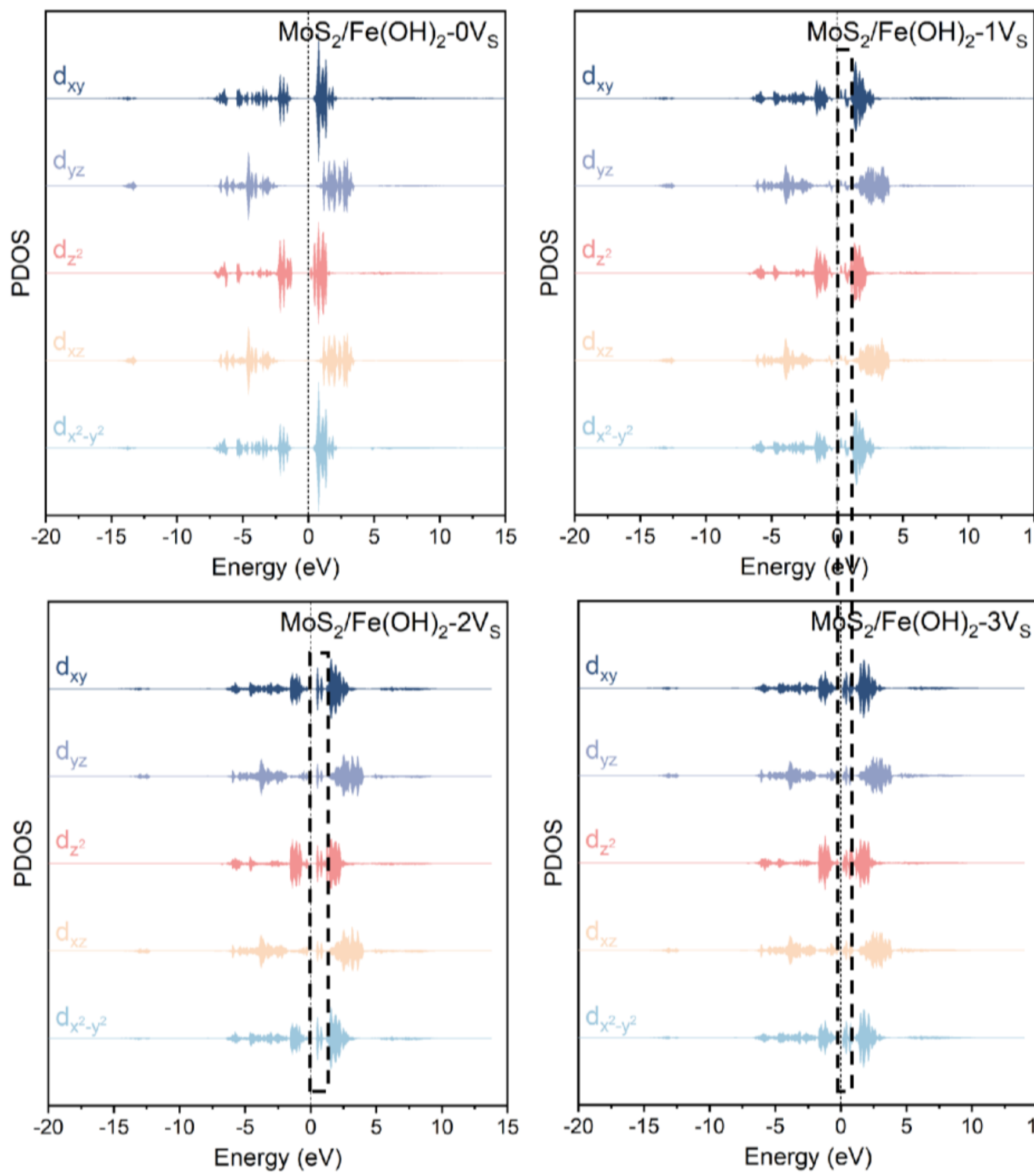

Figure S9. Projected density of states (PDOS) of $MoS_2/Fe(OH)_2$ with different concentrations of S vacancies. The boxed region indicates the defect-level region, highlighting vacancy-related electronic states after heterojunction formation.

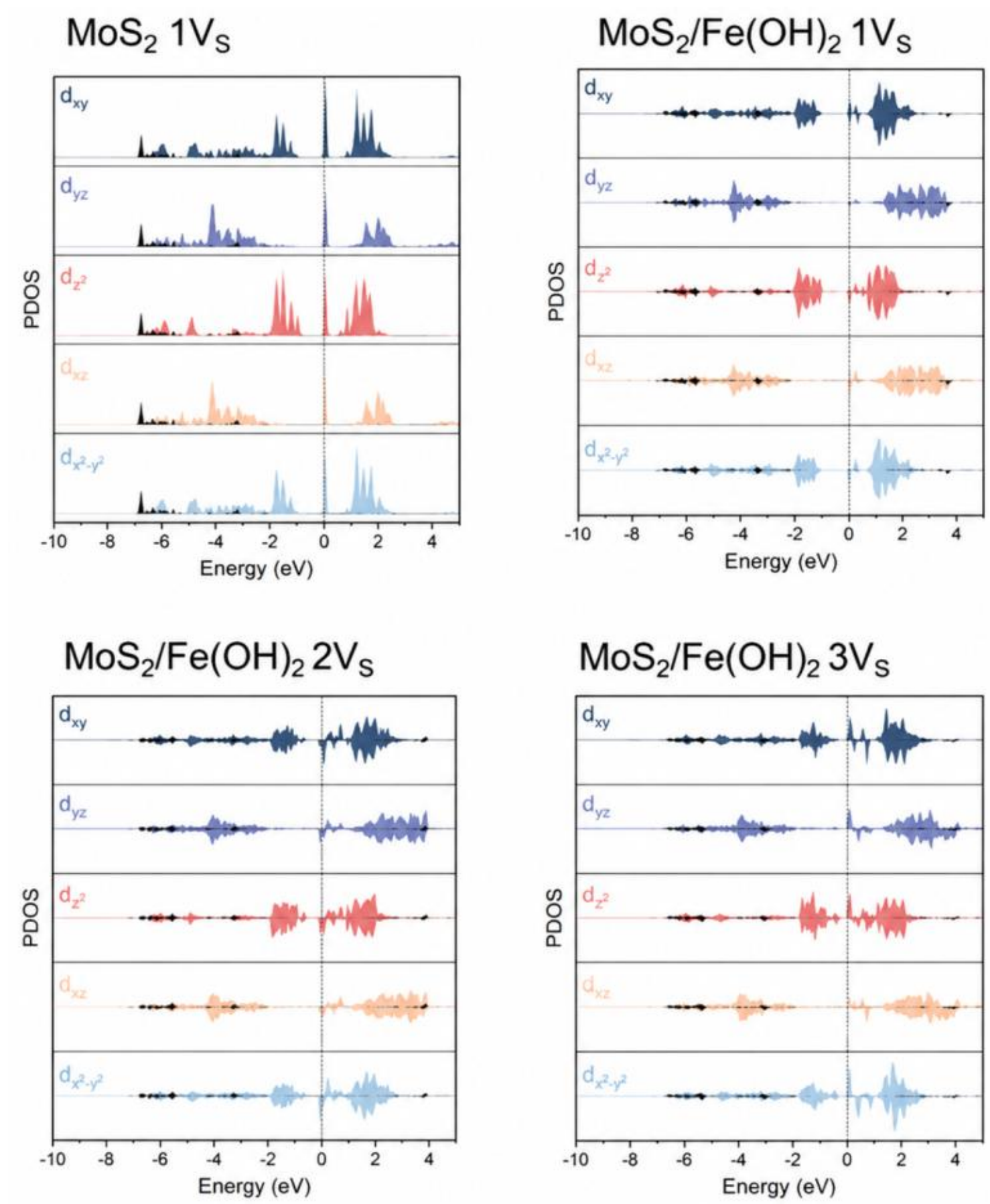

Figure S10. Orbital hybridization of Mo-H. The projected orbital interaction illustrates the electronic coupling between Mo and adsorbed H at the active site.

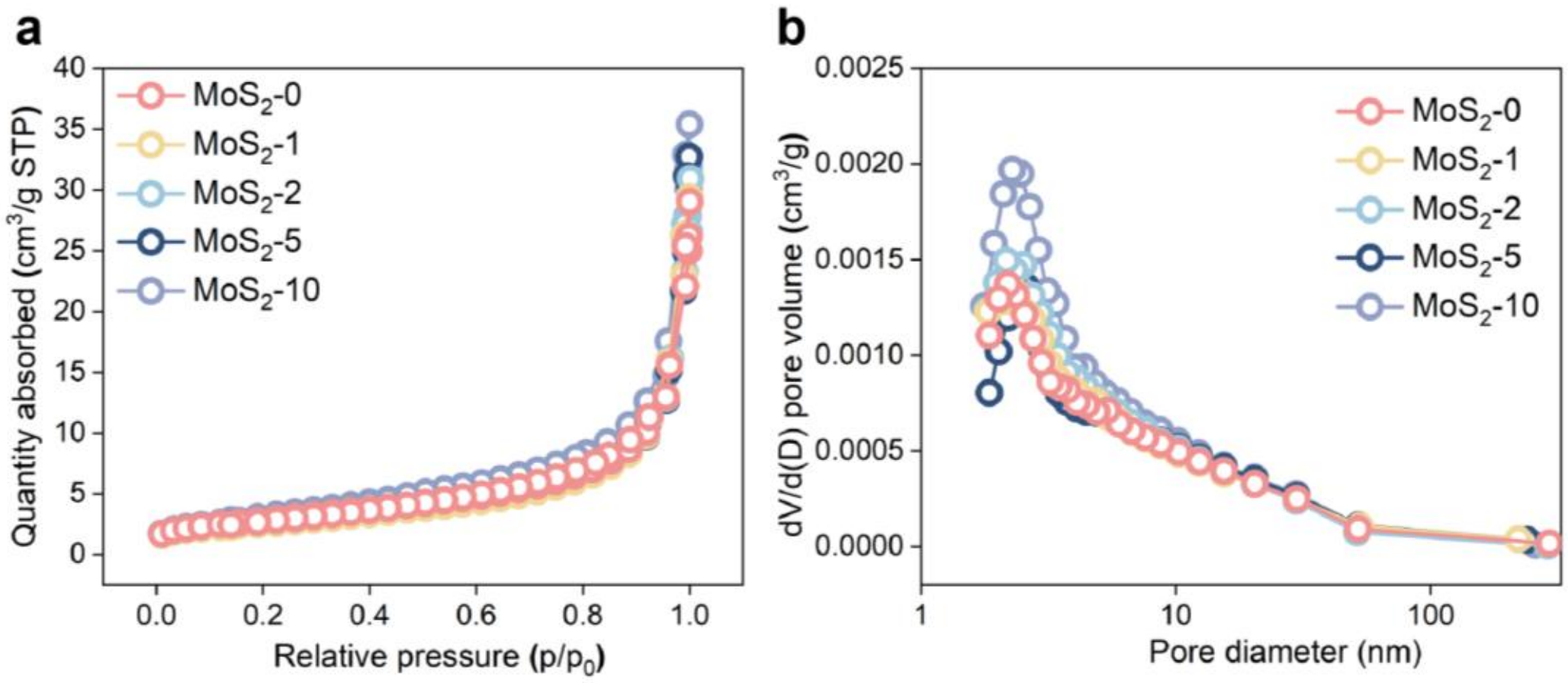

Figure S11. N_2 adsorption-desorption isotherm curve for BET specific surface area analysis of MoS_2-z. (a) BET analysis, (b) BJH pore size distribution.

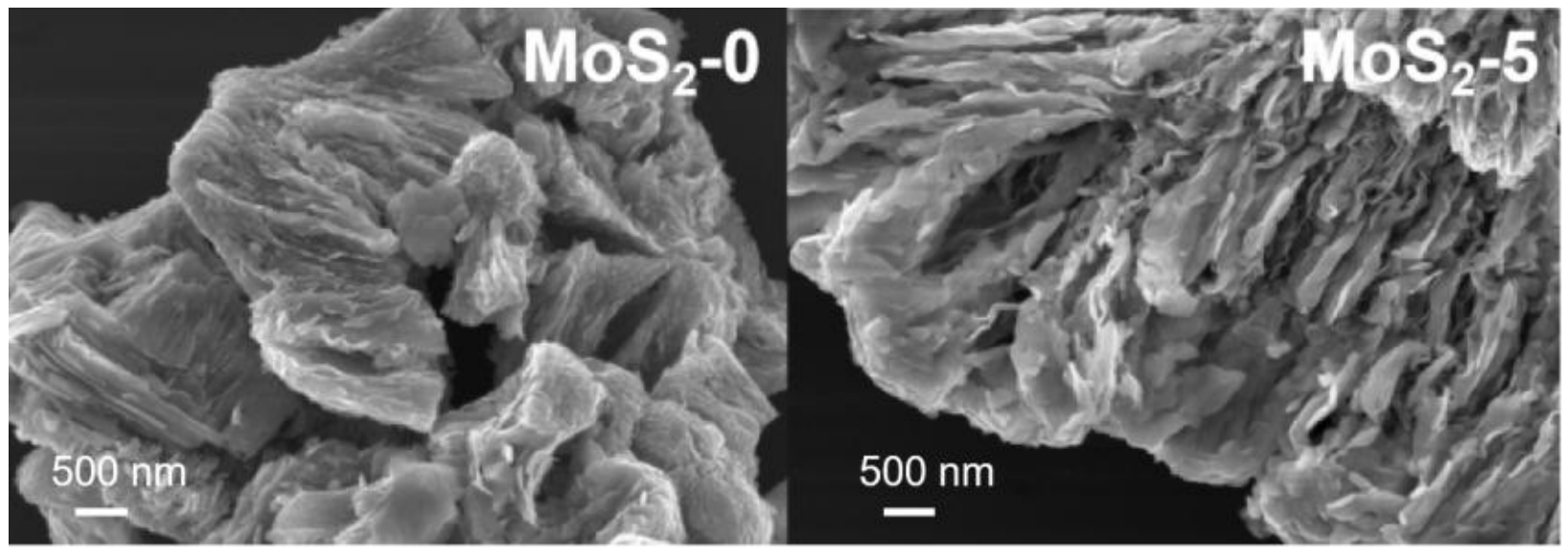

Figure S12. Morphology of MoS_2-0 and MoS_2-5. The images compare the morphology of pristine MoS_2 and H_2O_2-etched MoS_2.

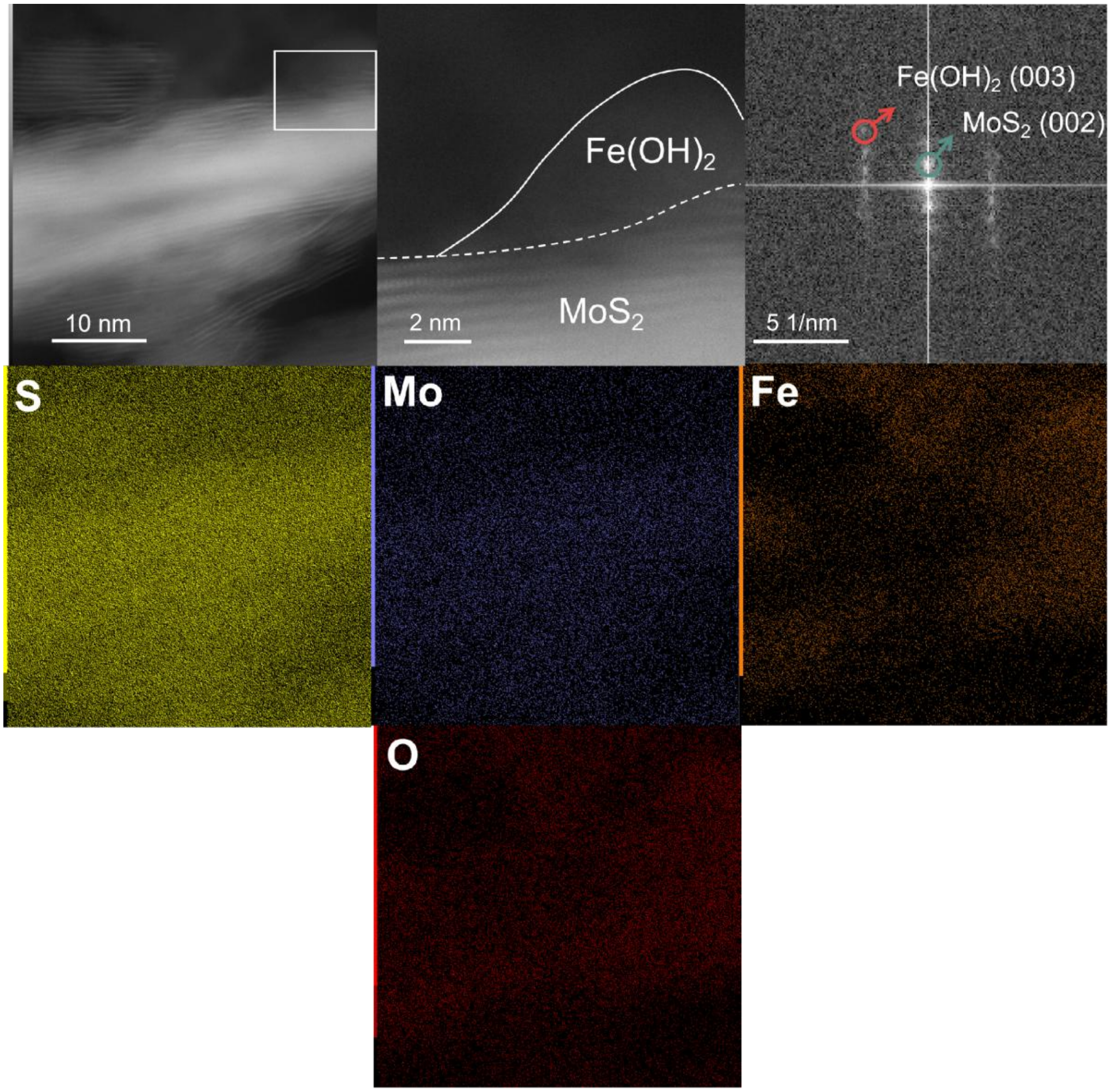

Figure S13. EDS mapping of the $MoS_2/Fe(OH)_2$ interface. The elemental maps show the spatial distribution of Mo, S, Fe and O at the heterojunction interface.

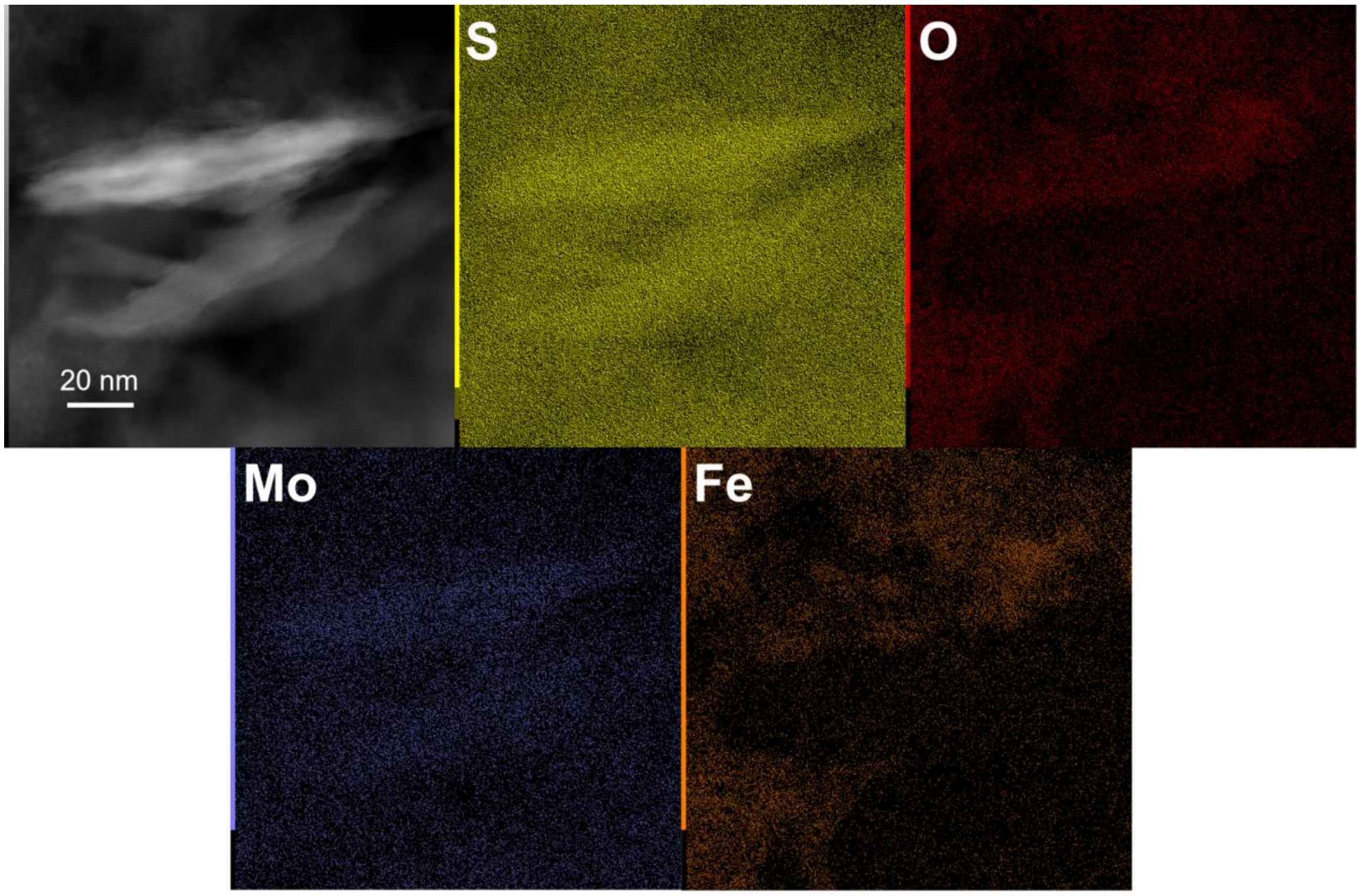

Figure S14. EDS mapping of MoS_2/$Fe(OH)_2$ (high MoS_2 loading). The elemental maps show the spatial distribution of Mo, S, Fe and O at the heterojunction interface.

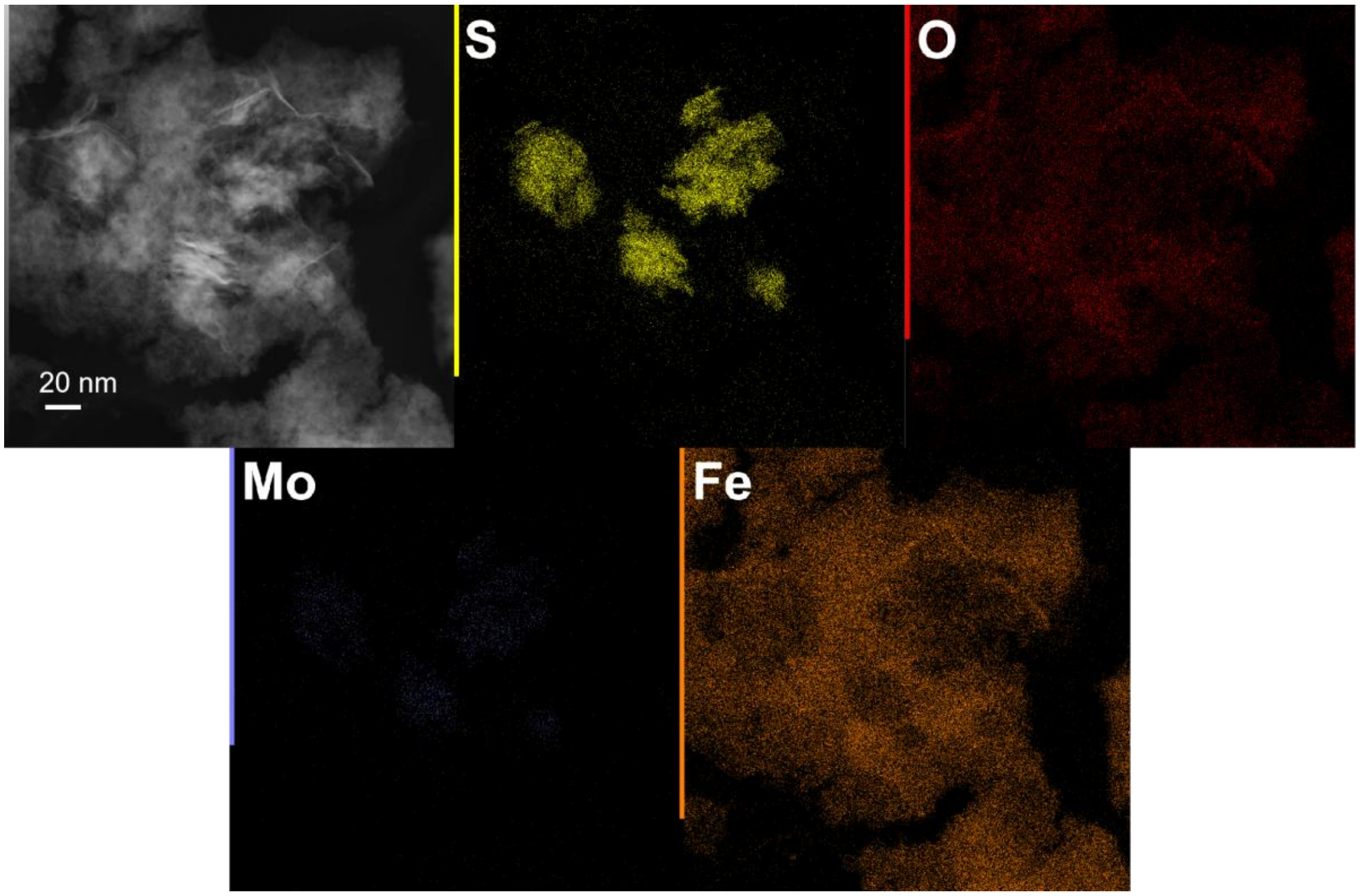

Figure S15. **EDS mapping of MoS_2/$Fe(OH)_2$ (low MoS_2 loading).** The elemental maps show the spatial distribution of Mo, S, Fe and O at the heterojunction interface.

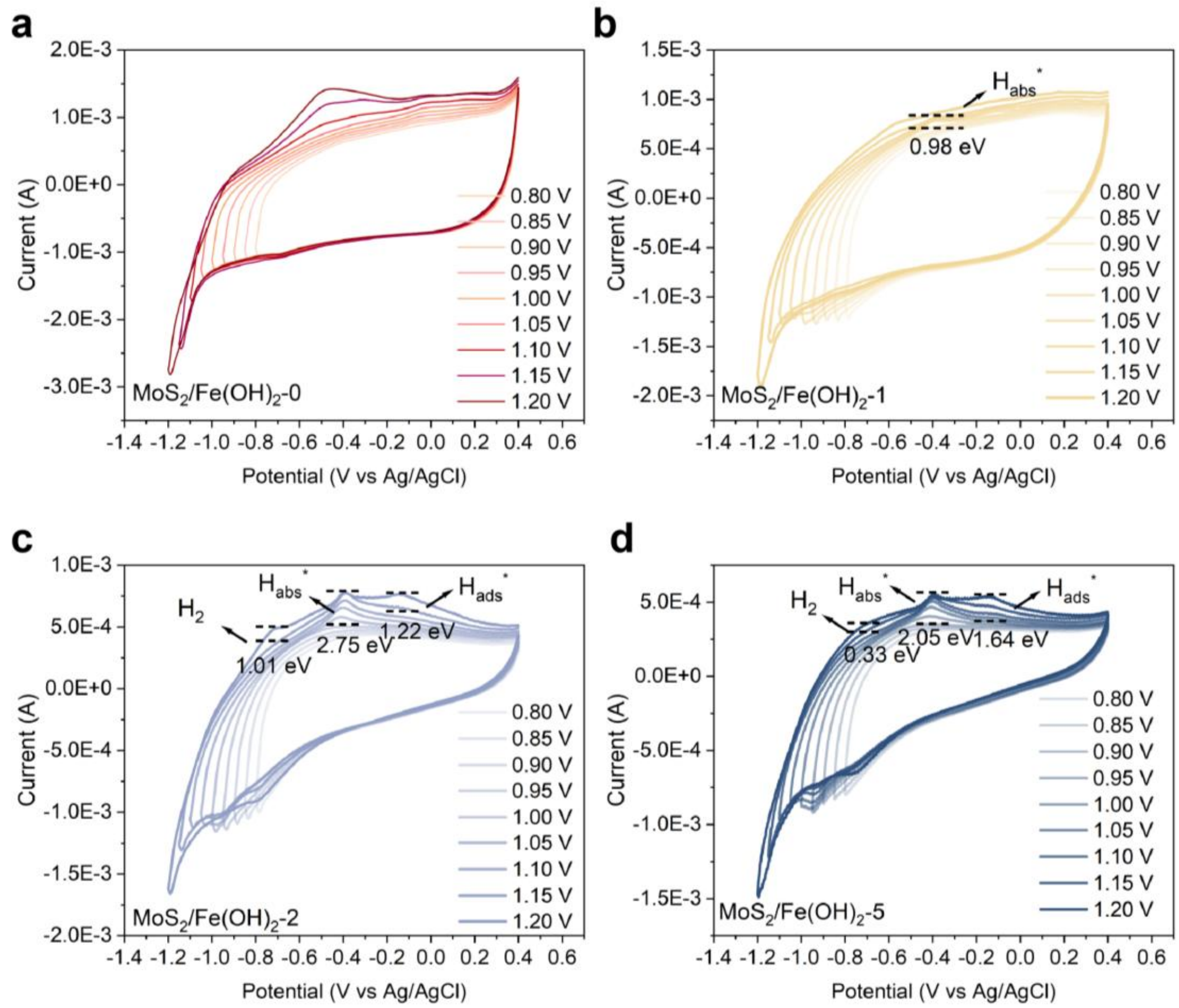

Figure S16. Cyclic voltammetry curves of $MoS_2/Fe(OH)_2$-z. z = (a) 0, (b) 1, (c) 2 and (d) 5.

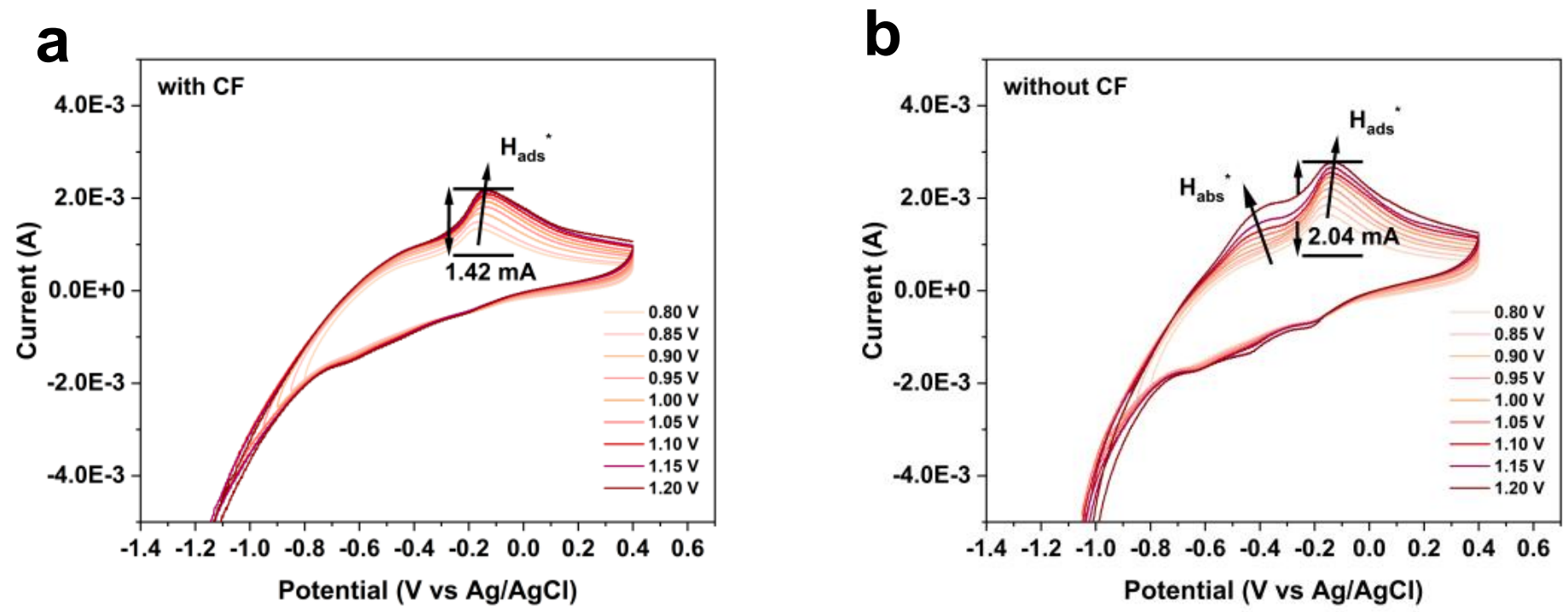

Figure S17. Cyclic voltammetry curves of $MoS_2/Fe(OH)_2$. (a) with or (b) without chloroform.

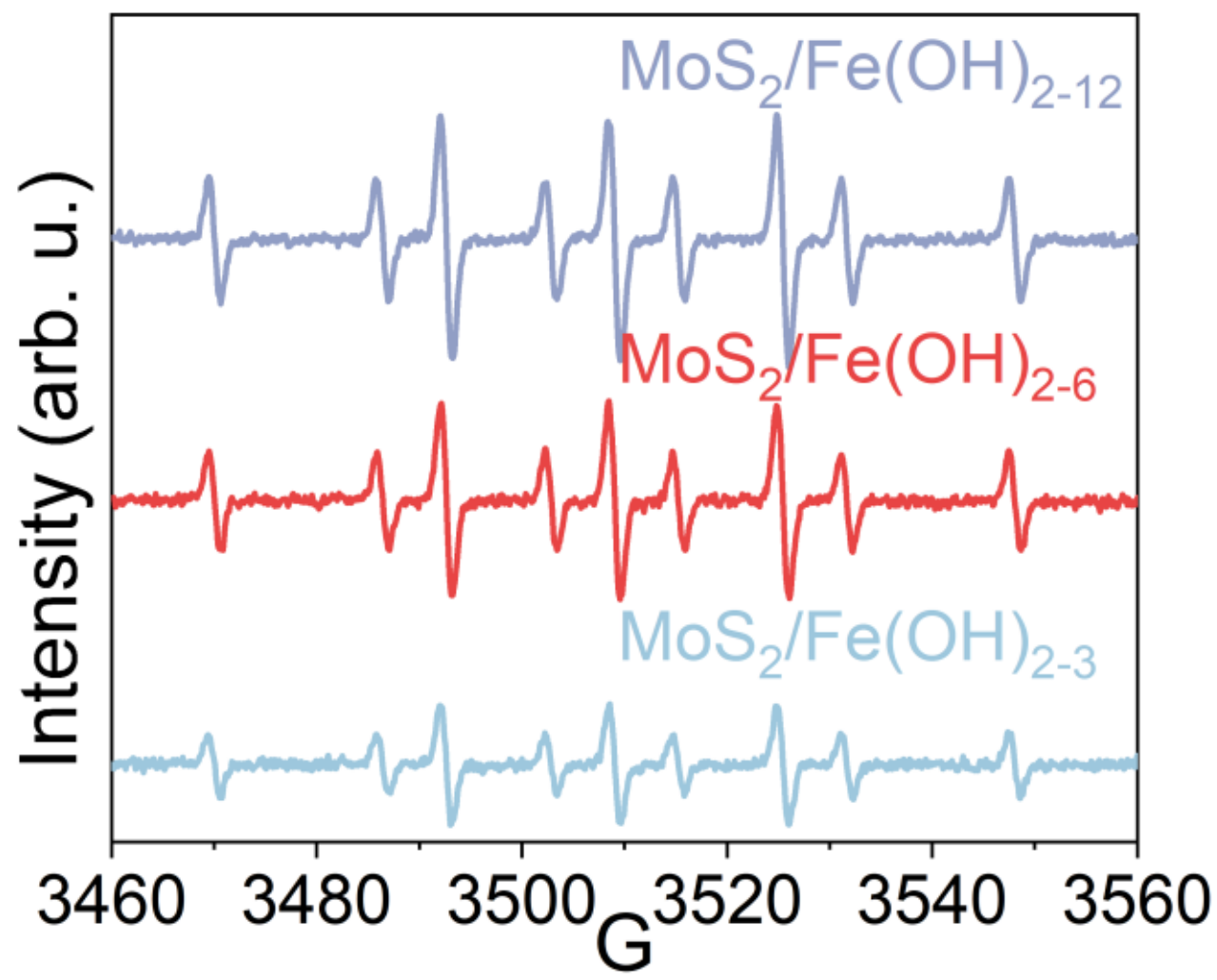

Figure S18. Electron paramagnetic resonance (EPR) spectra using DMPO. The EPR signals identify Fe(II) as the generator of H* under the reaction conditions.

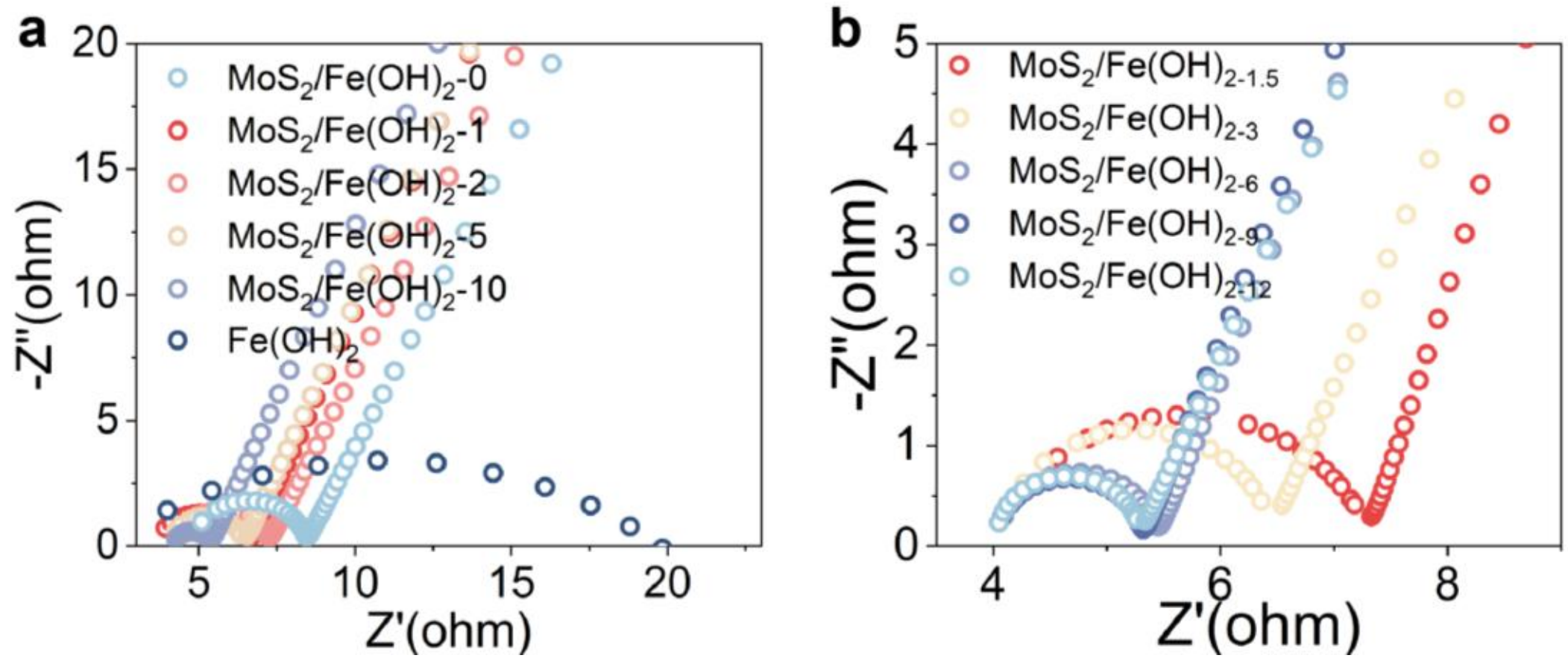

Figure S19. Electrochemical impedance spectroscopy (EIS) spectra. (a) $MoS_2/Fe(OH)_2$-z and (b) $MoS_2/Fe(OH)_{2-y}$

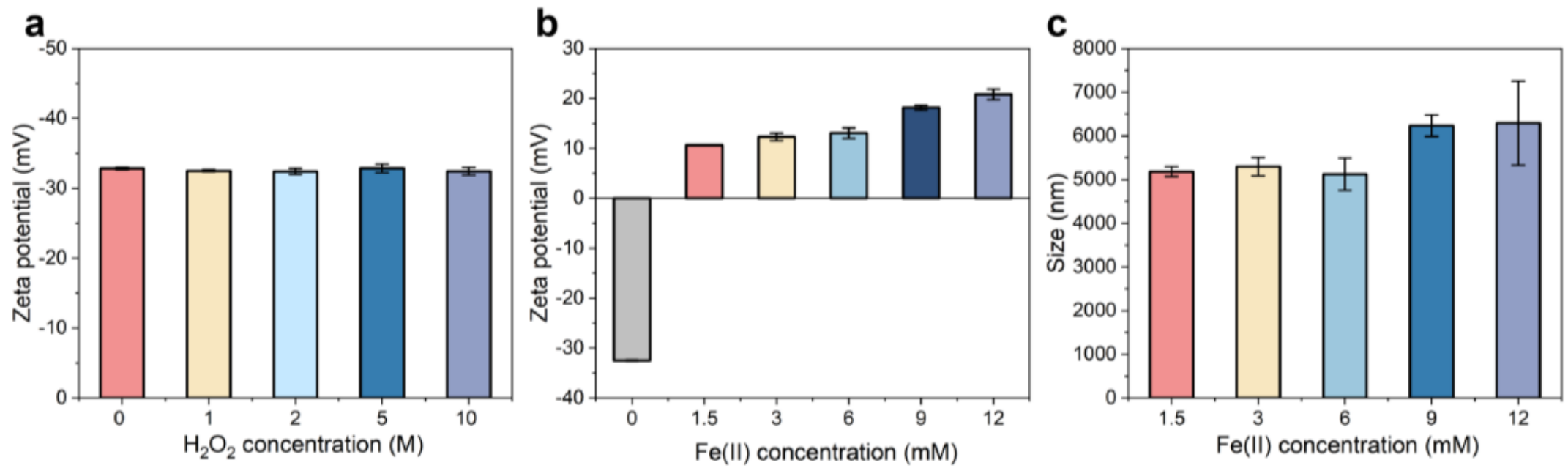

Figure S20. Zeta potential and particle size of $MoS_2/Fe(OH)_2$. (a) zeta potential of $MoS_2/Fe(OH)_2$-z, (b) zeta potential of $MoS_2/Fe(OH)_{2\text{-}y}$, (c) particle size of $MoS_2/Fe(OH)_{2\text{-}y}$. Data are presented as mean ± standard error (SE) from n = 2 independent experiments. Source data are provided in Source Data 2.

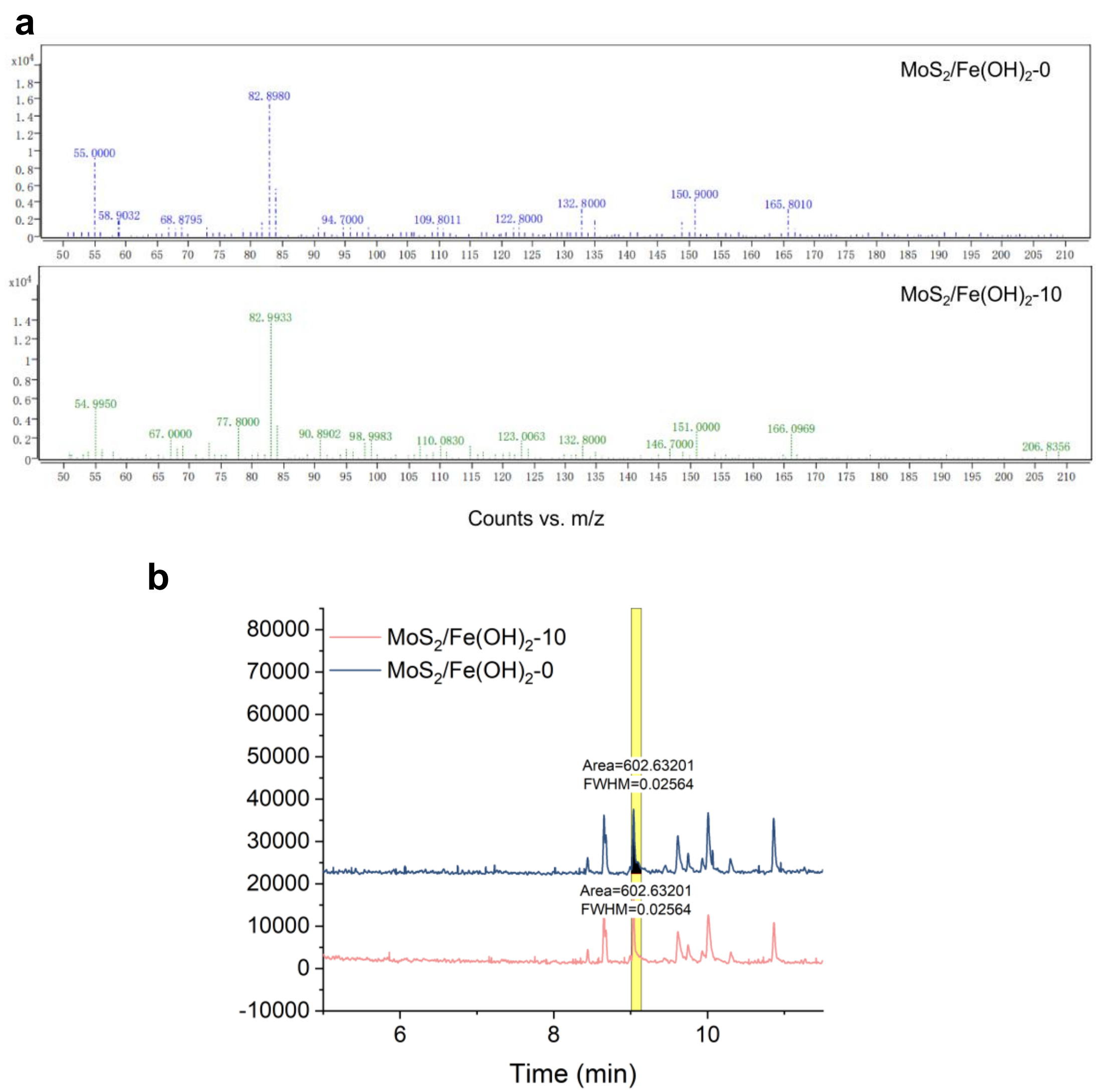

Figure S21. Mass spectrometric identification of the $\cdot CCl_3$ radical adduct product $TCCl_3$. (a) Mass spectrum of $\cdot CCl_3$ radical adduct product $TCCl_3$ and (b) EIC peak area of $TCCl_3$ (retention time = 9.37 min).

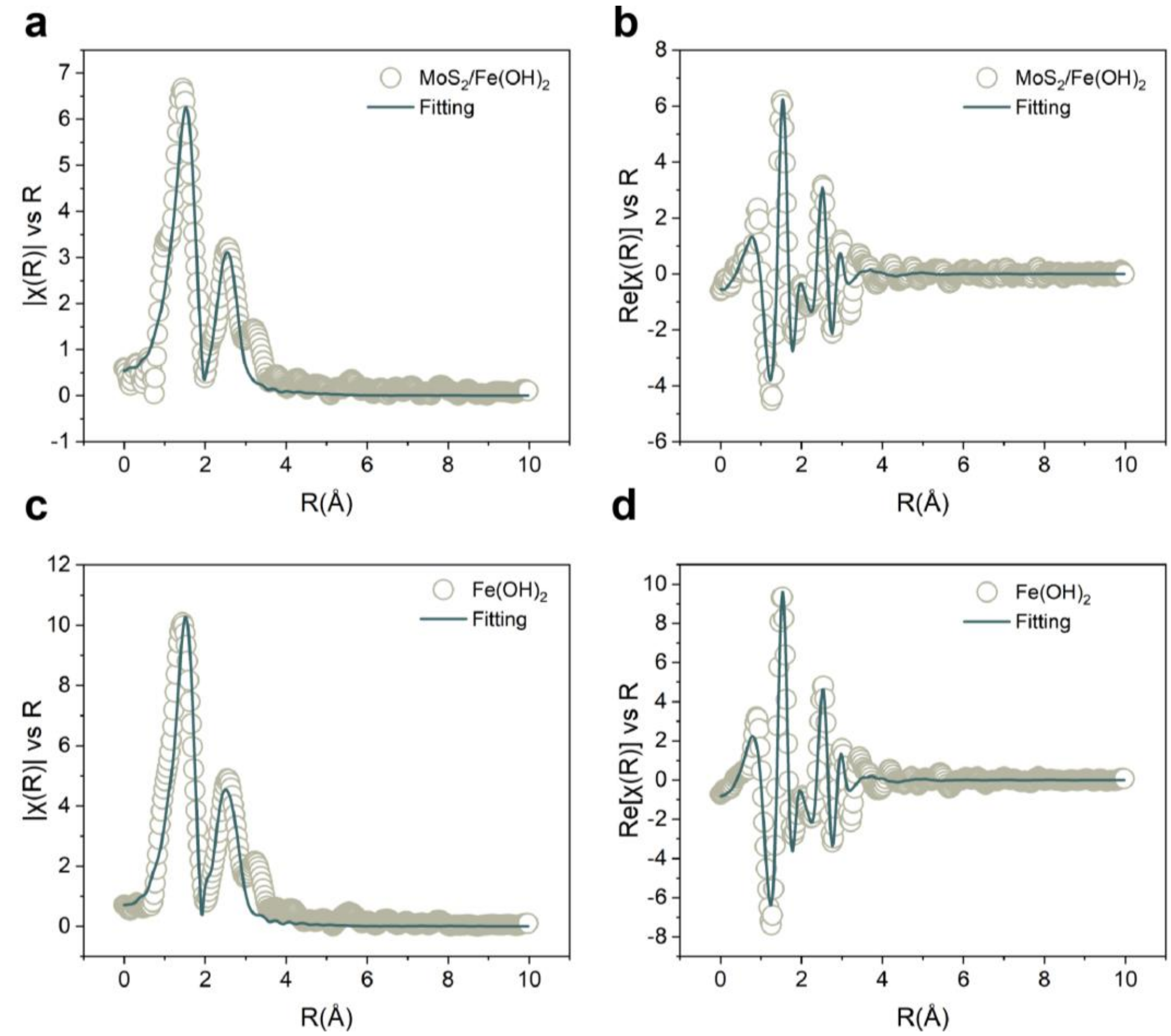

Figure S22. R-space extended X-ray absorption fine structure (EXAFS) fitting results of $MoS_2/Fe(OH)_2$ and $Fe(OH)_2$. (a) |χ(R)| for $MoS_2/Fe(OH)_2$, (b) Corresponding real part, Reχ(R), for $MoS_2/Fe(OH)_2$, (c) |χ(R)| for $Fe(OH)_2$ and (d) Corresponding real part, Reχ(R), for $Fe(OH)_2$.

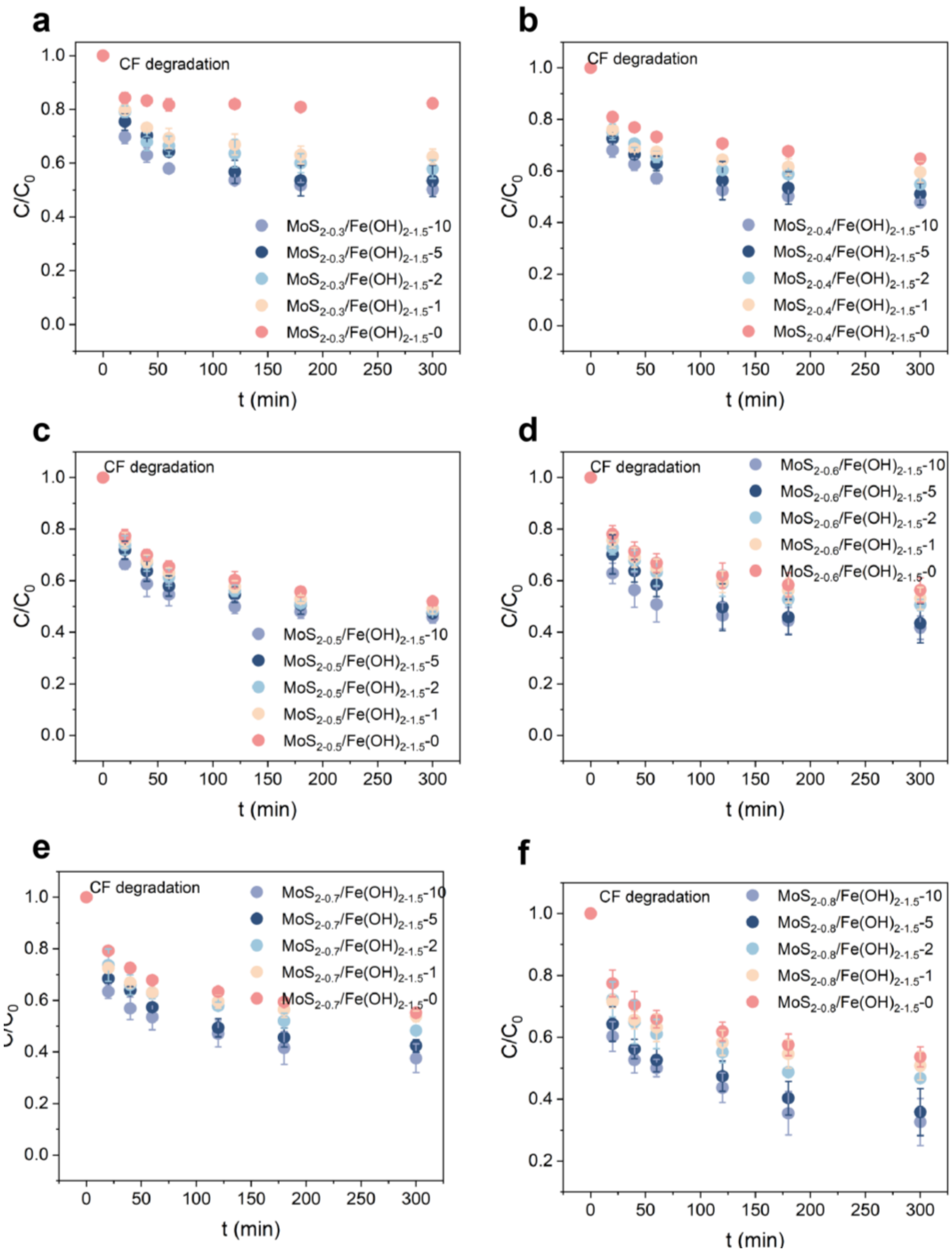

Figure S23. Chloroform degradation by $MoS_2/Fe(OH)_{2\text{-}1.5}$ (initial concentration of chloroform = 20 µM) (a) $MoS_{2\text{-}0.3}/Fe(OH)_{2\text{-}1.5}$, (b) $MoS_{2\text{-}0.4}/Fe(OH)_{2\text{-}1.5}$, (c) $MoS_{2\text{-}0.5}/Fe(OH)_{2\text{-}1.5}$, (d) $MoS_{2\text{-}0.6}/Fe(OH)_{2\text{-}1.5}$, (e) $MoS_{2\text{-}0.7}/Fe(OH)_{2\text{-}1.5}$, (f) $MoS_{2\text{-}0.8}/Fe(OH)_{2\text{-}1.5}$. Data are presented as mean ± standard error (SE) from n = 2 independent experiments. Source data are provided in Source Data 2.

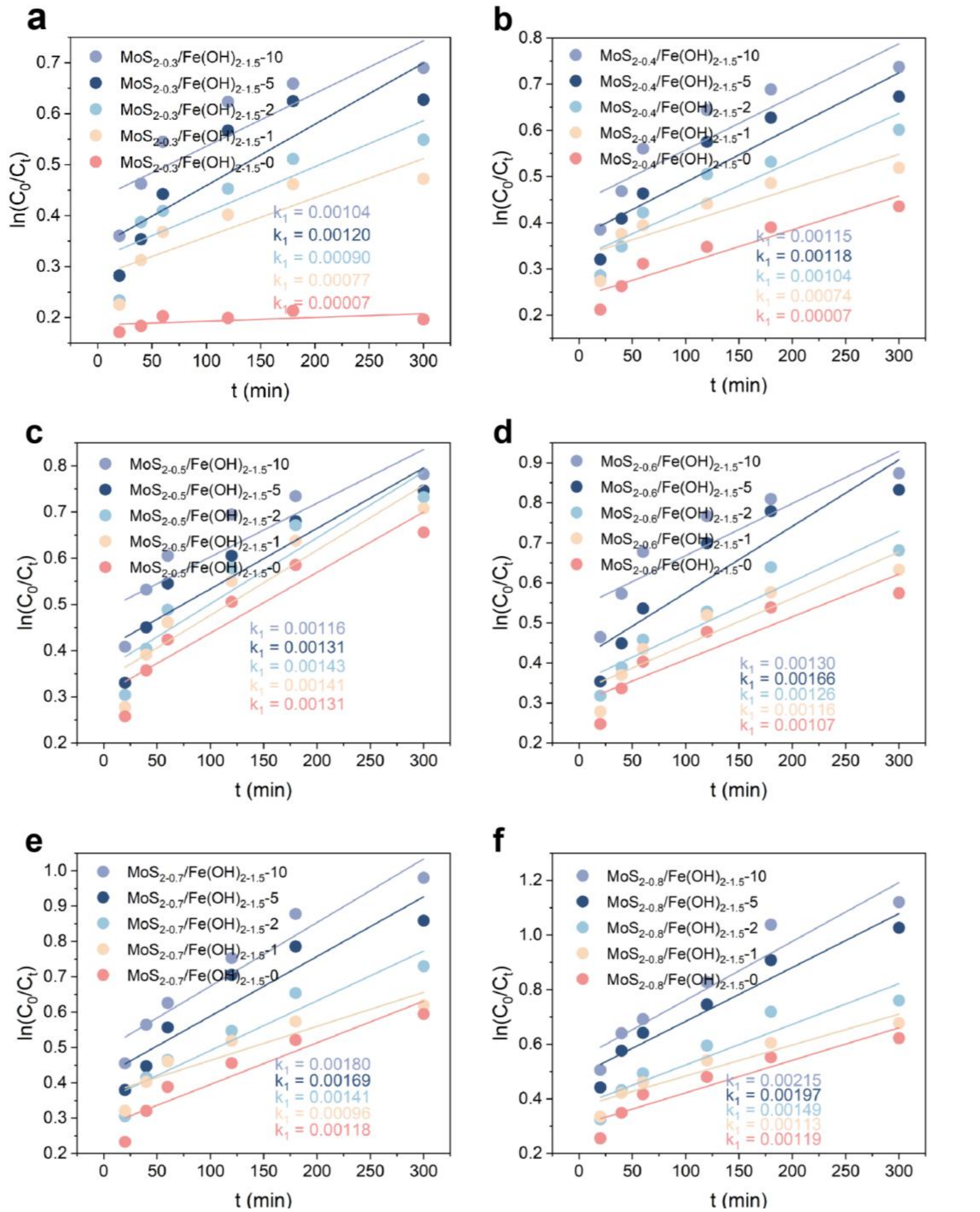

Figure S24. Pseudo-first-order kinetics fitting for chloroform degradation by $MoS_2/Fe(OH)_{2\text{-}1.5}$. (a) $MoS_{2\text{-}0.3}/Fe(OH)_{2\text{-}1.5}$, (b) $MoS_{2\text{-}0.4}/Fe(OH)_{2\text{-}1.5}$, (c) $MoS_{2\text{-}0.5}/Fe(OH)_{2\text{-}1.5}$, (d) $MoS_{2\text{-}0.6}/Fe(OH)_{2\text{-}1.5}$, (e) $MoS_{2\text{-}0.7}/Fe(OH)_{2\text{-}1.5}$, (f) $MoS_{2\text{-}0.8}/Fe(OH)_{2\text{-}1.5}$. Data are presented as mean ± standard error (SE) from n = 2 independent experiments. Source data are provided in Source Data 2.

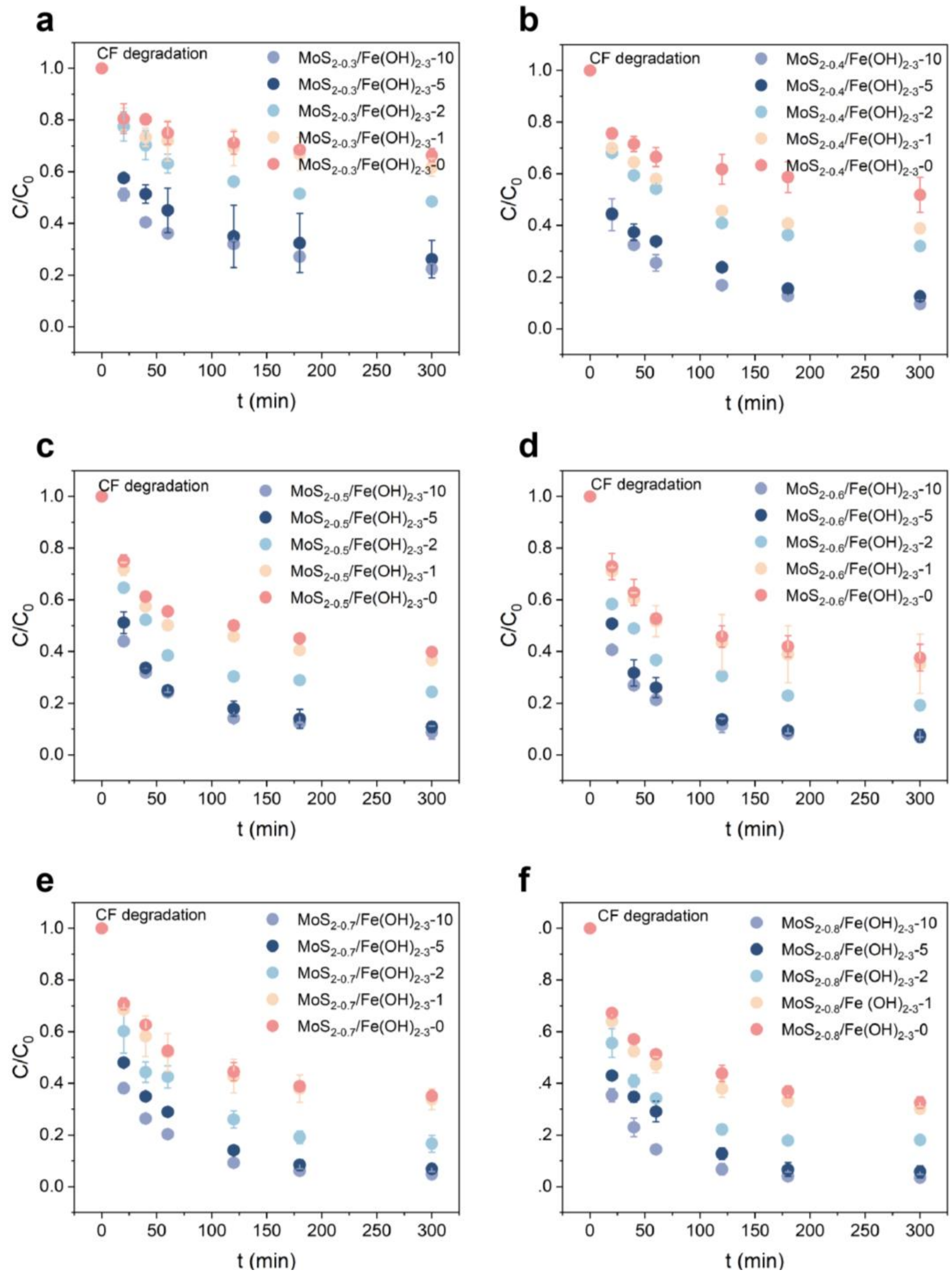

Figure S25. Chloroform degradation by $MoS_2/Fe(OH)_{2-3}$ (initial concentration of chloroform = 20 µM). (a) $MoS_{2-0.3}/Fe(OH)_{2-3}$, (b) $MoS_{2-0.4}/Fe(OH)_{2-3}$, (c) $MoS_{2-0.5}/Fe(OH)_{2-3}$, (d) $MoS_{2-0.6}/Fe(OH)_{2-3}$, (e) $MoS_{2-0.7}/Fe(OH)_{2-3}$, (f) $MoS_{2-0.8}/Fe(OH)_{2-3}$. Data are presented as mean ± standard error (SE) from n = 2 independent experiments. Source data are provided in Source Data 2.

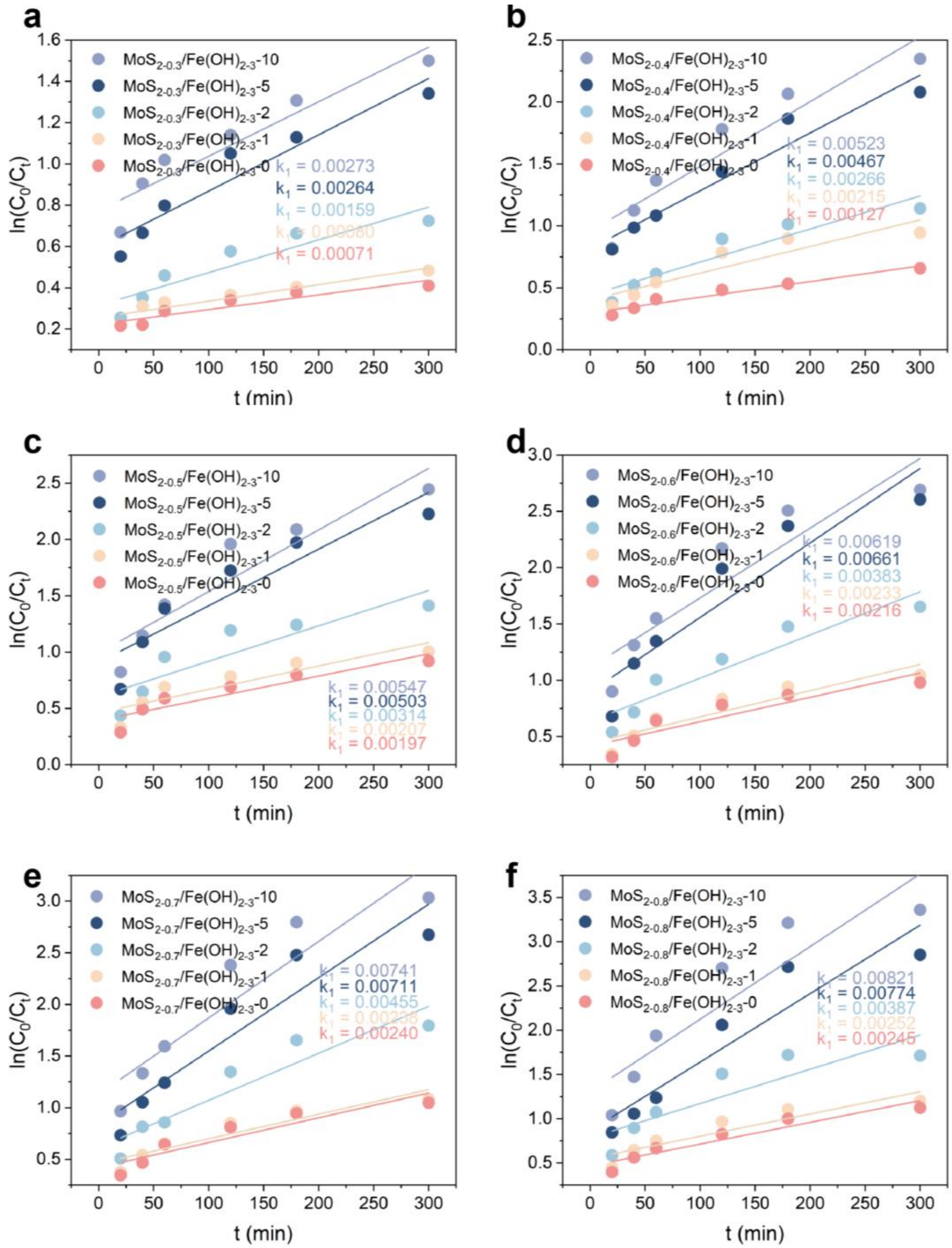

Figure S26. Pseudo-first-order kinetics fitting for chloroform degradation by $MoS_2/Fe(OH)_{2-3}$. (a) $MoS_{2-0.3}/Fe(OH)_{2-3}$, (b) $MoS_{2-0.4}/Fe(OH)_{2-3}$, (c) $MoS_{2-0.5}/Fe(OH)_{2-3}$, (d) $MoS_{2-0.6}/Fe(OH)_{2-3}$, (e) $MoS_{2-0.7}/Fe(OH)_{2-3}$, (f) $MoS_{2-0.8}/Fe(OH)_{2-3}$. Data are presented as mean ± standard error (SE) from n = 2 independent experiments. Source data are provided in Source Data 2.

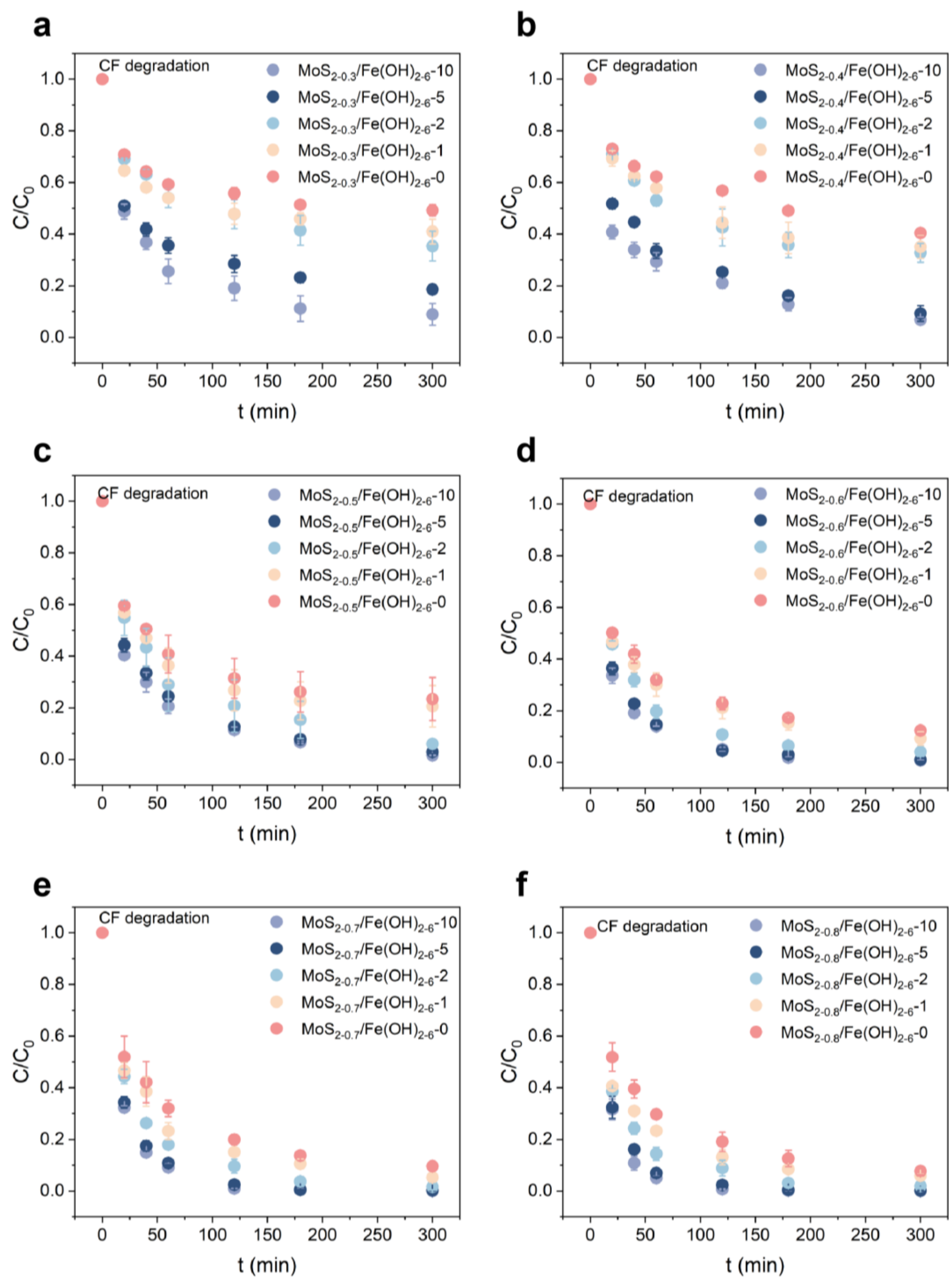

Figure S27. Chloroform degradation by $MoS_2/Fe(OH)_{2\text{-}6}$ (initial concentration of chloroform = 20 μM). (a) $MoS_{2\text{-}0.3}/Fe(OH)_{2\text{-}6}$, (b) $MoS_{2\text{-}0.4}/Fe(OH)_{2\text{-}6}$, (c) $MoS_{2\text{-}0.5}/Fe(OH)_{2\text{-}6}$, (d) $MoS_{2\text{-}0.6}/Fe(OH)_{2\text{-}6}$, (e) $MoS_{2\text{-}0.7}/Fe(OH)_{2\text{-}6}$, (f) $MoS_{2\text{-}0.8}/Fe(OH)_{2\text{-}6}$. Data are presented as mean ± standard error (SE) from n = 2 independent experiments. Source data are provided in Source Data 2.

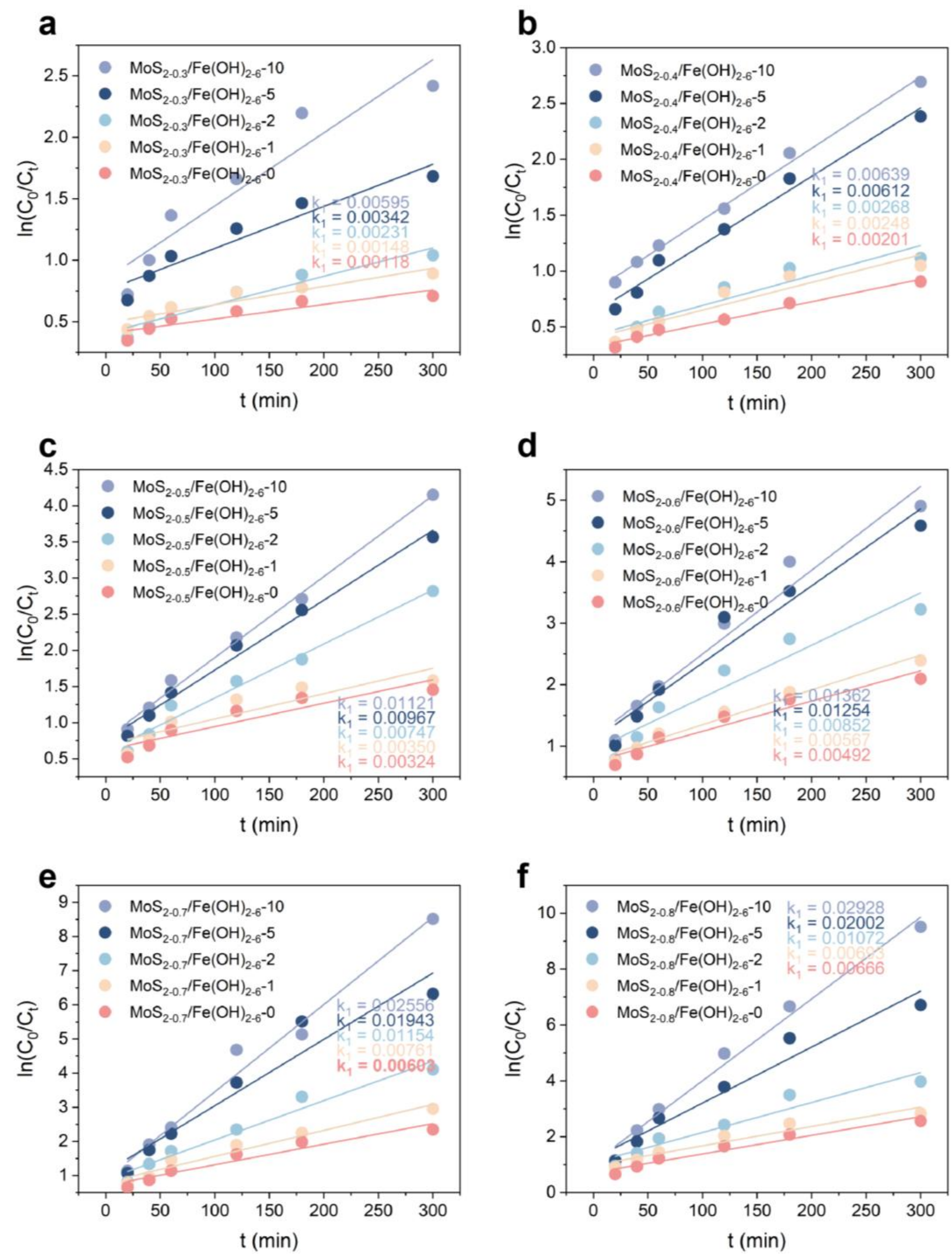

Figure S28. Pseudo-first-order kinetics fitting for chloroform degradation by $MoS_2/Fe(OH)_{2\text{-}6}$. (a) $MoS_{2\text{-}0.3}/Fe(OH)_{2\text{-}6}$, (b) $MoS_{2\text{-}0.4}/Fe(OH)_{2\text{-}6}$, (c) $MoS_{2\text{-}0.5}/Fe(OH)_{2\text{-}6}$, (d) $MoS_{2\text{-}0.6}/Fe(OH)_{2\text{-}6}$, (e) $MoS_{2\text{-}0.7}/Fe(OH)_{2\text{-}6}$, (f) $MoS_{2\text{-}0.8}/Fe(OH)_{2\text{-}6}$. Data are presented as mean ± standard error (SE) from n = 2 independent experiments. Source data are provided in Source Data 2.

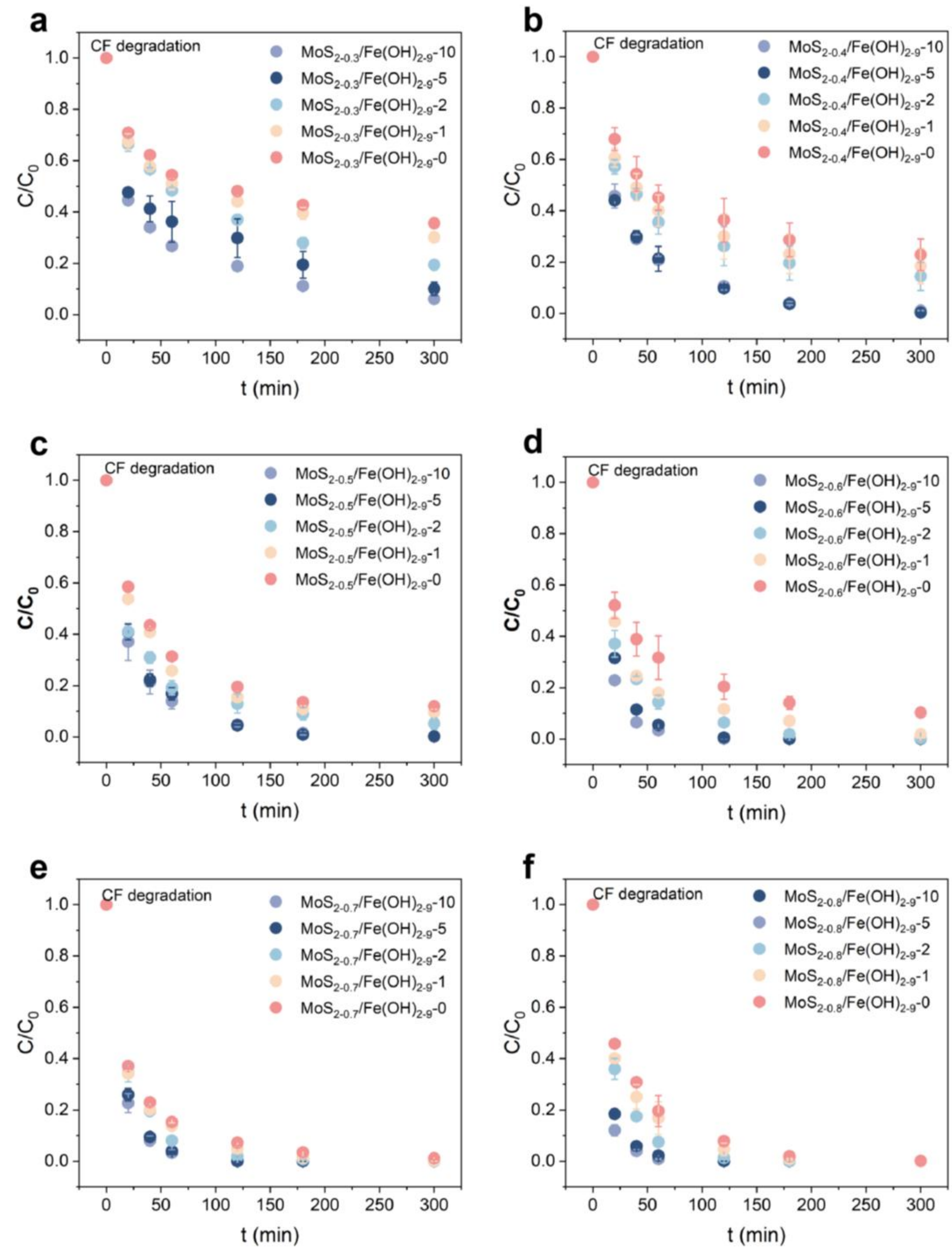

Figure S29. Chloroform degradation by $MoS_2/Fe(OH)_{2\text{-}9}$ (initial concentration of chloroform = 20 μM). (a) $MoS_{2\text{-}0.3}/Fe(OH)_{2\text{-}9}$, (b) $MoS_{2\text{-}0.4}/Fe(OH)_{2\text{-}9}$, (c) $MoS_{2\text{-}0.5}/Fe(OH)_{2\text{-}9}$, (d) $MoS_{2\text{-}0.6}/Fe(OH)_{2\text{-}9}$, (e) $MoS_{2\text{-}0.7}/Fe(OH)_{2\text{-}9}$, (f) $MoS_{2\text{-}0.8}/Fe(OH)_{2\text{-}9}$. Data are presented as mean ± standard error (SE) from n = 2 independent experiments. Source data are provided in Source Data 2.

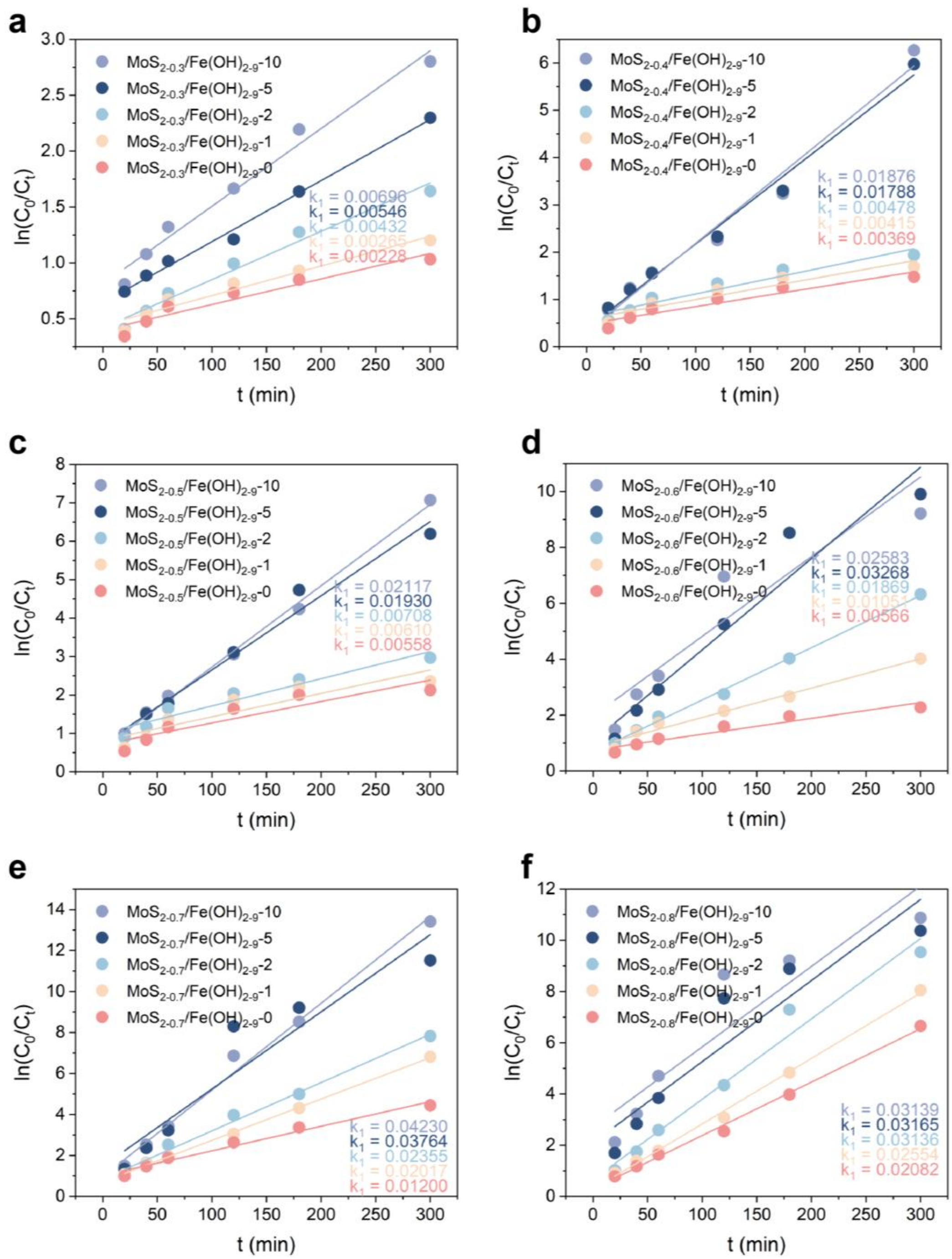

Figure S30. Pseudo-first-order kinetics fitting for chloroform degradation by $MoS_2/Fe(OH)_{2\text{-}9}$. (a) $MoS_{2\text{-}0.3}/Fe(OH)_{2\text{-}9}$, (b) $MoS_{2\text{-}0.4}/Fe(OH)_{2\text{-}9}$, (c) $MoS_{2\text{-}0.5}/Fe(OH)_{2\text{-}9}$, (d) $MoS_{2\text{-}0.6}/Fe(OH)_{2\text{-}9}$, (e) $MoS_{2\text{-}0.7}/Fe(OH)_{2\text{-}9}$, (f) $MoS_{2\text{-}0.8}/Fe(OH)_{2\text{-}9}$ Data are presented as mean ± standard error (SE) from n = 2 independent experiments. Source data are provided in Source Data 2.

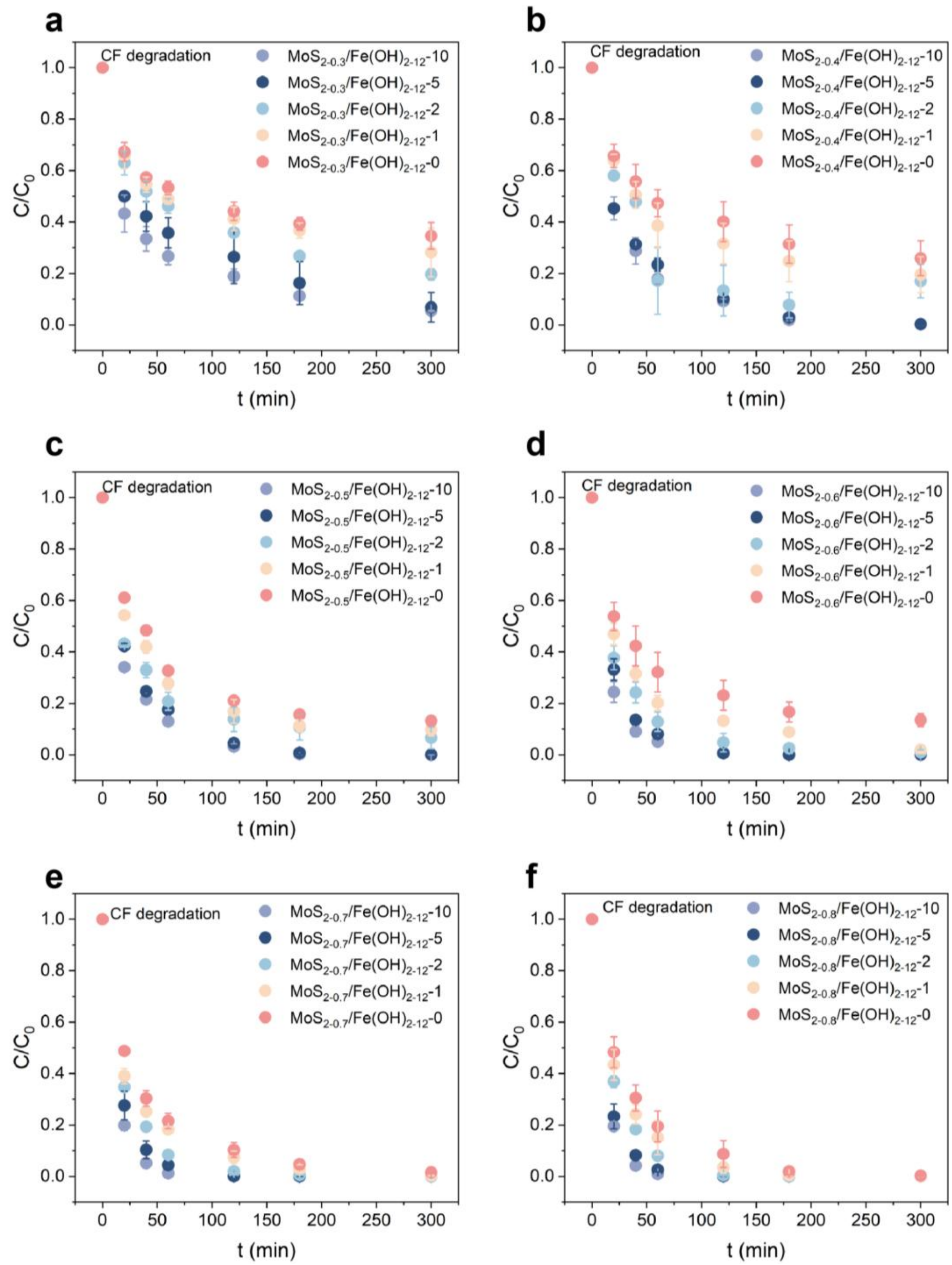

Figure S31. Chloroform degradation by $MoS_2/Fe(OH)_{2\text{-}12}$ (initial concentration of chloroform = 20 μM). (a) $MoS_{2\text{-}0.3}/Fe(OH)_{2\text{-}12}$, (b) $MoS_{2\text{-}0.4}/Fe(OH)_{2\text{-}12}$, (c) $MoS_{2\text{-}0.5}/Fe(OH)_{2\text{-}12}$, (d) $MoS_{2\text{-}0.6}/Fe(OH)_{2\text{-}12}$, (e) $MoS_{2\text{-}0.7}/Fe(OH)_{2\text{-}12}$, (f) $MoS_{2\text{-}0.8}/Fe(OH)_{2\text{-}12}$. Data are presented as mean ± standard error (SE) from n = 2 independent experiments. Source data are provided in Source Data 2.

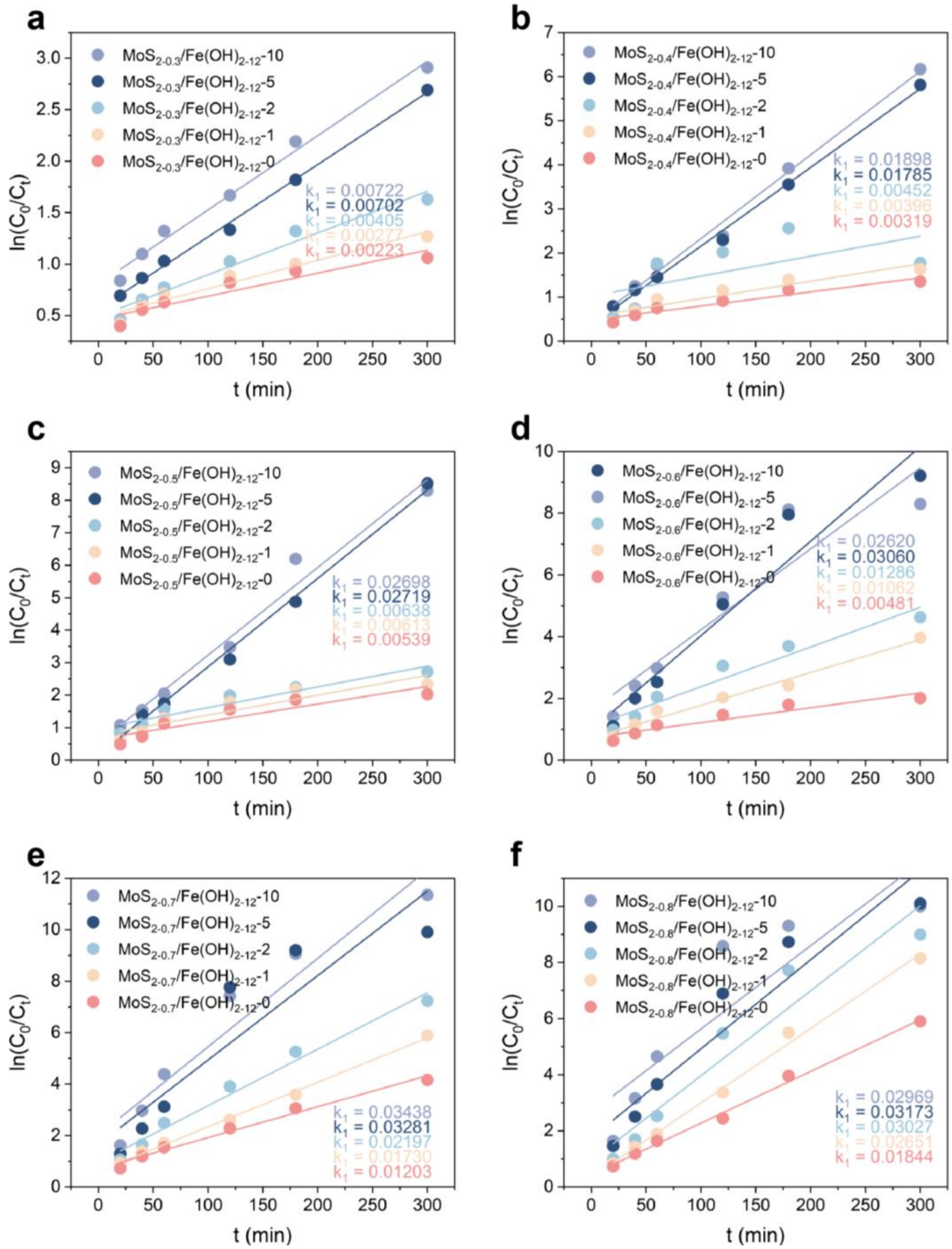

Figure S32. Pseudo-first-order kinetics fitting for chloroform degradation by $MoS_2/Fe(OH)_{2\text{-}12}$. (a) $MoS_{2\text{-}0.3}/Fe(OH)_{2\text{-}12}$, (b) $MoS_{2\text{-}0.4}/Fe(OH)_{2\text{-}12}$, (c) $MoS_{2\text{-}0.5}/Fe(OH)_{2\text{-}12}$, (d) $MoS_{2\text{-}0.6}/Fe(OH)_{2\text{-}12}$, (e) $MoS_{2\text{-}0.7}/Fe(OH)_{2\text{-}12}$, (f) $MoS_{2\text{-}0.8}/Fe(OH)_{2\text{-}12}$. Data are presented as mean ± standard error (SE) from n = 2 independent experiments. Source data are provided in Source Data 2.

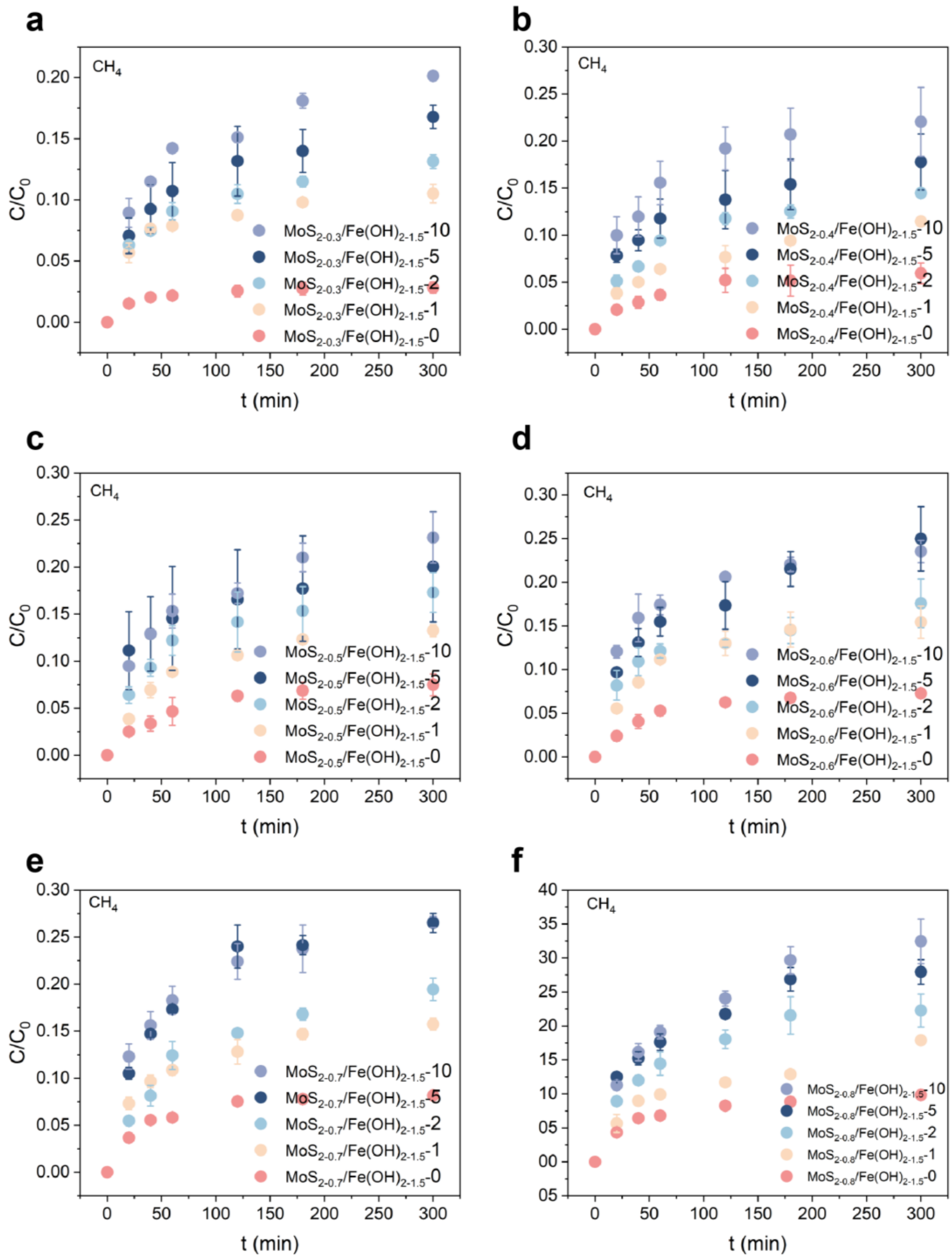

Figure S33. Methane generation during chloroform degradation by $MoS_2/Fe(OH)_{2\text{-}1.5}$. (a) $MoS_{2\text{-}0.3}/Fe(OH)_{2\text{-}1.5}$, (b) $MoS_{2\text{-}0.4}/Fe(OH)_{2\text{-}1.5}$, (c) $MoS_{2\text{-}0.5}/Fe(OH)_{2\text{-}1.5}$, (d) $MoS_{2\text{-}0.6}/Fe(OH)_{2\text{-}1.5}$, (e) $MoS_{2\text{-}0.7}/Fe(OH)_{2\text{-}1.5}$, (f) $MoS_{2\text{-}0.8}/Fe(OH)_{2\text{-}1.5}$. Data are presented as mean ± standard error (SE) from n = 2 independent experiments. Source data are provided in Source Data 2.

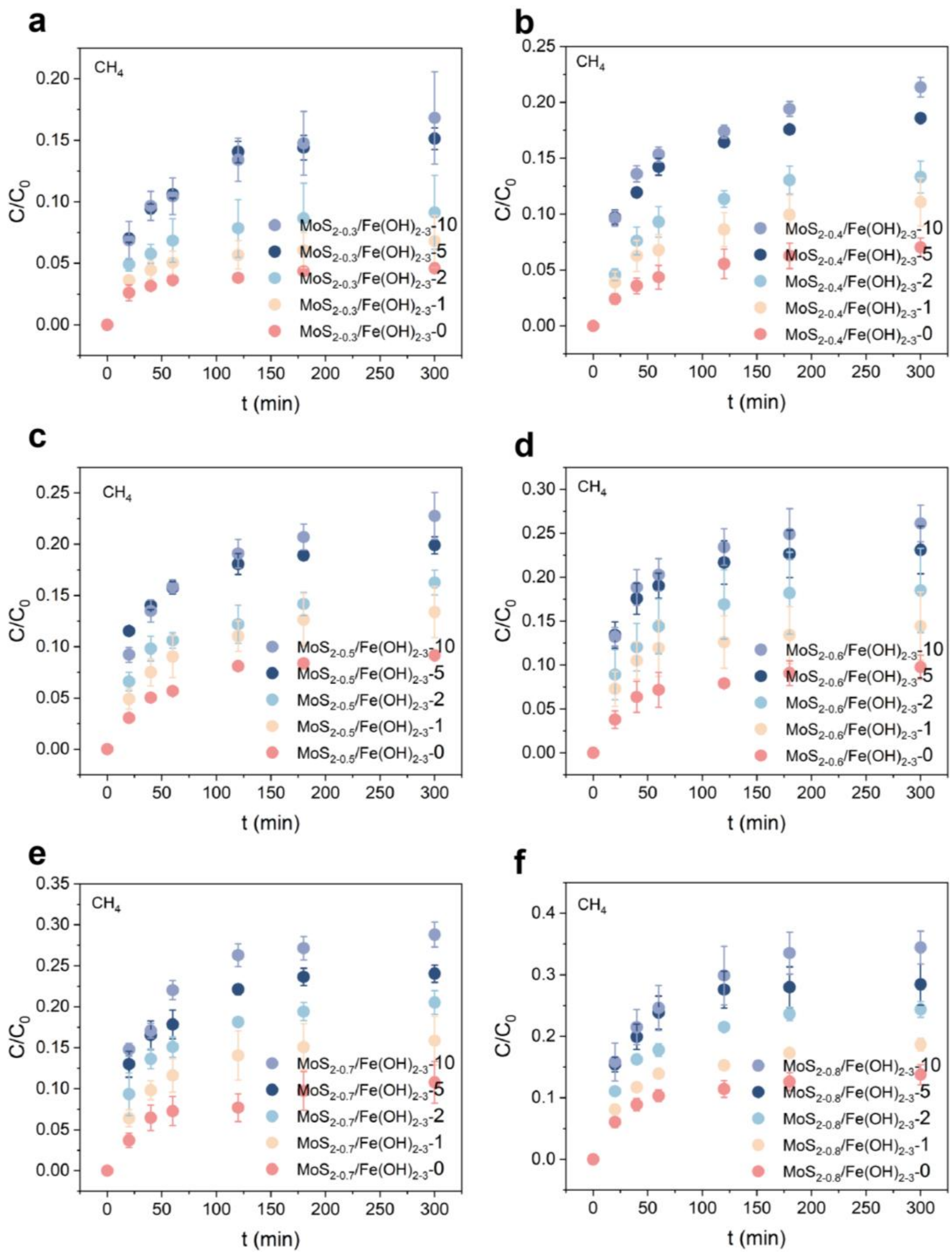

Figure S34. Methane generation during chloroform degradation by $MoS_2/Fe(OH)_{2\text{-}3}$. (a) $MoS_{2\text{-}0.3}/Fe(OH)_{2\text{-}3}$, (b) $MoS_{2\text{-}0.4}/Fe(OH)_{2\text{-}3}$, (c) $MoS_{2\text{-}0.5}/Fe(OH)_{2\text{-}3}$, (d) $MoS_{2\text{-}0.6}/Fe(OH)_{2\text{-}3}$, (e) $MoS_{2\text{-}0.7}/Fe(OH)_{2\text{-}3}$, (f) $MoS_{2\text{-}0.8}/Fe(OH)_{2\text{-}3}$. Data are presented as mean ± standard error (SE) from n = 2 independent experiments. Source data are provided in Source Data 2.

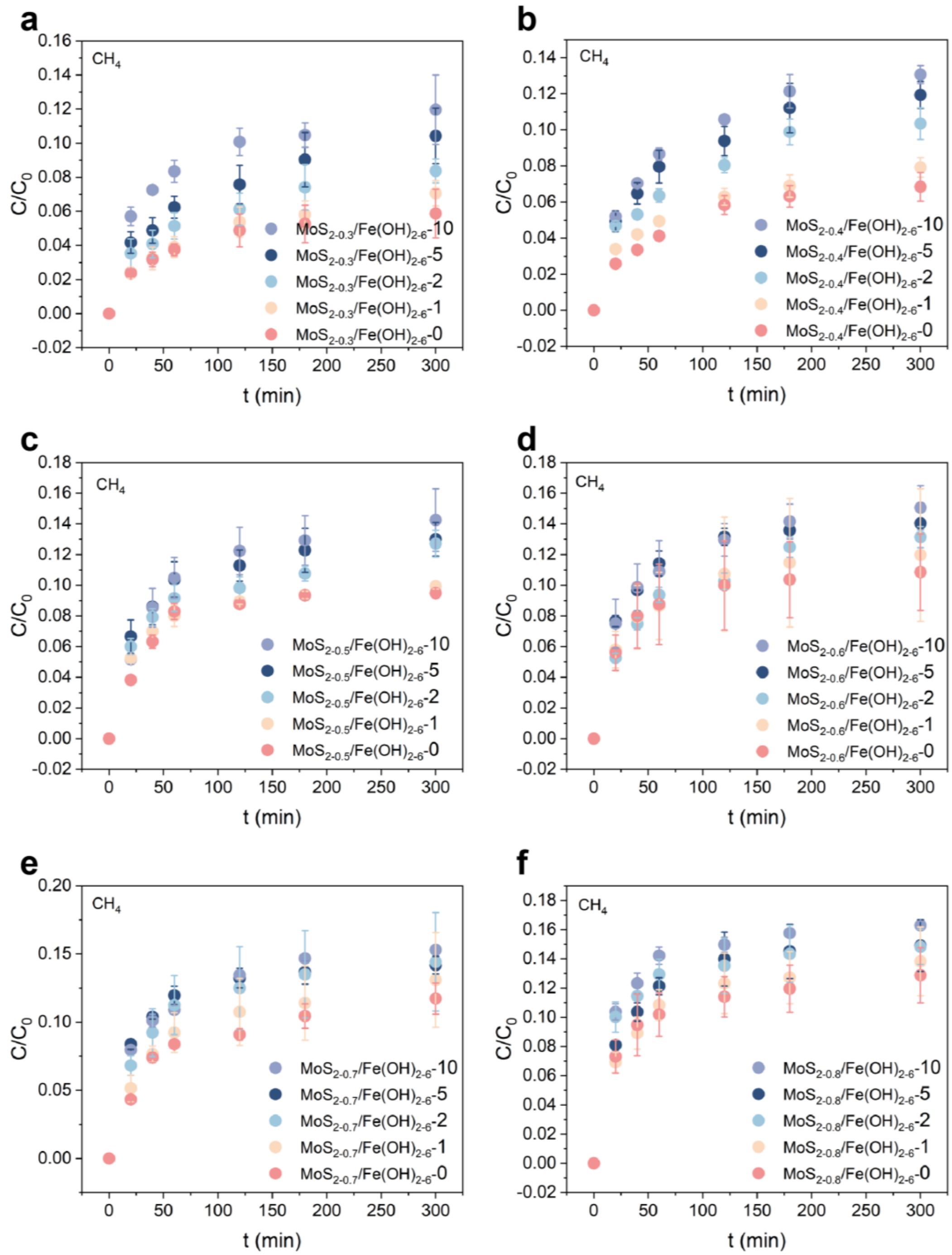

Figure S35. Methane generation during chloroform degradation by MoS_2/$Fe(OH)_{2\text{-}6}$. (a) $MoS_{2\text{-}0.3}$/$Fe(OH)_{2\text{-}6}$, (b) $MoS_{2\text{-}0.4}$/$Fe(OH)_{2\text{-}6}$, (c) $MoS_{2\text{-}0.5}$/$Fe(OH)_{2\text{-}6}$, (d) $MoS_{2\text{-}0.6}$/$Fe(OH)_{2\text{-}6}$, (e) $MoS_{2\text{-}0.7}$/$Fe(OH)_{2\text{-}6}$, (f) $MoS_{2\text{-}0.8}$/$Fe(OH)_{2\text{-}6}$. Data are presented as mean ± standard error (SE) from n = 2 independent experiments. Source data are provided in Source Data 2.

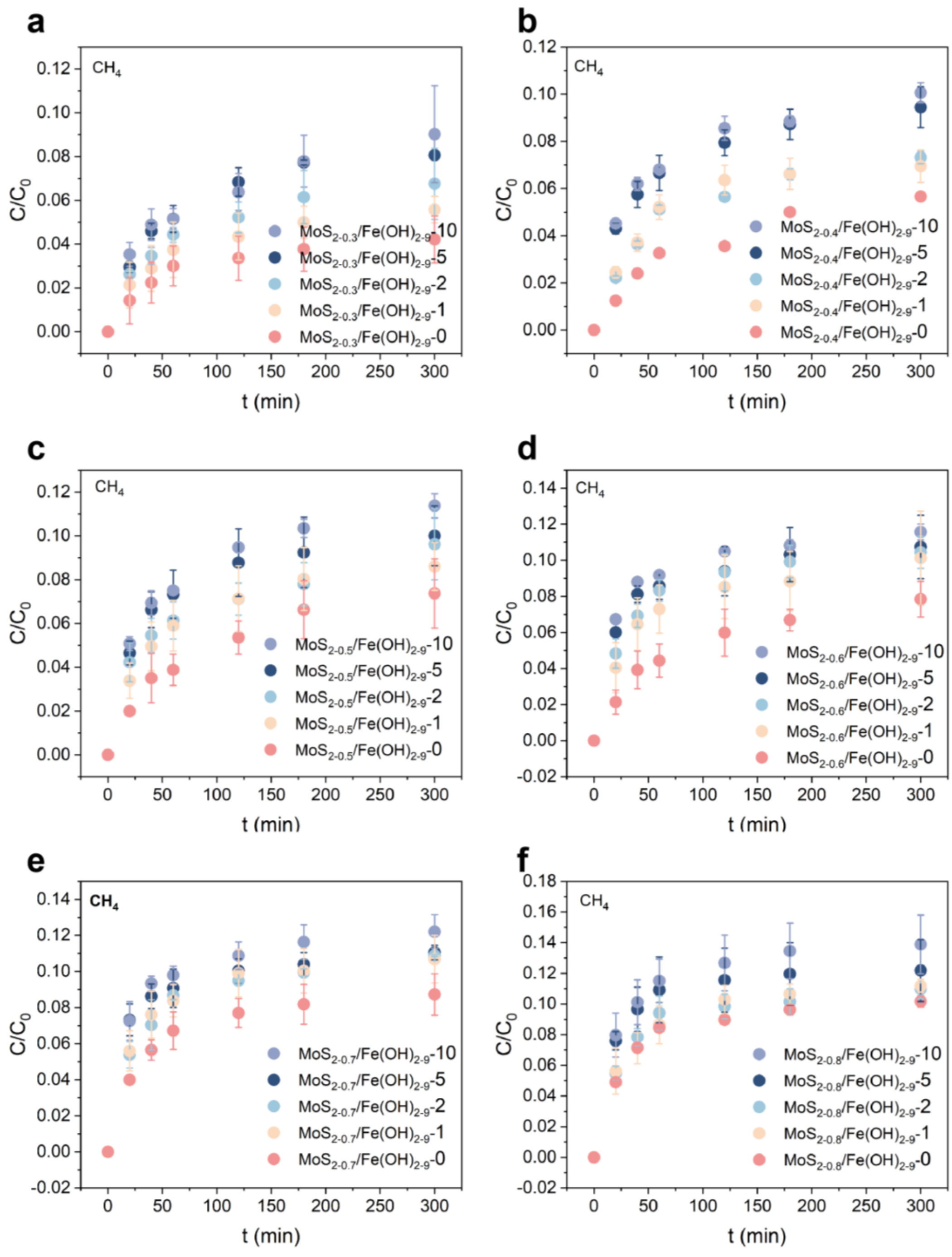

Figure S36. Methane generation during chloroform degradation by $MoS_2/Fe(OH)_{2\text{-}9}$. (a) $MoS_{2\text{-}0.3}/Fe(OH)_{2\text{-}9}$, (b) $MoS_{2\text{-}0.4}/Fe(OH)_{2\text{-}9}$, (c) $MoS_{2\text{-}0.5}/Fe(OH)_{2\text{-}9}$, (d) $MoS_{2\text{-}0.6}/Fe(OH)_{2\text{-}9}$, (e) $MoS_{2\text{-}0.7}/Fe(OH)_{2\text{-}9}$, (f) $MoS_{2\text{-}0.8}/Fe(OH)_{2\text{-}9}$. Data are presented as mean ± standard error (SE) from n = 2 independent experiments. Source data are provided in Source Data 2.

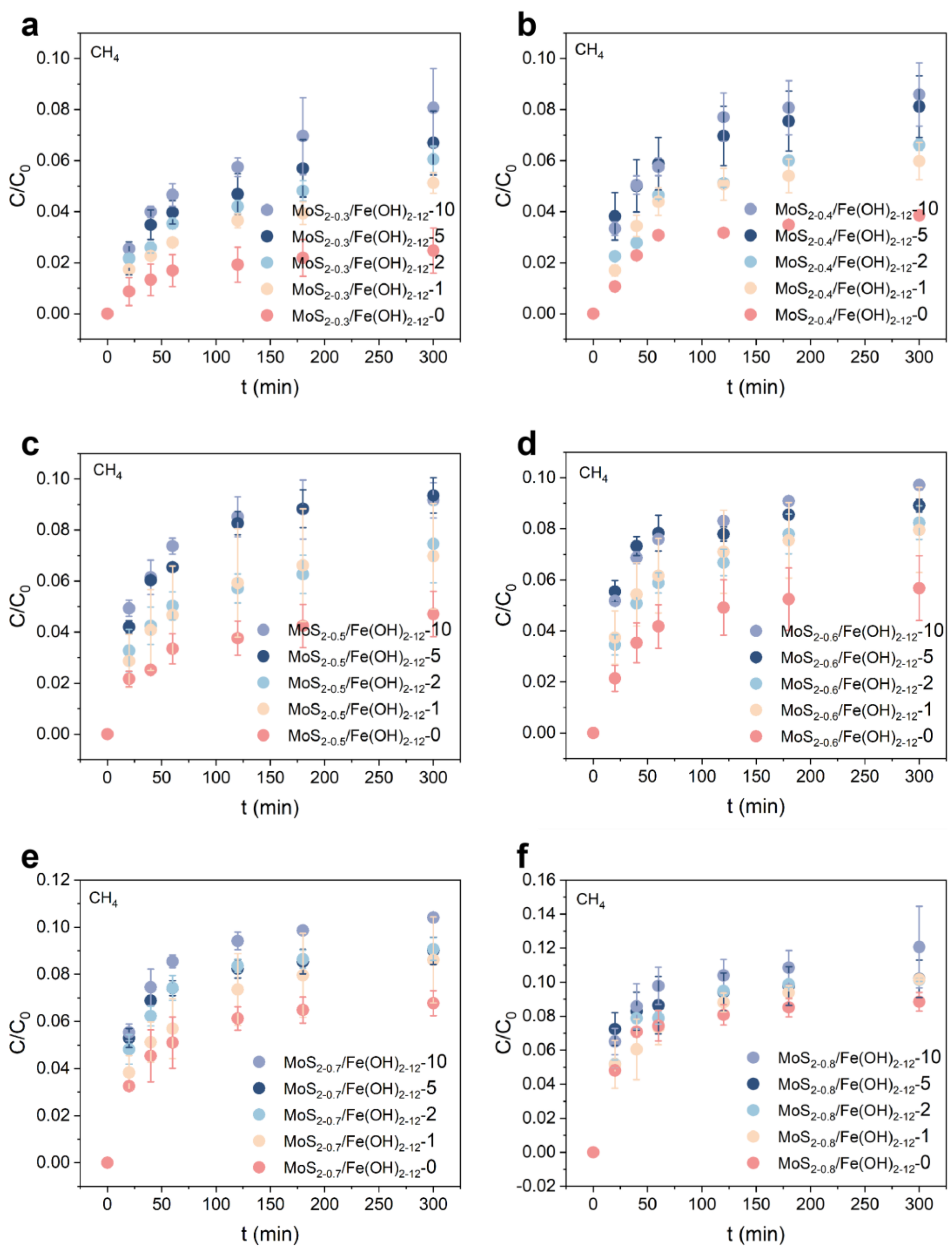

Figure S37. Methane generation during chloroform degradation by MoS_2/$Fe(OH)_{2-12}$. (a) $MoS_{2-0.3}$/$Fe(OH)_{2-12}$, (b) $MoS_{2-0.4}$/$Fe(OH)_{2-12}$, (c) $MoS_{2-0.5}$/$Fe(OH)_{2-12}$, (d) $MoS_{2-0.6}$/$Fe(OH)_{2-12}$, (e) $MoS_{2-0.7}$/$Fe(OH)_{2-12}$, (f) $MoS_{2-0.8}$/$Fe(OH)_{2-12}$. Data are presented as mean ± standard error (SE) from n = 2 independent experiments. Source data are provided in Source Data 2.

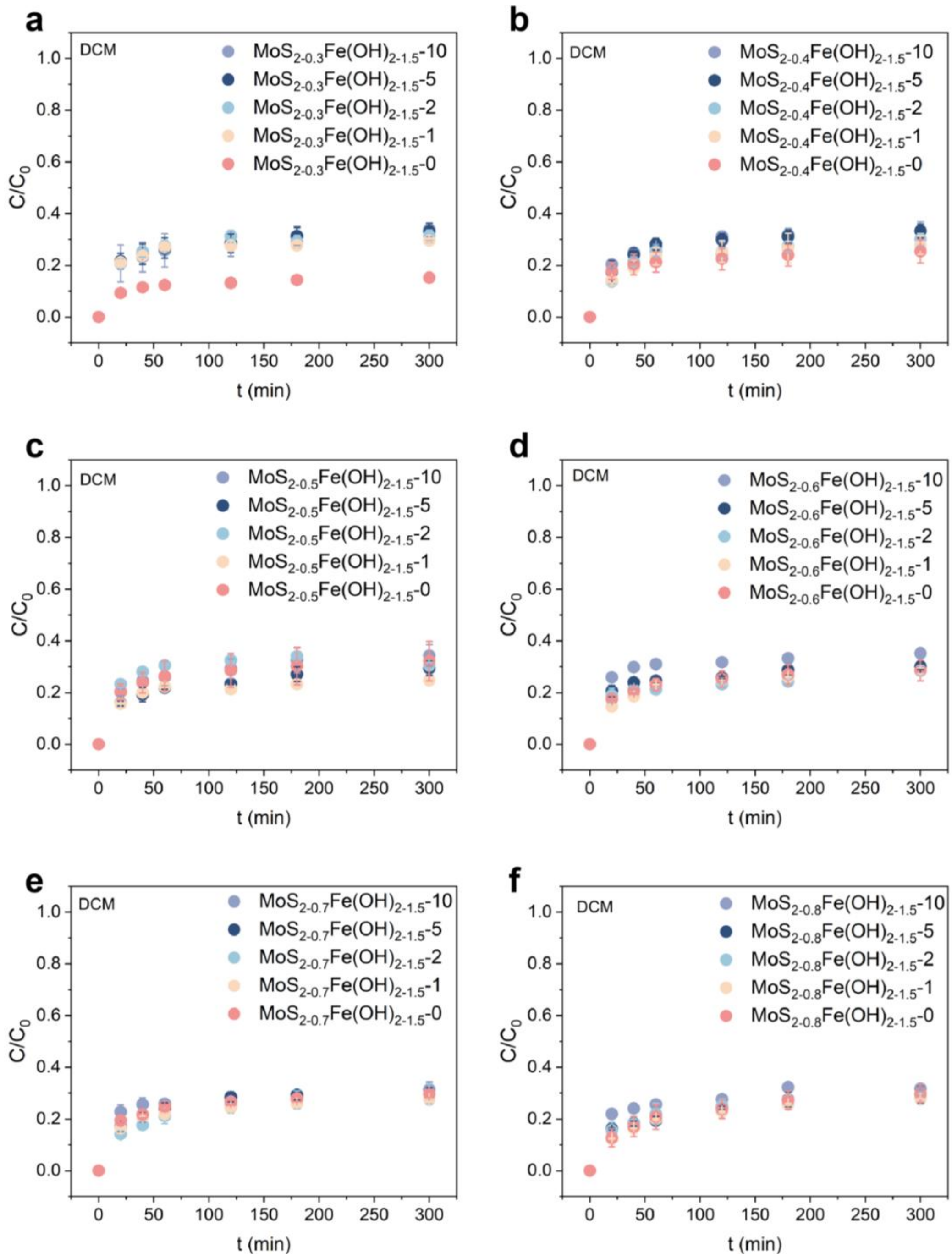

Figure S38. Dichloromethane generation during chloroform degradation by $MoS_2/Fe(OH)_{2\text{-}1.5}$. (a) $MoS_{2\text{-}0.3}/Fe(OH)_{2\text{-}1.5}$, (b) $MoS_{2\text{-}0.4}/Fe(OH)_{2\text{-}1.5}$, (c) $MoS_{2\text{-}0.5}/Fe(OH)_{2\text{-}1.5}$, (d) $MoS_{2\text{-}0.6}/Fe(OH)_{2\text{-}1.5}$, (e) $MoS_{2\text{-}0.7}/Fe(OH)_{2\text{-}1.5}$, (f) $MoS_{2\text{-}0.8}/Fe(OH)_{2\text{-}1.5}$. Data are presented as mean ± standard error (SE) from n = 2 independent experiments. Source data are provided in Source Data 2.

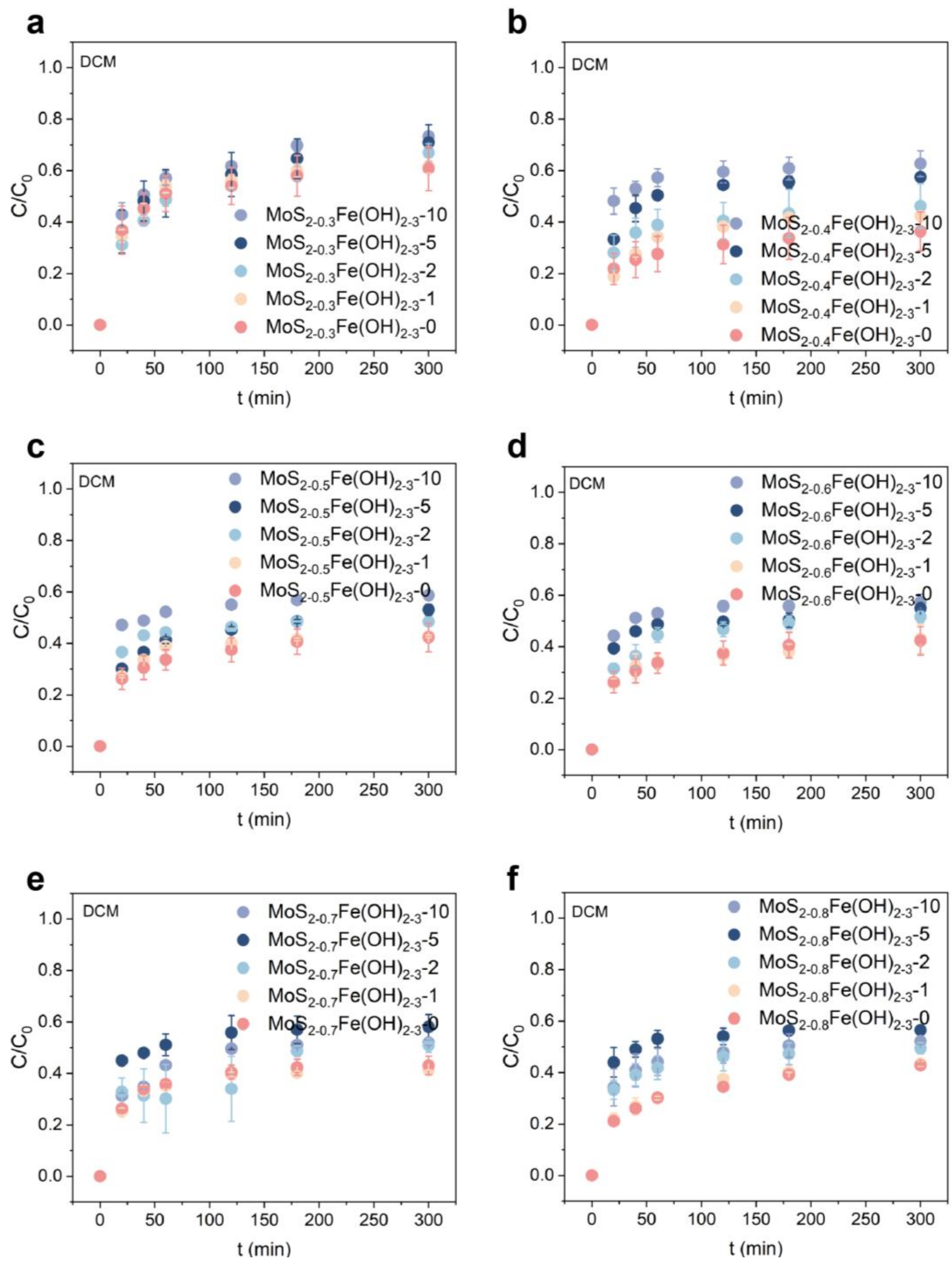

Figure S39. Dichloromethane generation during chloroform degradation by $MoS_2/Fe(OH)_{2-3}$. (a) $MoS_{2-0.3}/Fe(OH)_{2-3}$, (b) $MoS_{2-0.4}/Fe(OH)_{2-3}$, (c) $MoS_{2-0.5}/Fe(OH)_{2-3}$, (d) $MoS_{2-0.6}/Fe(OH)_{2-3}$, (e) $MoS_{2-0.7}/Fe(OH)_{2-3}$, (f) $MoS_{2-0.8}/Fe(OH)_{2-3}$. Data are presented as mean ± standard error (SE) from n = 2 independent experiments. Source data are provided in Source Data 2.

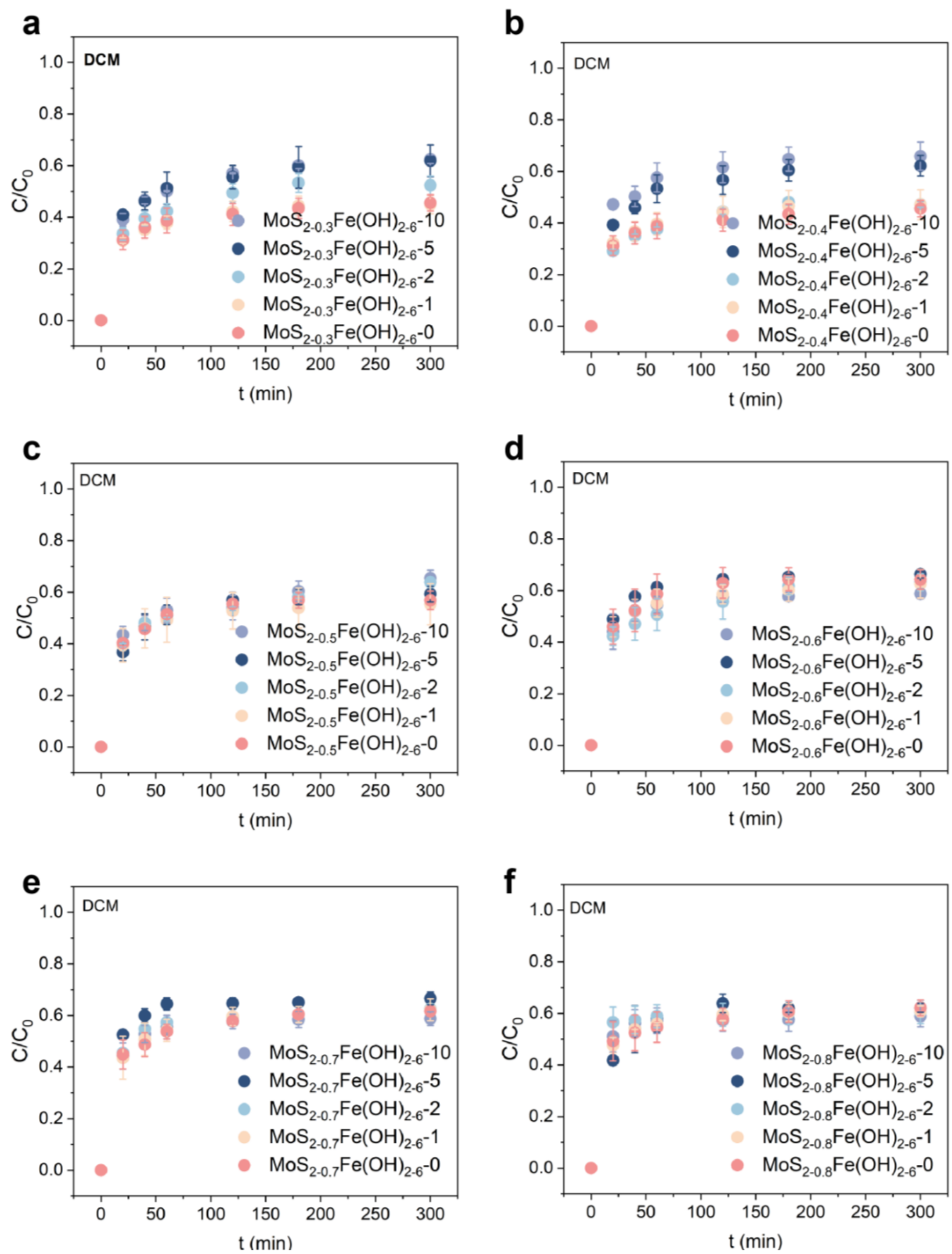

Figure S40. Dichloromethane generation during chloroform degradation by $MoS_2/Fe(OH)_{2\text{-}6}$. (a) $MoS_{2\text{-}0.3}/Fe(OH)_{2\text{-}6}$, (b) $MoS_{2\text{-}0.4}/Fe(OH)_{2\text{-}6}$, (c) $MoS_{2\text{-}0.5}/Fe(OH)_{2\text{-}6}$, (d) $MoS_{2\text{-}0.6}/Fe(OH)_{2\text{-}6}$, (e) $MoS_{2\text{-}0.7}/Fe(OH)_{2\text{-}6}$, (f) $MoS_{2\text{-}0.8}/Fe(OH)_{2\text{-}6}$. Data are presented as mean ± standard error (SE) from n = 2 independent experiments. Source data are provided in Source Data 2.

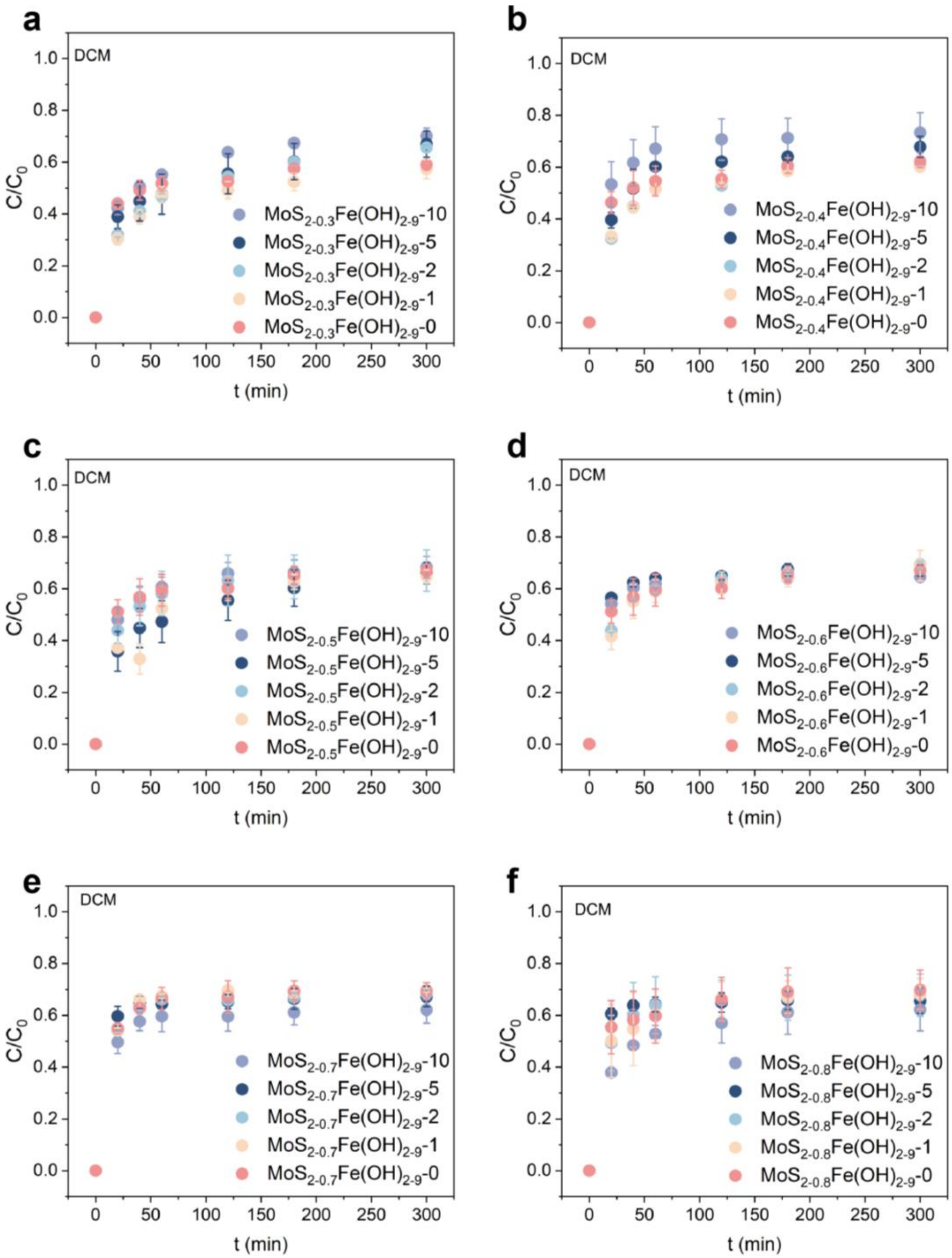

Figure S41. Dichloromethane generation during chloroform degradation by $MoS_2/Fe(OH)_{2\text{-}9}$. (a) $MoS_{2\text{-}0.3}/Fe(OH)_{2\text{-}9}$, (b) $MoS_{2\text{-}0.4}/Fe(OH)_{2\text{-}9}$, (c) $MoS_{2\text{-}0.5}/Fe(OH)_{2\text{-}9}$, (d) $MoS_{2\text{-}0.6}/Fe(OH)_{2\text{-}9}$, (e) $MoS_{2\text{-}0.7}/Fe(OH)_{2\text{-}9}$, (f) $MoS_{2\text{-}0.8}/Fe(OH)_{2\text{-}9}$. Data are presented as mean ± standard error (SE) from n = 2 independent experiments. Source data are provided in Source Data 2.

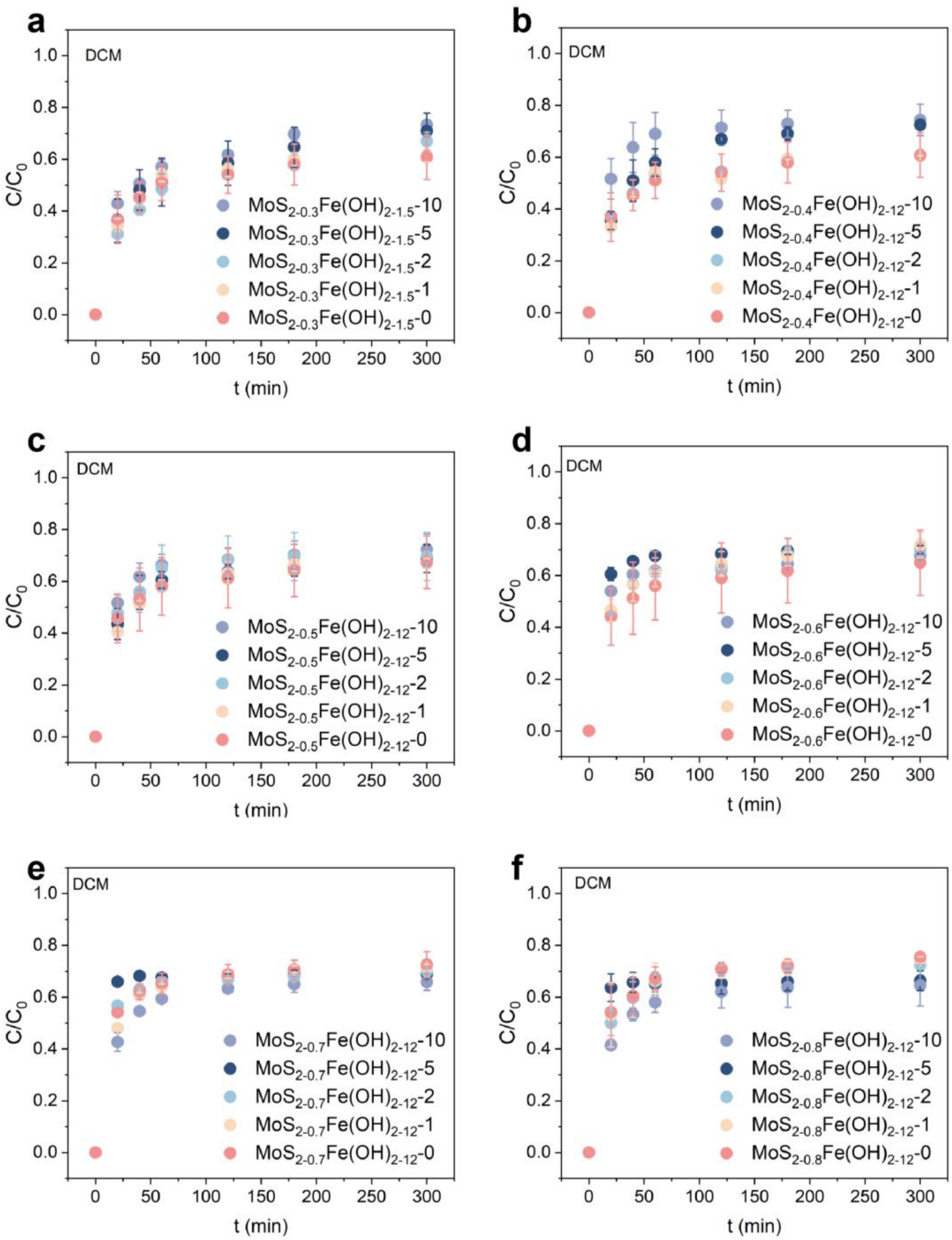

Figure S42. Dichloromethane generation during chloroform degradation by $MoS_2/Fe(OH)_{2-12}$. (a) $MoS_{2-0.3}/Fe(OH)_{2-12}$, (b) $MoS_{2-0.4}/Fe(OH)_{2-12}$, (c) $MoS_{2-0.5}/Fe(OH)_{2-12}$, (d) $MoS_{2-0.6}/Fe(OH)_{2-12}$, (e) $MoS_{2-0.7}/Fe(OH)_{2-12}$, (f) $MoS_{2-0.8}/Fe(OH)_{2-12}$. Data are presented as mean ± standard error (SE) from n = 2 independent experiments. Source data are provided in Source Data 2.

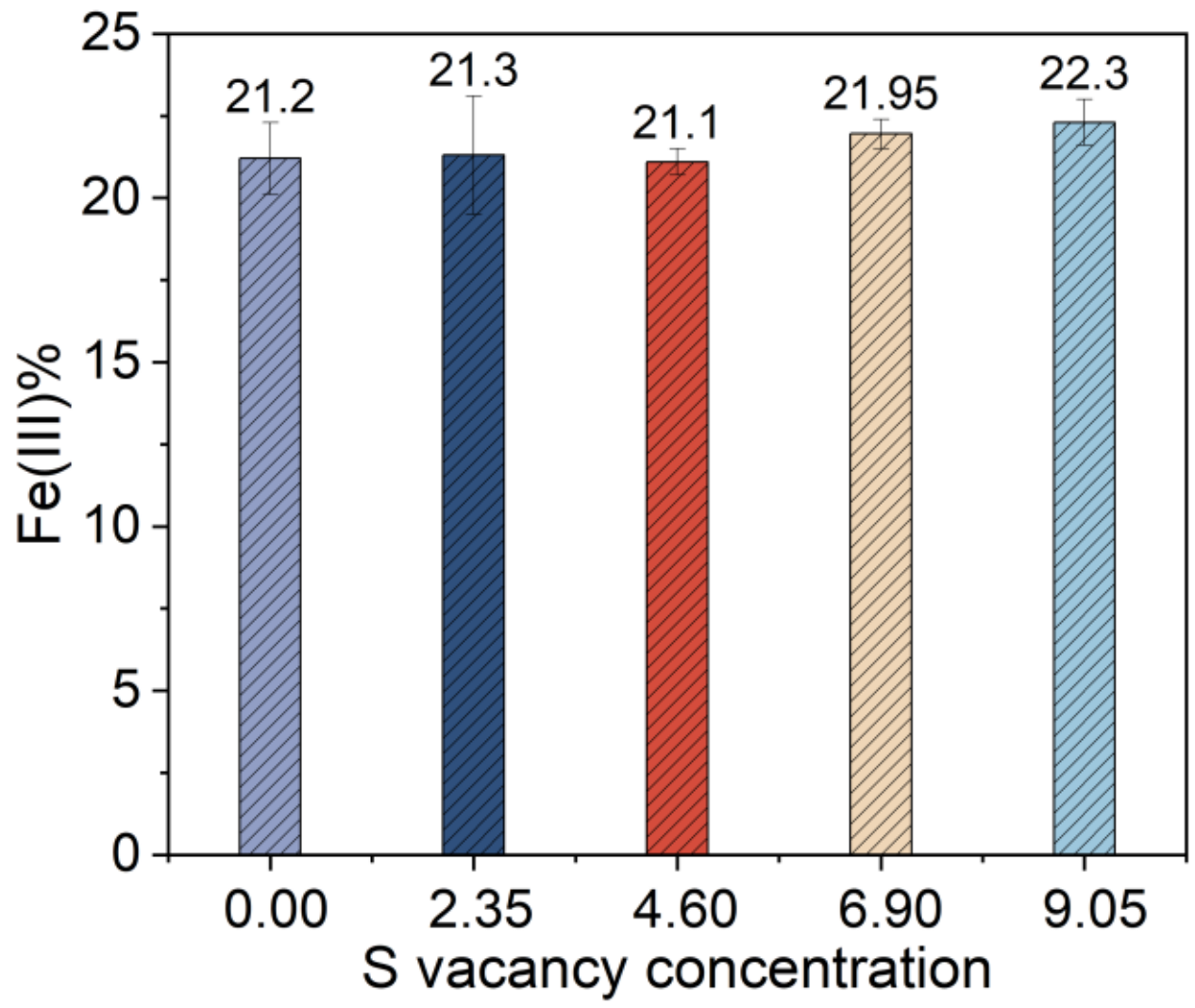

Figure S43. The concentration of Fe(III) after reaction under different initial S vacancy concentration. Bars represent mean ± standard error (SE) from n = 2 independent experiments. Points indicate individual measurements. Source data are provided in Source Data 2.

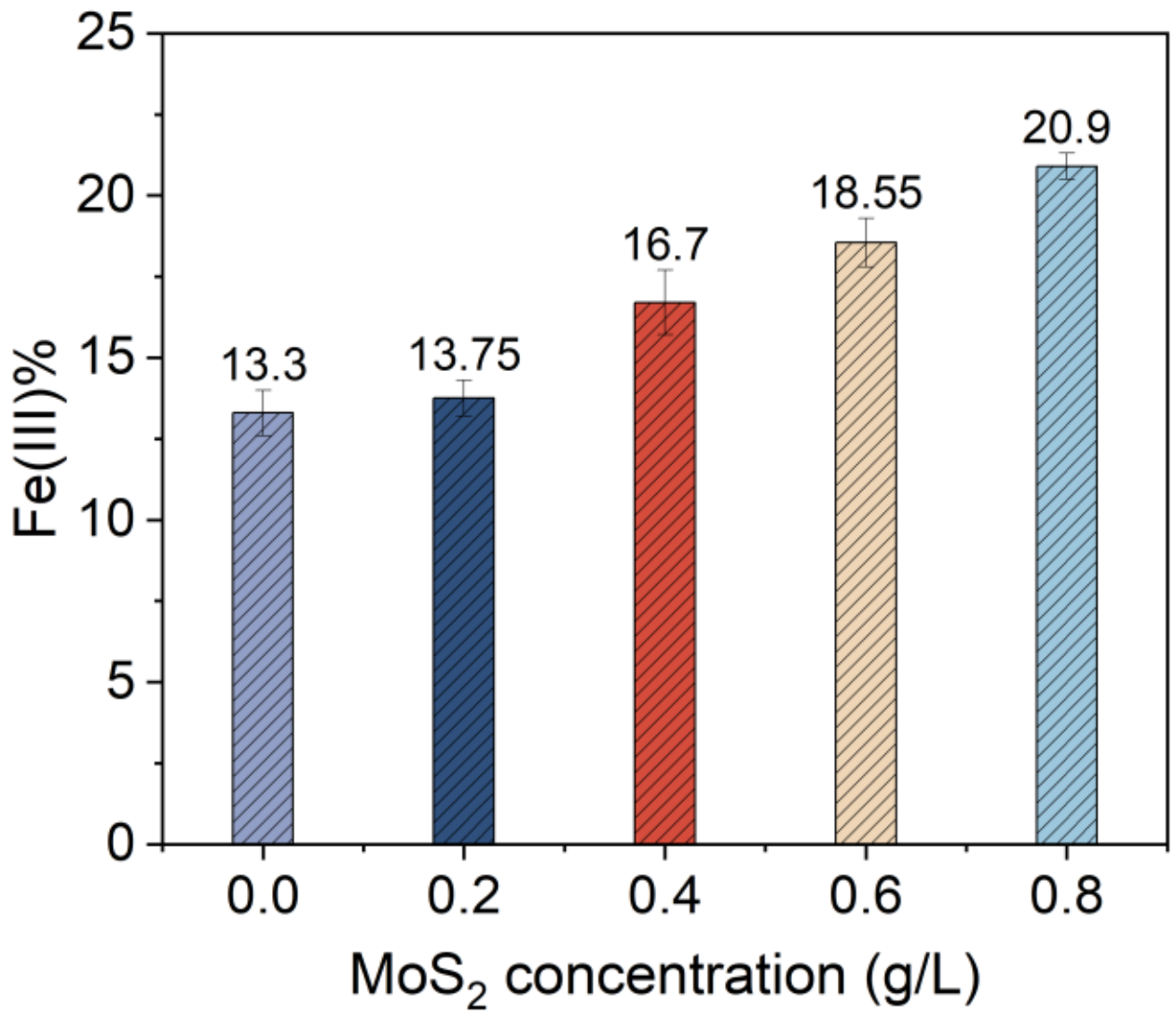

Figure S44. The concentration of Fe(III) after reaction under different initial MoS_2 concentrations. Bars represent mean ± standard error (SE) from n = 2 independent experiments. Points indicate individual measurements. Source data are provided in Source Data 2.

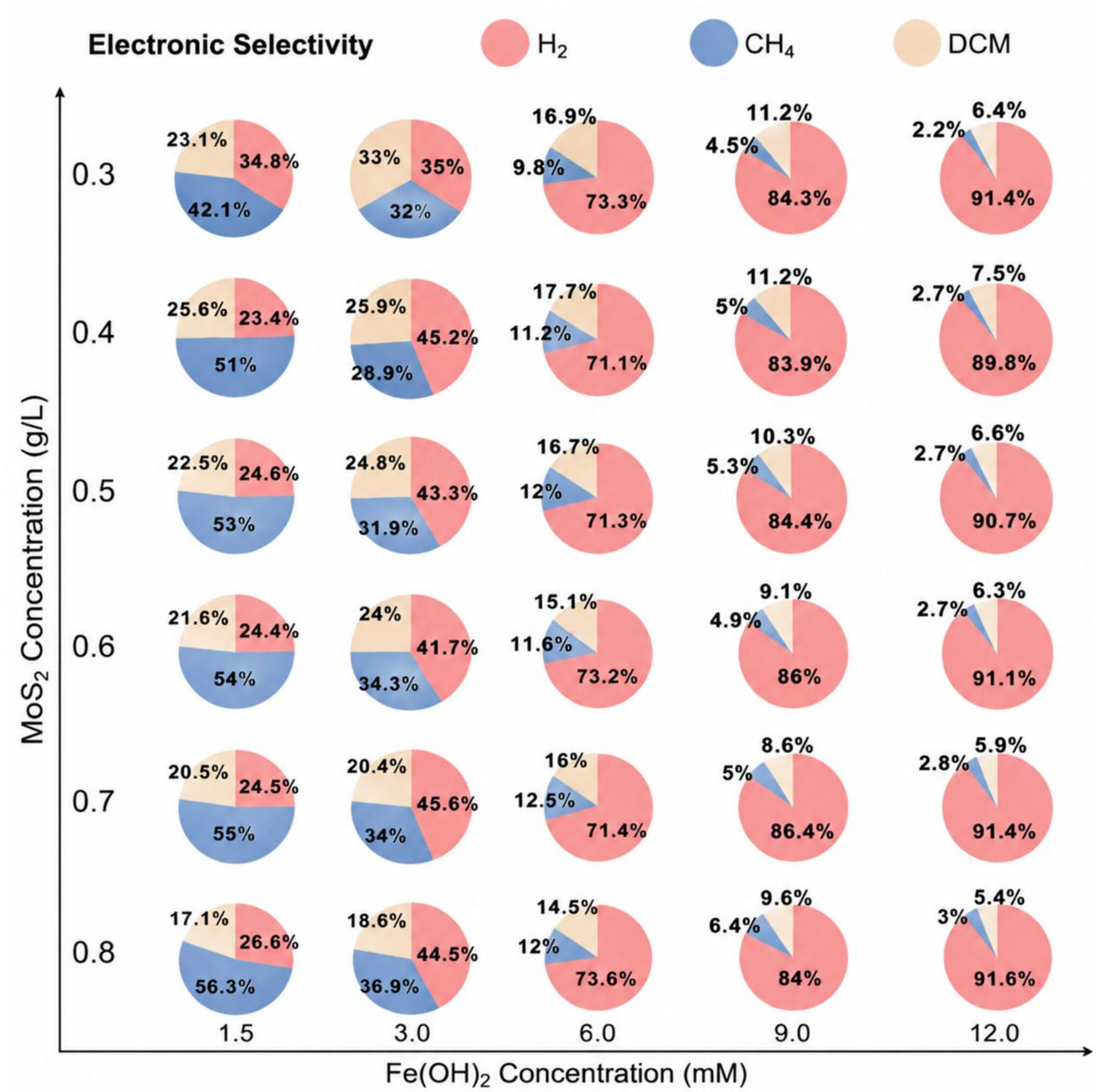

Figure S45. The effect of tuning MoS_2 concentration and $Fe(OH)_2$ concentration on the electron selectivity of products. Colours indicate the electron selectivity toward different products: blue, CH_4; yellow, DCM; and red, H_2. Source data are provided in Source Data 2.

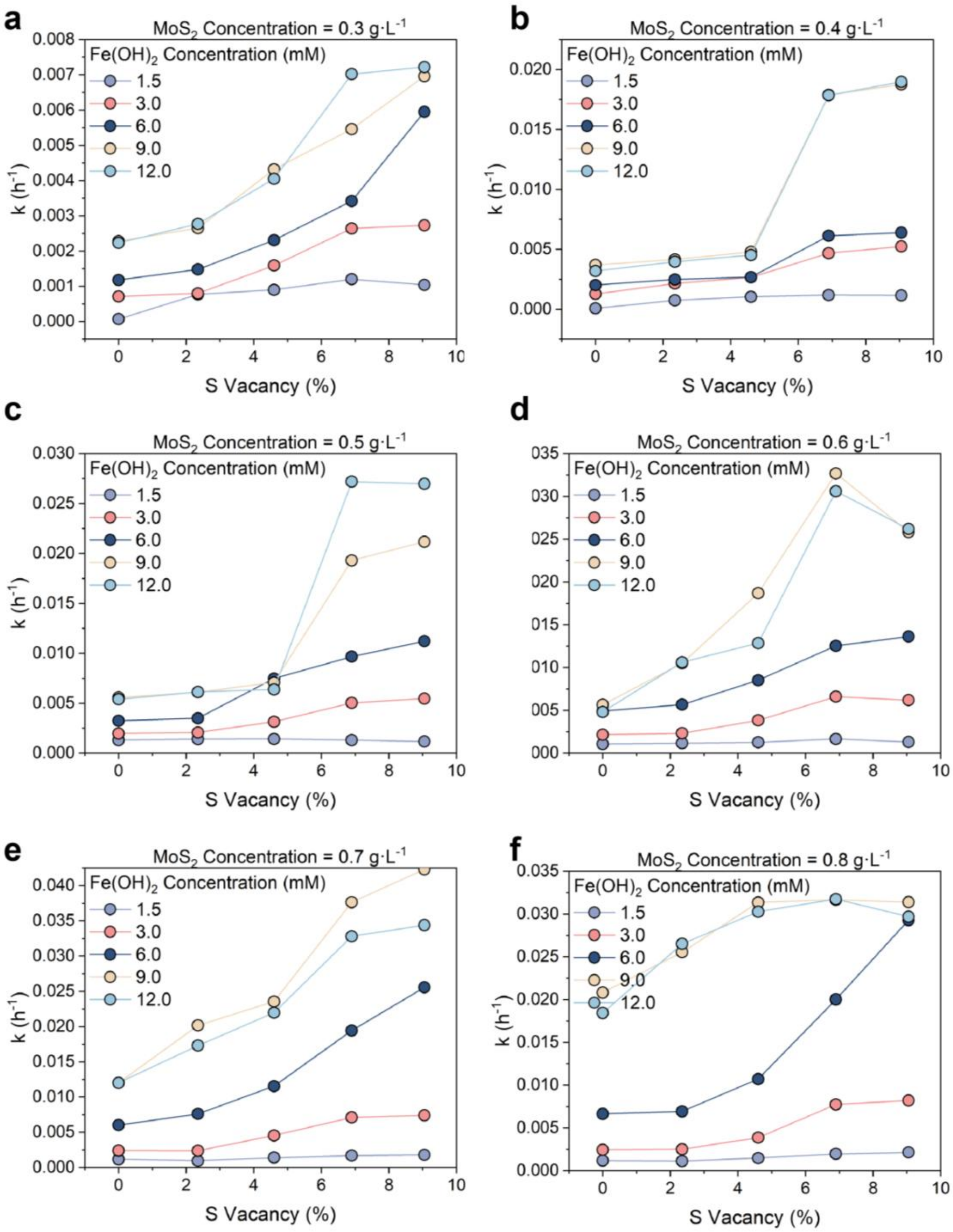

Figure S46. Correlation between sulfur-vacancy density and intrinsic pseudo-first-order rate constant (k_1) for chloroform degradation with different MoS_2 concentration. (a) 0.3 g·L^{-1}, (b) 0.4 g·L^{-1}, (c) 0.5 g·L^{-1}, (d) 0.6 g·L^{-1}, (e) 0.7 g·L^{-1} and (f) 0.8 g·L^{-1}. Source data are provided in Source Data 2.

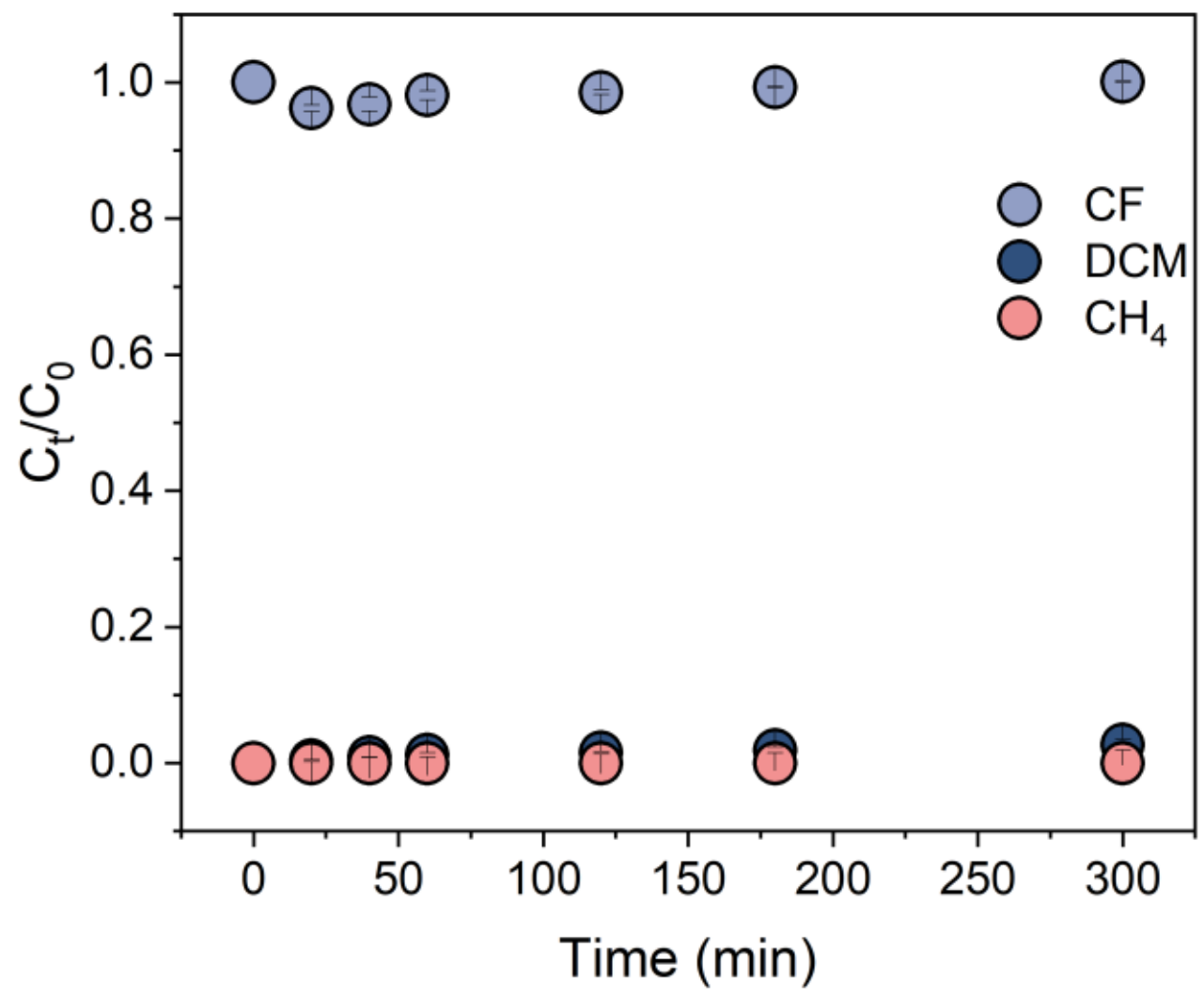

Figure S47. CF degradation by pure $Fe(OH)_2$ (12 mM). Data are presented as mean ± standard error (SE) from n = 2 independent experiments. Source data are provided in Source Data 2.

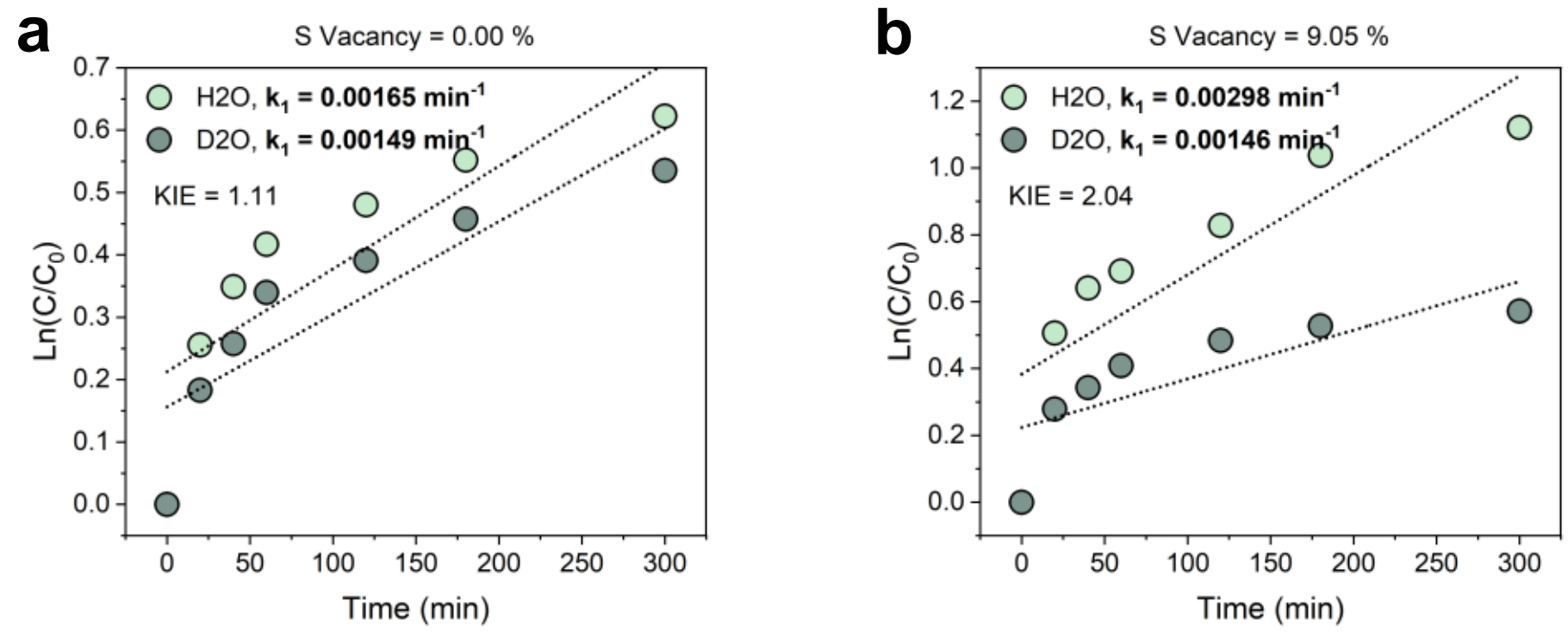

Figure S48. Kinetic isotope effect (KIE) of CF degradation in low and high S-vacancy-density groups. Panels a and b show the KIE results for the low S-vacancy-density group (0.00%) and high S-vacancy-density group (9.05%), respectively. Source data are provided in Source Data 2.

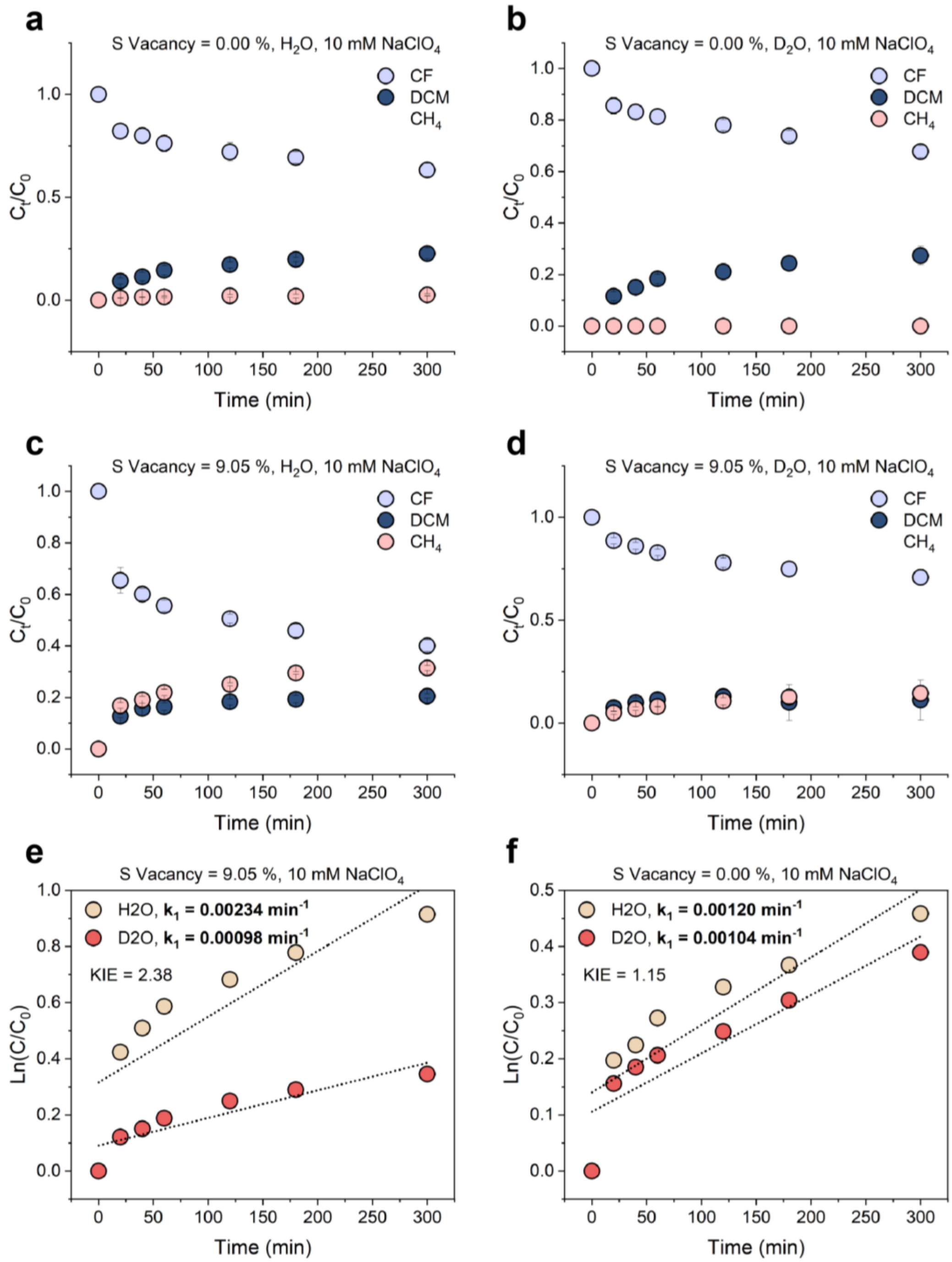

Figure S49. CF degradation curves. (a) Low S vacancy density group in H_2O with adding $NaClO_4$, (b) Low S vacancy density group in D_2O with adding $NaClO_4$, (c) High S vacancy density group in H_2O with adding $NaClO_4$, (d) High S vacancy density group in D_2O with adding $NaClO_4$, kinetic isotope effect (KIE) of (e) low S vacancy density (0.00%) and (f) high S vacancy density (9.05%) with adding $NaClO_4$ (10 mM). Data are presented as mean ± standard error (SE) from n = 2 independent experiments. Source data are provided in Source Data 2.

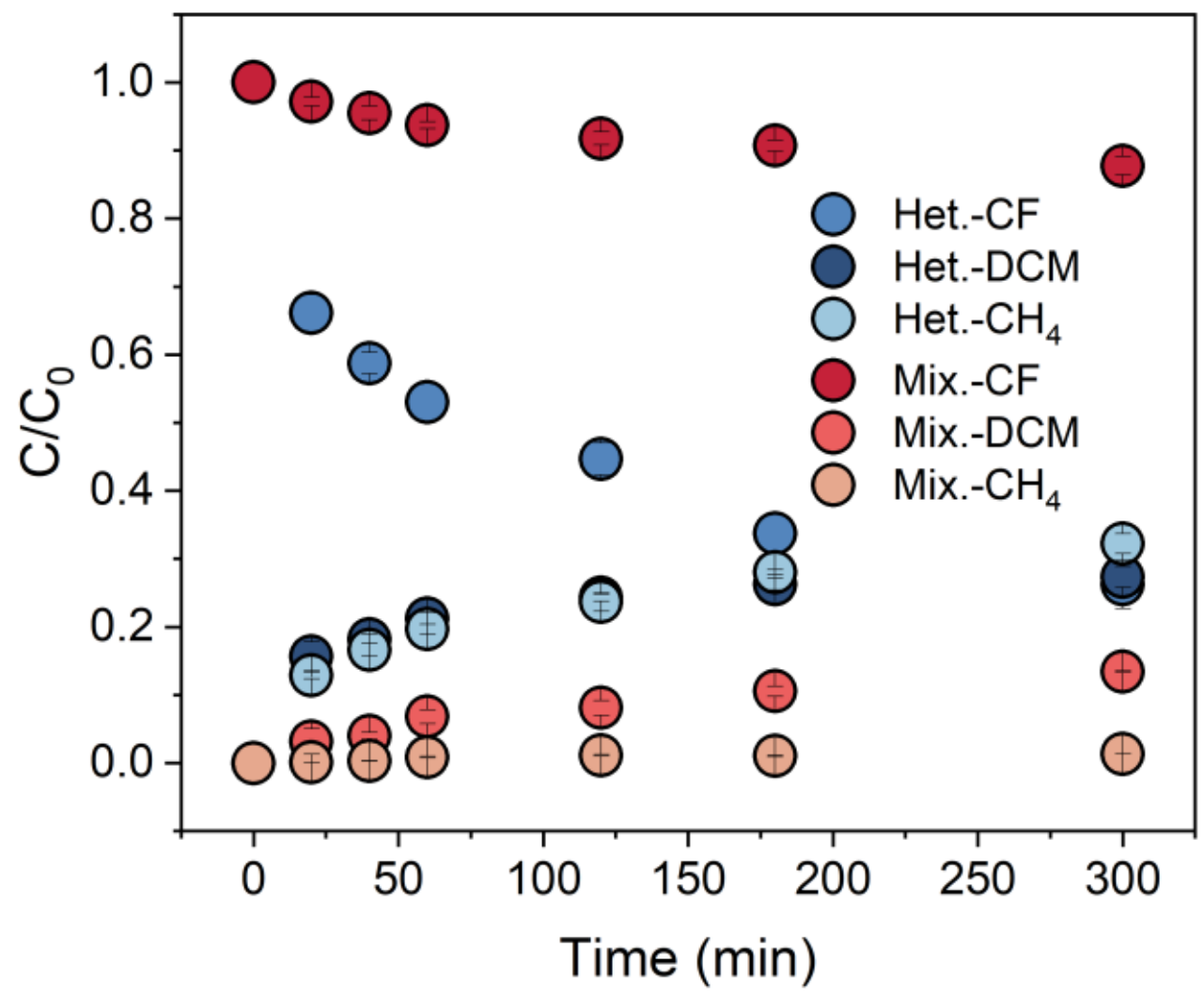

Figure S50. Comparison of CF degradation by the $MoS_2/Fe(OH)_2$ heterojunction and the physical-mixture control. Data are presented as the mean ± standard error (SE) from n = 2 independent experiments, showing the effect of heterojunction formation on catalytic performance. Source data are provided in Source Data 2.

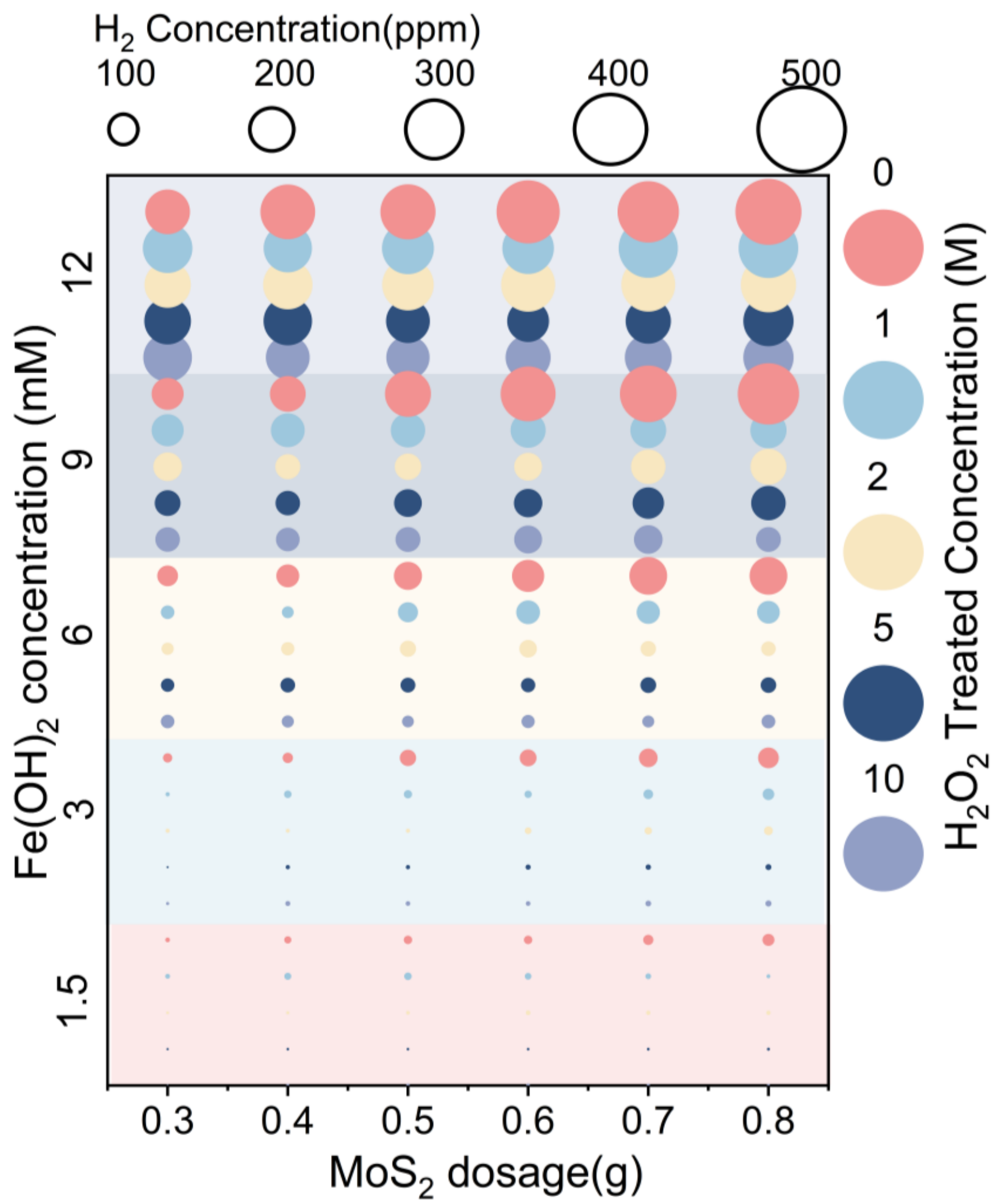

Figure S51. Hydrogen evolution during the reaction. The experiments quantify H_2 generation under the tested conditions, providing a measure of the competing hydrogen evolution pathway. Source data are provided in Source Data 2.

Supporting Information Tables

Table S1. Mo/S ratio in MoS_2 etched with different concentrations of H_2O_2.

H_2O_2 Concentration (M)	Mo/S	S Vacancy (%)
0	2.052	0.00
1	1.953	2.35
2	1.908	4.60
5	1.862	6.90
10	1.819	9.05

Table S2. Structural parameters obtained from EXAFS fitting in R space.

Sample	Path	CN	R(Å)	σ^2 (Å^2)	ΔE_0(eV)	R factor
$MoS_2/Fe(OH)_2$	Fe-O	5.26	2.03	0.013	-2.95	0.028
$Fe(OH)_2$	Fe-O	6.72	2.01	0.009	-4.72	0.024

Table S3. Mössbauer spectroscopy parameters of $Fe(OH)_2$ and $Fe(OH)_2/MoS_2$

Sample Name	Component	IS(mm^{-1})	QS(mm^{-1})	Γ(mm^{-1})	Area(%)
$Fe(OH)_2$	Doublet (1)	1.31	3.03	0.47	55.80
	Doublet (2)	1.28	1.74	0.71	30.30
	Doublet (3)	0.25	0.97	0.32	14.00
$MoS_2/Fe(OH)_2$	Doublet (1)	1.30	3.09	0.39	38.10
	Doublet (2)	1.39	2.15	0.87	46.00
	Doublet (3)	0.21	0.90	0.36	15.90

Table S4. Pseudo-first-order rate constant and corresponding goodness of fit (R^2) for chloroform degradation at a H_2O_2 etching concentration of 10 M.

MoS_2 Concentration	$Fe(OH)_2$ Concentration (mM)	k_1 (min^{-1})	R^2
0.3	1.5	0.00104	0.7492
	3.0	0.00273	0.9003
	6.0	0.00595	0.8976
	9.0	0.00696	0.9801
	12.0	0.00722	0.9897
0.4	1.5	0.00115	0.8102
	3.0	0.00523	0.9013
	6.0	0.00639	0.9942
	9.0	0.01876	0.9936
	12.0	0.01898	0.9933
0.5	1.5	0.00116	0.7767
	3.0	0.00547	0.8765
	6.0	0.01121	0.9947
	9.0	0.02117	0.9972
	12.0	0.02698	0.9794
0.6	1.5	0.00130	0.8007
	3.0	0.00619	0.8511
	6.0	0.01362	0.9613
	9.0	0.02583	0.8583
	12.0	0.02620	0.8756
0.7	1.5	0.00180	0.9261
	3.0	0.00741	0.8718
	6.0	0.02556	0.9806
	9.0	0.04230	0.8342
	12.0	0.03438	0.7932
0.8	1.5	0.00215	0.9164
	3.0	0.00821	0.8338
	6.0	0.02928	0.9866
	9.0	0.03139	0.7994
	12.0	0.02969	0.7937

Table S5. Pseudo-first-order rate constant and corresponding goodness of fit (R^2) for chloroform degradation at a H_2O_2 etching concentration of 5 M.

MoS_2 Concentration	$Fe(OH)_2$ Concentration (mM)	k_1 (min^{-1})	R^2
0.3	1.5	0.00120	0.7598
	3.0	0.00264	0.9205
	6.0	0.00342	0.9231
	9.0	0.00546	0.9935
	12.0	0.00702	0.9970
0.4	1.5	0.00118	0.8432
	3.0	0.00467	0.9414
	6.0	0.00612	0.9818
	9.0	0.01788	0.9884
	12.0	0.01785	0.9964
0.5	1.5	0.00131	0.8282
	3.0	0.00503	0.8483
	6.0	0.00967	0.9886
	9.0	0.01930	0.9791
	12.0	0.02719	0.9945
0.6	1.5	0.00166	0.8463
	3.0	0.00661	0.8679
	6.0	0.01254	0.9483
	9.0	0.03268	0.9397
	12.0	0.03060	0.9360
0.7	1.5	0.00169	0.8712
	3.0	0.00711	0.8883
	6.0	0.01943	0.9345
	9.0	0.03764	0.8340
	12.0	0.03281	0.8986
0.8	1.5	0.00197	0.9307
	3.0	0.00774	0.8830
	6.0	0.02002	0.9524
	9.0	0.03165	0.8827
	12.0	0.03173	0.9058

Table S6. Pseudo-first-order rate constant and corresponding goodness of fit (R^2) for chloroform degradation at a H_2O_2 etching concentration of 2 M.

MoS_2 Concentration	$Fe(OH)_2$ Concentration (mM)	k_1 (min^{-1})	R^2
0.3	1.5	0.00090	0.7390
	3.0	0.00159	0.8502
	6.0	0.00231	0.9272
	9.0	0.00432	0.9746
	12.0	0.00405	0.9668
0.4	1.5	0.00104	0.8608
	3.0	0.00266	0.8890
	6.0	0.00268	0.9326
	9.0	0.00478	0.9283
	12.0	0.00452	0.7818
0.5	1.5	0.00143	0.8679
	3.0	0.00314	0.7811
	6.0	0.00747	0.9771
	9.0	0.00708	0.9307
	12.0	0.00638	0.9087
0.6	1.5	0.00126	0.8871
	3.0	0.00383	0.8884
	6.0	0.00852	0.9154
	9.0	0.01869	0.9972
	12.0	0.01286	0.9419
0.7	1.5	0.00141	0.9072
	3.0	0.00455	0.8803
	6.0	0.01154	0.9626
	9.0	0.02355	0.9910
	12.0	0.02197	0.9814
0.8	1.5	0.00149	0.8709
	3.0	0.00387	0.7055
	6.0	0.01072	0.9260
	9.0	0.03136	0.9758
	12.0	0.03027	0.9247

Table S7. Pseudo-first-order rate constant and corresponding goodness of fit (R^2) for chloroform degradation at a H_2O_2 etching concentration of 1 M.

MoS_2 Concentration	$Fe(OH)_2$ Concentration (mM)	k_1 (min^{-1})	R^2
0.3	1.5	0.00077	0.7524
	3.0	0.00080	0.8903
	6.0	0.00148	0.8878
	9.0	0.00265	0.9508
	12.0	0.00277	0.9426
0.4	1.5	0.00074	0.7983
	3.0	0.00215	0.8435
	6.0	0.00248	0.8934
	9.0	0.00415	0.9157
	12.0	0.00396	0.8939
0.5	1.5	0.00141	0.8740
	3.0	0.00207	0.8089
	6.0	0.00350	0.8194
	9.0	0.00610	0.8272
	12.0	0.00613	0.8509
0.6	1.5	0.00116	0.8550
	3.0	0.00233	0.8423
	6.0	0.00567	0.9738
	9.0	0.01051	0.9749
	12.0	0.01062	0.9786
0.7	1.5	0.00096	0.8485
	3.0	0.00238	0.8758
	6.0	0.00761	0.9544
	9.0	0.02017	0.9989
	12.0	0.01730	0.9975
0.8	1.5	0.00113	0.9098
	3.0	0.00252	0.8522
	6.0	0.00693	0.9256
	9.0	0.02554	0.9971
	12.0	0.02651	0.9951

Table S8. Pseudo-first-order rate constant and corresponding goodness of fit (R^2) for chloroform degradation without etching process.

MoS_2 Concentration	$Fe(OH)_2$ Concentration (mM)	k_1 (min^{-1})	R^2
0.3	1.5	0.00007	0.2814
	3.0	0.00071	0.8671
	6.0	0.00118	0.8353
	9.0	0.00228	0.9262
	12.0	0.00223	0.9008
0.4	1.5	0.00007	0.8832
	3.0	0.00127	0.9492
	6.0	0.00201	0.9807
	9.0	0.00369	0.9166
	12.0	0.00319	0.9309
0.5	1.5	0.00131	0.8843
	3.0	0.00197	0.8507
	6.0	0.00324	0.8557
	9.0	0.00558	0.8446
	12.0	0.00539	0.8476
0.6	1.5	0.00107	0.8238
	3.0	0.00216	0.8302
	6.0	0.00492	0.9367
	9.0	0.00566	0.9265
	12.0	0.00481	0.8947
0.7	1.5	0.00118	0.8863
	3.0	0.00240	0.8623
	6.0	0.00603	0.9350
	9.0	0.01201	0.9776
	12.0	0.01203	0.9832
0.8	1.5	0.00119	0.8809
	3.0	0.00245	0.9046
	6.0	0.00666	0.9516
	9.0	0.02082	0.9961
	12.0	0.01844	0.9946

Table S9. Bader charge analysis of $MoS_2/Fe(OH)_2$ without S vacancy.

1	Fe	6.59172	33	O	7.31599	65	H	0.3683	97	S	6.61589
2	Fe	6.58691	34	O	7.26342	66	H	0.42078	98	S	6.56707
3	Fe	6.59144	35	O	7.32016	67	H	0.36385	99	S	6.62347
4	Fe	6.58688	36	O	7.26485	68	H	0.41925	100	S	6.56895
5	Fe	6.58684	37	O	7.31694	69	H	0.36791	101	S	6.61685
6	Fe	6.58562	38	O	7.26311	70	H	0.42103	102	S	6.56723
7	Fe	6.58754	39	O	7.3189	71	H	0.36511	103	S	6.62325
8	Fe	6.58532	40	O	7.26622	72	H	0.41803	104	S	6.56916
9	Fe	6.59136	41	O	7.31524	73	H	0.36945	105	S	6.61998
10	Fe	6.58658	42	O	7.25981	74	H	0.42515	106	S	6.56745
11	Fe	6.59167	43	O	7.31477	75	H	0.3692	107	S	6.61455
12	Fe	6.58714	44	O	7.26346	76	H	0.42007	108	S	6.56755
13	Fe	6.58777	45	O	7.31546	77	H	0.36932	109	S	6.61897
14	Fe	6.58546	46	O	7.25929	78	H	0.42602	110	S	6.56714
15	Fe	6.58703	47	O	7.31345	79	H	0.37028	111	S	6.615
16	Fe	6.58573	48	O	7.26318	80	H	0.42063	112	S	6.5675
17	O	7.31772	49	H	0.36741	81	Mo	12.84807	113	S	6.61759
18	O	7.26232	50	H	0.42193	82	Mo	12.84812	114	S	6.56746
19	O	7.31951	51	H	0.36432	83	Mo	12.848	115	S	6.62345
20	O	7.26585	52	H	0.41833	84	Mo	12.8484	116	S	6.56908
21	O	7.31712	53	H	0.36766	85	Mo	12.85993	117	S	6.61636
22	O	7.26514	54	H	0.41888	86	Mo	12.84894	118	S	6.56729
23	O	7.32001	55	H	0.36411	87	Mo	12.86023	119	S	6.62387
24	O	7.26516	56	H	0.4189	88	Mo	12.84802	120	S	6.56931
25	O	7.3155	57	H	0.3691	89	Mo	12.84759	121	S	6.61983
26	O	7.25879	58	H	0.42668	90	Mo	12.84875	122	S	6.5676
27	O	7.31533	59	H	0.36839	91	Mo	12.84779	123	S	6.61531
28	O	7.26325	60	H	0.42019	92	Mo	12.84683	124	S	6.56763
29	O	7.31551	61	H	0.36866	93	Mo	12.85949	125	S	6.61978
30	O	7.259	62	H	0.42636	94	Mo	12.8476	126	S	6.5673
31	O	7.3141	63	H	0.36961	95	Mo	12.85953	127	S	6.615
32	O	7.26356	64	H	0.42022	96	Mo	12.84804	128	S	6.56742

Table S10. Bader charge analysis of $MoS_2/Fe(OH)_2$ *with 3.1% S vacancy*.

1	Fe	6.58848	33	O	7.316311	65	H	0.364774	97	S	6.655713
2	Fe	6.575592	34	O	7.272314	66	H	0.411577	98	S	6.578631
3	Fe	6.582111	35	O	7.314893	67	H	0.365434	99	S	6.645348
4	Fe	6.578125	36	O	7.274544	68	H	0.408583	100	S	6.57425
5	Fe	6.575417	37	O	7.312049	69	H	0.371073	101	S	6.622129
6	Fe	6.57383	38	O	7.274085	70	H	0.408666	102	S	6.581033
7	Fe	6.575422	39	O	7.325291	71	H	0.359624	103	S	6.656565
8	Fe	6.57668	40	O	7.274078	72	H	0.408249	104	S	6.580164
9	Fe	6.589542	41	O	7.3235	73	H	0.361505	105	S	6.646425
10	Fe	6.575932	42	O	7.273616	74	H	0.408923	106	S	6.577499
11	Fe	6.586881	43	O	7.31551	75	H	0.365792	107	S	6.655658
12	Fe	6.567889	44	O	7.274552	76	H	0.408492	108	S	6.577668
13	Fe	6.567624	45	O	7.317118	77	H	0.363754	109	S	6.615471
14	Fe	6.577479	46	O	7.280422	78	H	0.404682	110	S	6.610125
15	Fe	6.578165	47	O	7.308947	79	H	0.374207	111	S	6.612143
16	Fe	6.559115	48	O	7.272005	80	H	0.409932	112	S	6.634414
17	O	7.312469	49	H	0.367549	81	Mo	12.826384	113	S	6.598402
18	O	7.273363	50	H	0.410411	82	Mo	12.852116	114	S	6.611875
19	O	7.315248	51	H	0.365286	83	Mo	12.834952	115	S	6.648052
20	O	7.279508	52	H	0.404242	84	Mo	12.812777	116	S	6.578096
21	O	7.312853	53	H	0.368057	85	Mo	12.81858	117	S	6.625168
22	O	7.275368	54	H	0.408537	86	Mo	12.826948	118	S	6.623139
23	O	7.315881	55	H	0.36373	87	Mo	12.830503	119	S	6.706032
24	O	7.27522	56	H	0.408079	88	Mo	12.83094	120	S	6.605834
25	O	7.317534	57	H	0.363905	89	Mo	12.84206	121	S	6.607774
26	O	7.277211	58	H	0.40562	90	Mo	12.828243	122	S	6.620398
27	O	7.312944	59	H	0.367815	91	Mo	12.950866	123	S	6.579267
28	O	7.27112	60	H	0.412107	92	Mo	12.987984	124	S	6.643187
29	O	7.322624	61	H	0.36257	93	Mo	12.845437	125	S	6.586628
30	O	7.275024	62	H	0.408126	94	Mo	12.836076	126	S	6.615618
31	O	7.310744	63	H	0.370889	95	Mo	12.819362	127	S	6.623089
32	O	7.271429	64	H	0.411687	96	Mo	12.992006			

Table S11. Bader charge analysis of $MoS_2/Fe(OH)_2$ *with 6.3% S vacancy*.

1	Fe	6.589054	33	O	7.3136	65	H	0.368041	97	S	6.612803
2	Fe	6.565663	34	O	7.277796	66	H	0.404733	98	S	6.614654
3	Fe	6.579933	35	O	7.311434	67	H	0.36867	99	S	6.706772
4	Fe	6.572069	36	O	7.27453	68	H	0.408831	100	S	6.619656
5	Fe	6.569968	37	O	7.31568	69	H	0.364681	101	S	6.613129
6	Fe	6.562295	38	O	7.274093	70	H	0.408565	102	S	6.661707
7	Fe	6.56457	39	O	7.319237	71	H	0.36226	103	S	6.587965
8	Fe	6.567671	40	O	7.277481	72	H	0.406129	104	S	6.663092
9	Fe	6.580699	41	O	7.313905	73	H	0.369598	105	S	6.570486
10	Fe	6.571027	42	O	7.279681	74	H	0.40249	106	S	6.612814
11	Fe	6.589555	43	O	7.312779	75	H	0.368884	107	S	6.614548
12	Fe	6.565722	44	O	7.278521	76	H	0.404366	108	S	6.599595
13	Fe	6.564185	45	O	7.316363	77	H	0.36398	109	S	6.627293
14	Fe	6.568031	46	O	7.274346	78	H	0.408244	110	S	6.619565
15	Fe	6.57075	47	O	7.314413	79	H	0.366423	111	S	6.614121
16	Fe	6.561844	48	O	7.272775	80	H	0.411075	112	S	6.619675
17	O	7.316011	49	H	0.364295	81	Mo	12.991222	113	S	6.613898
18	O	7.274643	50	H	0.408565	82	Mo	12.992956	114	S	6.661895
19	O	7.318871	51	H	0.362505	83	Mo	12.815376	115	S	6.588159
20	O	7.277704	52	H	0.405655	84	Mo	12.811151	116	S	6.612536
21	O	7.313811	53	H	0.367711	85	Mo	12.854369	117	S	6.614419
22	O	7.276524	54	H	0.405925	86	Mo	12.991153	118	S	6.707164
23	O	7.311973	55	H	0.36781	87	Mo	12.856307	119	S	6.599606
24	O	7.274746	56	H	0.408781	88	Mo	12.814288	120	S	6.627074
25	O	7.316825	57	H	0.363633	89	Mo	12.815595	121	S	6.618712
26	O	7.274201	58	H	0.40819	90	Mo	12.810471	122	S	6.615213
27	O	7.314597	59	H	0.366042	91	Mo	12.990518	123	S	6.663692
28	O	7.27182	60	H	0.411642	92	Mo	12.99296	124	S	6.570466
29	O	7.31401	61	H	0.369426	93	Mo	12.856182	125	S	6.611981
30	O	7.280776	62	H	0.401379	94	Mo	12.814391	126	S	6.614905
31	O	7.313792	63	H	0.3682	95	Mo	12.855602			
32	O	7.278293	64	H	0.404735	96	Mo	12.991385			

Table S12. Bader charge analysis of $MoS_2/Fe(OH)_2$ *with 9.4% S vacancy*.

1	Fe	6.600548	33	O	7.25578	65	H	0.396315	97	S	6.594982
2	Fe	6.609122	34	O	7.265513	66	H	0.398041	98	S	6.634889
3	Fe	6.607405	35	O	7.230929	67	H	0.422276	99	S	6.72588
4	Fe	6.589736	36	O	7.320772	68	H	0.369012	100	S	6.644073
5	Fe	6.59643	37	O	7.29143	69	H	0.39358	101	S	6.623993
6	Fe	6.509626	38	O	7.284192	70	H	0.405546	102	S	6.732452
7	Fe	6.569681	39	O	7.272886	71	H	0.409584	103	S	6.626767
8	Fe	6.56951	40	O	7.311721	72	H	0.37781	104	S	6.621352
9	Fe	6.610148	41	O	7.330514	73	H	0.357166	105	S	6.622715
10	Fe	6.608266	42	O	7.267873	74	H	0.39363	106	S	6.623928
11	Fe	6.605023	43	O	7.23633	75	H	0.416272	107	S	6.621944
12	Fe	6.565981	44	O	7.25171	76	H	0.411874	108	S	6.61917
13	Fe	6.608226	45	O	7.331801	77	H	0.356355	109	S	6.63954
14	Fe	6.212727	46	O	7.273438	78	H	0.415207	110	S	6.618695
15	Fe	6.609418	47	O	7.261255	79	H	0.422417	111	S	6.631647
16	Fe	6.587022	48	O	7.284145	80	H	0.408032	112	S	6.615926
17	O	7.284769	49	H	0.396214	81	Mo	12.990837	113	S	6.658812
18	O	7.269124	50	H	0.417872	82	Mo	12.958161	114	S	6.5788
19	O	7.256017	51	H	0.425822	83	Mo	12.959785	115	S	6.622868
20	O	7.255951	52	H	0.431691	84	Mo	12.987622	116	S	6.624124
21	O	7.276488	53	H	0.4078	85	Mo	12.841639	117	S	6.729372
22	O	7.263624	54	H	0.425009	86	Mo	12.960966	118	S	6.603696
23	O	7.313642	55	H	0.372013	87	Mo	12.851611	119	S	6.653487
24	O	7.286301	56	H	0.398732	88	Mo	12.956989	120	S	6.628731
25	O	7.264479	57	H	0.423205	89	Mo	12.853781	121	S	6.617083
26	O	7.261889	58	H	0.427161	90	Mo	12.862763	122	S	6.624839
27	O	7.287646	59	H	0.394608	91	Mo	12.95839	123	S	6.620891
28	O	7.307165	60	H	0.381888	92	Mo	12.959309	124	S	6.587786
29	O	7.280153	61	H	0.401211	93	Mo	12.839705	125	S	6.636106
30	O	7.253534	62	H	0.433097	94	Mo	12.851717			
31	O	7.316582	63	H	0.37006	95	Mo	12.841335			
32	O	7.271214	64	H	0.418159	96	Mo	12.986381			

SI References

1. Cui Z. J., Zhang X. J., Hu B. B., Zhu L. D. & Huang L. Z. CO_2 geological sequestration can remove emerging contaminants in groundwater: The important role of secondary mineral carbonates. *Water Res.* **277,** 123293 (2025).
2. Kresse G. & Hafner J. Ab initio molecular dynamics for liquid metals. *Phys. Rev. B* **47,** 558-561 (1993).
3. Blochl P. E. Projector augmented-wave method. *Phys. Rev. B* **50,** 17953-17979 (1994).
4. Kresse G. & Joubert D. From ultrasoft pseudopotentials to the projector augmented-wave method. *Phys. Rev. B* **59,** 1758-1775 (1999).
5. Perdew J. P., Burke K. & Ernzerhof M. Generalized Gradient Approximation Made Simple. *Phys. Rev. Lett.* **77,** 3865-3868 (1996).
6. Ernzerhof M. & Scuseria G. E. Assessment of the Perdew–Burke–Ernzerhof exchange-correlation functional. *J. Chem. Phys.* **110,** 5029-5036 (1999).
7. Dudarev S. L., Botton G. A., Savrasov S. Y., Humphreys C. J. & Sutton A. P. Electron-energy-loss spectra and the structural stability of nickel oxide: An LSDA+U study. *Phys. Rev. B* **57,** 1505-1509 (1998).
8. Merlo Ramirez J. M. & Carhart M. H. In-situ momentum dispersion in a single crystal of MoS_2. *Rev. Mex. Fis.* **70,** e050201 (2024).
