# Supplementary material for: Bypassing Schottky constraints via defect-mediated hydrogen transfer in hydrodechlorination
Source: Nat Commun. 2026 Jun 22;17:7861. doi: 10.1038/s41467-026-74751-0 (PMC13443187; doi:10.1038/s41467-026-74751-0)
Supplement: Supplementary file 2 [file 41467_2026_74751_MOESM2_ESM.pdf]

Description of Additional Supplementary Files

File Name: Supplementary Data 1
Description: Optimized DFT model coordinates supplied as separate Supplementary Data.
