# Supplementary material for: Bypassing Schottky constraints via defect-mediated hydrogen transfer in hydrodechlorination
Source: Nat Commun. 2026 Jun 22;17:7861. doi: 10.1038/s41467-026-74751-0 (PMC13443187; doi:10.1038/s41467-026-74751-0)
Supplement: Supplementary file 3 [file 41467_2026_74751_MOESM4_ESM.pdf]

nature portfolio

Peer Review File

Bypassing Schottky Constraints via Defect-Mediated Hydrogen Transfer in Hydrodechlorination

Corresponding Author: Professor Li-Zhi Huang

This file contains all reviewer reports in order by version, followed by all author rebuttals in order by version.

Version 0:

Reviewer comments:

Reviewer #1

(Remarks to the Author)
Summary:

In this work, the authors present an intriguing concept: exploiting a seemingly paradoxical phenomenon where the introduction of sulfur vacancies in MoS_2 widens its band gap (nominally increasing the electron transfer barrier) but simultaneously creates additional hybridized states that provide more channels for electron migration, ultimately enhancing charge transfer. This is a clever and interesting idea.

Major Concern:

However, while the fundamental mechanism is of interest, I cannot recommend this manuscript for publication in Nature Communications at this stage. The primary limitation is the extremely stringent reaction conditions. The key reaction requires a rigorously air-free environment, which renders it essentially impractical for any realistic, large-scale application. If the authors believe that the intrinsic defect engineering of MoS_{2-x} is the true key to the performance, then the role of $Fe(OH)_2$ is largely relegated to that of a active hydrogen (H*) generation site. To significantly enhance the practical relevance of this work, the authors should consider coupling their defect-engineered MoS_2 with other, more air-stable, materials that can supply active hydrogen. Demonstrating such a versatile and robust platform would substantially strengthen the case for publication in a high-impact venue.

Specific Comments and Questions:

Figure 1 (TEM): For the $MoS_2/Fe(OH)_2$ heterojunction shown in the TEM image, it would be beneficial to include EDS mapping to confirm the spatial distribution and intimate contact of the two phases.

XAFS Analysis. In the R-space XAS spectrum for Fe, the features for the $Fe(OH)_2/MoS_2$ sample appear to be proportionally scaled compared to the pristine $Fe(OH)_2$ sample. Could this scaling be attributed to a change in particle size or coordination number? Please provide clarification or fitting results to distinguish between these effects.

ESR Evidence (Figure 3c). The authors attribute the decrease in the DMPO-H ESR signal to more hydrogen being trapped on the surface. Is there direct in-situ spectroscopic evidence (e.g., in-situ Raman, IR, or XPS) to support the presence and nature of this trapped hydrogen state?

Defect Density and Intrinsic Activity. How does the concentration of sulfur vacancies quantitatively affect the intrinsic catalytic activity of MoS_2 for chloroform hydrodechlorination? Is there a direct correlation, or does an optimal vacancy density exist?

Impact on $Fe(OH)_2$ Function. Does the formation of the heterojunction with MoS_2 and the resulting internal electric field enhance the ability of $Fe(OH)_2$ to dissociate water and generate active hydrogen? If so, can this be experimentally quantified?

Interfacial Contact and H Migration.* At different $MoS_2/Fe(OH)_2$ mass ratios, is the physical and electronic contact between

the two phases consistent? Changes in the interface could alter the distance for H* spillover and migration, potentially affecting the overall kinetics. This should be considered when interpreting results from samples with different ratios.

Reviewer #2

(Remarks to the Author)
Remarks to the Author:
Overall, this study has relatively high academic value. The authors establish a fairly complete framework of “structure regulation–interfacial processes–reaction pathways–selective outcomes” based on a defect MoS2/Fe(OH)2 heterostructure, and achieve an improvement in the selectivity toward deep hydrodechlorination of chloroform under a controllable sulfur-vacancy gradient. This work does not merely remain at the level of an applied study on environmental pollutant removal, but advances the discussion toward a debatable and potentially transferable interfacial mechanism and theoretical interpretation. To address the apparent contradiction between the stabilizing effect of S vacancies on the key reductive reactive species (active hydrogen) and the vacancy-induced increase in the interfacial Schottky barrier, the authors propose a mechanism in which the system can still maintain effective interfacial electron supply and promote a higher contribution of the deep dechlorination pathway.
The evidence is organized in a solid manner. Multiple probes, including electrochemical responses, EPR with trapping and quenching experiments, kinetic isotope effects, and electrolyte perturbations, are used to cross-validate key inferences, rendering the conclusions reasonably traceable and to a certain extent verifiable. Overall, the work establishes a fairly close connection between environmental chemistry questions and materials interfacial mechanisms, reflecting a cross-disciplinary character with relatively sufficient theoretical support. The overall style is also largely consistent with this journal’s interest in “mechanistic elucidation and transferable concepts of broad significance.” After improvements in several expressions and in the presentation of controls, publication is recommended.

Major comments
1. Please add a short paragraph in the computational section of the Methods to clearly define how the Schottky barrier height (SBH) is specified and extracted. On this basis, please more clearly position the central contribution of this work: the defect heterostructure does not “eliminate the barrier,” but rather regulates the interfacial charge distribution and the electron-transfer mode through defect-related quasi-continuous energy levels and interfacial coupling, thereby mitigating excessive electron accumulation on the MoS2 side and improving the availability of electrons for subsequent reaction turnover. Accordingly, please refine the wording of “bypassing Schottky constraints” so that it is more accurately expressed as “alleviating SBH-induced charge accumulation and trapping effects / improving electron availability,” and avoid a possible misinterpretation that “a larger barrier is paradoxically easier to overcome.”
2. The definition of electron selectivity and the associated electron-counting scheme need to be fully transparent; otherwise, your central conclusion regarding “suppressing HER and redirecting electron flux to CH4” is not readily verifiable. Please provide a self-contained section (either in the main text or the SI) that explicitly states the calculation formula for electron selectivity and the electron-equivalent accounting/allocation, so that the analysis can be directly reproduced.
3. By what specific mechanism does Fe(OH)2 generate active hydrogen (H*)? In addition, the manuscript assumes that, at the same $Fe(OH)_2$ concentration, different MoS2/MoS2-with-defects samples exhibit approximately comparable hydrogen-evolution capability; however, this statement is not directly supported at present. It would be helpful if the authors could provide direct or indirect experimental evidence demonstrating that the H* generation capability is indeed comparable under identical Fe(OH)2 conditions.
4. The central conclusions of this manuscript rely on the premise that interfacial synergy in the heterojunction leads to performance enhancement and mechanistic advances. To more directly distinguish a genuine heterointerfacial effect from simple component additivity/physical mixing effects, it is recommended that the authors include a direct physical-mixture control prepared at the same composition ratio and dosage as the heterojunction sample.

Minor comments
1. References 13 and 36 are duplicates. Please verify and remove the duplicate entry or correct the numbering accordingly.
2. Reference 48 contains garbled/abnormal characters. Please check the original record and reformat the entry to ensure readability and traceability.
3. The journal formatting for Angew. Chem. Int. Ed. and Phys. Rev. B (Condensed Matter–related section/abbreviation) is incorrect. Please standardize these journal titles using the official abbreviations or the format required by this journal.
4. In the SI, several figure captions/descriptions are missing a space between the figure label/index (e.g., Figure Sx or (a)/(b)) and the accompanying text. Please format these consistently to improve readability.
5. In the SI, the pore size of the filter membrane is incorrectly written as 0.22 μM; it should be corrected to 0.22 μm.

Reviewer #3

(Remarks to the Author)
In this manuscript, the authors report a novel defect-engineered MoS2/Fe(OH)2 heterojunction catalyst for chloroform hydrodechlorination reaction. The long-standing kinetic trade-off between interfacial hydrogen stabilization and hydrogen mobility can be rationally overcome via defect-mediated electron-hydrogen coupling. Electronic structure analysis reveals that interfacial electric-field coupling drivers strong hybridization between vacancy-derived states and the MoS2 conduction band. This forms quasi-continuous defect-assisted tunneling channels that allow electrons to bypass the elevated Schotty barrier. This barrier-bypassing interfacial transport strategy of balancing reactive-hydrogen stabilization with mobility is interesting and innovative. Accordingly, in this context, the present study is suited for publication in ACS Catalysis. As whole, I would recommend this work for publication after the following issues are addressed.

Specific comments:
1.The “reactive hydrogen” should be replaced by “active hydrogen” to more precise expression.

2. The discussion in the manuscript regarding the high selectivity of the chloroform hydrodechlorination reaction and its correlation with various characterization results, especially the mechanism of the single-electron transfer (SET) transformation mediated by H*, is not sufficiently in-depth.

Version 1:

Reviewer comments:

Reviewer #2

(Remarks to the Author)
The revision is very deep and thorough, all my concerns are solved and carefully responded. The new finding in this paper should be a milestone in dechlorination study. So I think the paper is of high quality and meets the criteria to be published in this journal.
In addition, I noticed that reviewer#1 raised a concern regarding the practical applicability of the air-sensitive Fe(OH)2 system, alongside several technical queries regarding characterization and mechanistic interpretation. The authors have provided rigorous, satisfactory, and comprehensive responses to all points raised by reviewer#1. The revisions go beyond superficial text changes; they incorporate substantial new data—including HRTEM with EDS mapping, quantitative EXAFS fitting, and in situ IR spectroscopy—that directly address the reviewer's requests for evidence. Furthermore, the authors have contextualized the limitation regarding air-sensitivity honestly while articulating the fundamental geochemical and mechanistic value of the chosen model system. It can be concluded that the authors have fulfilled the revision requirements of reviewer #1 and that the manuscript now meets the high standards of evidence and interest expected for publication in Nature Communications.

Reviewer #3

(Remarks to the Author)
The revised manuscript answered most questions, so, I suggest publication.

Open Access This Peer Review File is licensed under a Creative Commons Attribution 4.0 International License, which permits use, sharing, adaptation, distribution and reproduction in any medium or format, as long as you give appropriate credit to the original author(s) and the source, provide a link to the Creative Commons license, and indicate if changes were made.
In cases where reviewers are anonymous, credit should be given to 'Anonymous Referee' and the source.
The images or other third party material in this Peer Review File are included in the article’s Creative Commons license, unless indicated otherwise in a credit line to the material. If material is not included in the article’s Creative Commons license and your intended use is not permitted by statutory regulation or exceeds the permitted use, you will need to obtain permission directly from the copyright holder.

To view a copy of this license, visit https://creativecommons.org/licenses/by/4.0/

REVIEWER COMMENTS

Reviewer #1 (Remarks to the Author):

Summary:

In this work, the authors present an intriguing concept: exploiting a seemingly paradoxical phenomenon where the introduction of sulfur vacancies in MoS_2 widens its band gap (nominally increasing the electron transfer barrier) but simultaneously creates additional hybridized states that provide more channels for electron migration, ultimately enhancing charge transfer. This is a clever and interesting idea.

Major Concern:

However, while the fundamental mechanism is of interest, I cannot recommend this manuscript for publication in Nature Communications at this stage. The primary limitation is the extremely stringent reaction conditions. The key reaction requires a rigorously air-free environment, which renders it essentially impractical for any realistic, large-scale application. If the authors believe that the intrinsic defect engineering of MoS_{2-x} is the true key to the performance, then the role of $Fe(OH)_2$ is largely relegated to that of an active hydrogen (H*) generation site. To significantly enhance the practical relevance of this work, the authors should consider coupling their defect-engineered MoS_2 with other, more air-stable, materials that can supply active hydrogen. Demonstrating such a versatile and robust platform would substantially strengthen the case for publication in a high-impact venue.

Specific Comments and Questions:

1. Figure 1 (TEM): For the $MoS_2/Fe(OH)_2$ heterojunction shown in the TEM image, it would be beneficial to include EDS mapping to confirm the spatial distribution and intimate contact of the two phases.

2. XAFS Analysis. In the R-space XAS spectrum for Fe, the features for the $Fe(OH)_2/MoS_2$ sample appear to be proportionally scaled compared to the pristine $Fe(OH)_2$ sample. Could this scaling be attributed to a change in particle size or coordination number? Please provide clarification or fitting results to distinguish between these effects.

3. ESR Evidence (Figure 3c). The authors attribute the decrease in the DMPO-H ESR signal to more hydrogen being trapped on the surface. Is there direct in-situ spectroscopic evidence (e.g., in-situ Raman, IR, or XPS) to support the presence and nature of this trapped hydrogen state?

4. Defect Density and Intrinsic Activity. How does the concentration of sulfur vacancies quantitatively affect the intrinsic catalytic activity of MoS_2 for chloroform hydrodechlorination? Is there a direct correlation, or does an optimal vacancy density exist?

5. Impact on $Fe(OH)_2$ Function. Does the formation of the heterojunction with MoS_2 and the resulting internal electric field enhance the ability of $Fe(OH)_2$ to dissociate water and generate active hydrogen? If so, can this be experimentally quantified?

6. Interfacial Contact and H Migration.* At different $MoS_2/Fe(OH)_2$ mass ratios, is the physical and electronic contact between the two phases consistent? Changes in the interface could alter the distance for H* spillover and migration, potentially affecting the overall kinetics. This should be considered when interpreting results from samples with different ratios.

Reviewer #2 (Remarks to the Author):

Overall, this study has relatively high academic value. The authors establish a fairly complete framework of "structure regulation–interfacial processes–reaction pathways–selective outcomes" based on a defect $MoS_2/Fe(OH)_2$ heterostructure, and achieve an improvement in the selectivity toward deep hydrodechlorination of chloroform under a controllable sulfur-vacancy gradient. This work does not merely remain at the level of an applied study on environmental pollutant removal, but advances the discussion toward a debatable and potentially transferable interfacial mechanism and theoretical interpretation. To address the apparent contradiction between the stabilizing effect of S vacancies on the key reductive reactive species (active hydrogen) and the vacancy-induced increase in the interfacial Schottky barrier, the authors propose a mechanism in which the system can still maintain effective interfacial electron supply and promote a higher contribution of the deep dechlorination pathway.
The evidence is organized in a solid manner. Multiple probes, including electrochemical responses, EPR with trapping and quenching experiments, kinetic isotope effects, and electrolyte perturbations, are used to cross-validate key inferences, rendering the conclusions reasonably traceable and to a certain extent verifiable. Overall, the work establishes a fairly close connection between environmental chemistry questions and materials interfacial mechanisms, reflecting a cross-disciplinary character with relatively sufficient theoretical support. The overall style is also largely consistent with this journal's interest in "mechanistic elucidation and transferable concepts of broad significance." After improvements in several expressions and in the presentation of controls, publication is recommended.

Major comments

1. Please add a short paragraph in the computational section of the Methods to clearly define how the Schottky barrier height (SBH) is specified and extracted. On this basis, please more clearly position the central contribution of this work: the defect heterostructure does not "eliminate the barrier," but rather regulates the interfacial charge distribution and the electron-transfer mode through defect-related quasi-continuous energy levels and interfacial coupling, thereby mitigating excessive electron accumulation on the MoS_2 side and improving the availability of electrons for subsequent reaction turnover. Accordingly, please refine the wording of "bypassing Schottky constraints" so that it is more accurately expressed as "alleviating SBH-induced charge accumulation and trapping effects / improving electron availability," and avoid a possible misinterpretation that "a larger barrier is paradoxically easier to overcome."
2. The definition of electron selectivity and the associated electron-counting scheme need to be fully transparent; otherwise, your central conclusion regarding "suppressing HER and redirecting electron flux to CH_4" is not readily verifiable. Please provide a self-contained section (either in the main text or the SI) that explicitly states the calculation formula for electron selectivity and the electron-equivalent accounting/allocation, so that the analysis can be directly reproduced.
3. By what specific mechanism does $Fe(OH)_2$ generate active hydrogen (H*)? In addition, the manuscript assumes that, at the same $Fe(OH)_2$ concentration, different MoS_2/MoS_2-with-defects samples exhibit approximately comparable hydrogen-evolution capability; however, this statement is not directly supported at present. It would be helpful if the authors could provide direct or indirect experimental evidence demonstrating that the H* generation capability is indeed comparable under identical $Fe(OH)_2$ conditions.
4. The central conclusions of this manuscript rely on the premise that interfacial synergy in the heterojunction leads to performance enhancement and mechanistic advances. To more directly distinguish a genuine heterointerfacial effect from simple component additivity/physical mixing effects, it is recommended that the authors include a direct physical-mixture control prepared at the same composition ratio and dosage as the heterojunction sample.

Minor comments
1. References 13 and 36 are duplicates. Please verify and remove the duplicate entry or correct the numbering accordingly.
2. Reference 48 contains garbled/abnormal characters. Please check the original record and reformat the entry to ensure readability and traceability.
3. The journal formatting for Angew. Chem. Int. Ed. and Phys. Rev. B (Condensed Matter–related section/abbreviation) is incorrect. Please standardize these journal titles using the official abbreviations or the format required by this journal.
4. In the SI, several figure captions/descriptions are missing a space between the figure label/index (e.g., Figure Sx or (a)/(b)) and the accompanying text. Please format these consistently to improve readability.

5. In the SI, the pore size of the filter membrane is incorrectly written as 0.22 μM; it should be corrected to 0.22 μm.

Reviewer #3 (Remarks to the Author):

In this manuscript, the authors report a novel defect-engineered $MoS_2/Fe(OH)_2$ heterojunction catalyst for chloroform hydrodechlorination reaction. The long-standing kinetic trade-off between interfacial hydrogen stabilization and hydrogen mobility can be rationally overcome via defect-mediated electron-hydrogen coupling. Electronic structure analysis reveals that interfacial electric-field coupling drives strong hybridization between vacancy-derived states and the MoS2 conduction band. This forms quasi-continuous defect-assisted tunneling channels that allow electrons to bypass the elevated Schottky barrier. This barrier-bypassing interfacial transport strategy of balancing reactive-hydrogen stabilization with mobility is interesting and innovative. Accordingly, in this context, the present study is suited for publication in Nature Communications. As a whole, I would recommend this work for publication after the following issues are addressed.

Specific comments:

1.The “reactive hydrogen” should be replaced by “active hydrogen” to more precise expression.

2. The discussion in the manuscript regarding the high selectivity of the chloroform hydrodechlorination reaction and its correlation with various characterization results, especially the mechanism of the single-electron transfer (SET) transformation mediated by H*, is not sufficiently in-depth.

Author Responses to Initial Comments:

Reviewers' Comments and Our Responses

We sincerely thank the Editor and the Reviewers for their careful evaluation of our manuscript and for the constructive and insightful comments. We have thoroughly revised the manuscript in response to all concerns raised. Substantial additions and clarifications have been incorporated, including new experimental results, extended analyses, and refined discussions to strengthen the mechanistic interpretation and practical relevance of the study. All revisions are highlighted in red in the revised manuscript, and the corresponding responses are provided point-by-point below. We hope that the revised version satisfactorily addresses the concerns and meets the standards for publication in Nature Communications.

To Reviewer #1

Comment:

Summary:

In this work, the authors present an intriguing concept: exploiting a seemingly paradoxical phenomenon where the introduction of sulfur vacancies in MoS_2 widens its band gap (nominally increasing the electron transfer barrier) but simultaneously creates additional hybridized states that provide more channels for electron migration, ultimately enhancing charge transfer. This is a clever and interesting idea.

Major Concern:

However, while the fundamental mechanism is of interest, I cannot recommend this manuscript for publication in Nature Communications at this stage. The primary limitation is the extremely stringent reaction conditions. The key reaction requires a rigorously air-free environment, which renders it essentially impractical for any realistic, large-scale application. If the authors believe that the intrinsic defect engineering of MoS_{2-x} is the true key to the performance, then the role of $Fe(OH)_2$ is

largely relegated to that of an active hydrogen (H*) generation site. To significantly enhance the practical relevance of this work, the authors should consider coupling their defect-engineered MoS_2 with other, more air-stable, materials that can supply active hydrogen. Demonstrating such a versatile and robust platform would substantially strengthen the case for publication in a high-impact venue.

Response: We sincerely thank the reviewer for raising this important point regarding the practical relevance of in situ H* generation under environmentally realistic (i.e., non-strictly anaerobic) conditions. We fully agree that a variety of materials and catalysts are capable of stably producing reactive hydrogen species for reductive transformations, including Ni-, Pd-, and Pt-based systems (e.g., *Environ. Sci. Technol., 2024, 58, 3079–3097; Nat. Mater., 2023, 22, 1022–1029; Nano Energ., 2017, 37, 74–80*). However, in the context of groundwater remediation, these systems may face important practical constraints. Catalysts relying on noble metals such as Pd or Pt inevitably involve limited natural abundance and high material cost, which restricts large-scale environmental deployment. While Ni-based systems offer a more economical alternative, large-scale introduction of additional transition metals into subsurface environments may raise concerns regarding long-term stability, secondary contamination, and regulatory acceptance.

In this study, we intentionally selected $Fe(OH)_2$ because it represents a geochemically relevant ferrous phase naturally present in subsurface environments, including corrosion products of iron materials. $Fe^{2+}/Fe(OH)_2$ is abundant, inexpensive, and environmentally benign, making it an intrinsically compatible electron donor for groundwater systems. Moreover, $Fe(OH)_2$ can be readily synthesized and exhibits a layered morphology (e.g., *Appl. Catal. B: Environ., 2023, 322, 122094*), which provides a structurally well-defined two-dimensional platform for constructing coherent heterointerfaces with MoS_2. This structural compatibility enables mechanistically clear interrogation of defect-mediated interfacial electron redistribution and hydrogen transfer processes, beyond conventional Schottky-type interface models.

In the revised manuscript, we have strengthened the Discussion section to explicitly acknowledge the reviewer's suggestion regarding the development of more oxygen-tolerant and structurally robust H*-generating partners. Identifying stable and air-resistant hydrogen-donating counterparts for defect-engineered MoS_{2-x} is indeed an important direction, and we are actively pursuing this line of investigation in our ongoing research.

[revision in page 16, line 433-435]

More broadly, this strategy of balancing active hydrogen stabilization with mobility establishes a general design principle for steering hydrogen-mediated pathways in complex heterogeneous catalytic systems. To further enhance the practical relevance of our findings, future work will focus on coupling defect-engineered MoS_2 with more air-stable hydrogen-generating materials, expanding the applicability of this approach for real-world, large-scale catalytic processes.

Comment: (1) Figure 1 (TEM): For the $MoS_2/Fe(OH)_2$ heterojunction shown in the TEM image, it would be beneficial to include EDS mapping to confirm the spatial distribution and intimate contact of the two phases.

Response: Thank for this helpful suggestion. In the revised manuscript, we have updated the HRTEM images in **Fig. 1d** to show a more representative interfacial region of the $MoS_2/Fe(OH)_2$ heterostructure. In addition, we have performed EDS mapping to directly verify the spatial distribution and interfacial integration of the two phases. As shown in the newly added **Fig. S13**, Fe and O signals ($Fe(OH)_2$) are distributed in regions in contact with Mo and S signals (MoS_2), supporting intimate interfacial contact and confirming the heterojunction architecture. The corresponding description has been added in the revised manuscript and Supplementary Information.

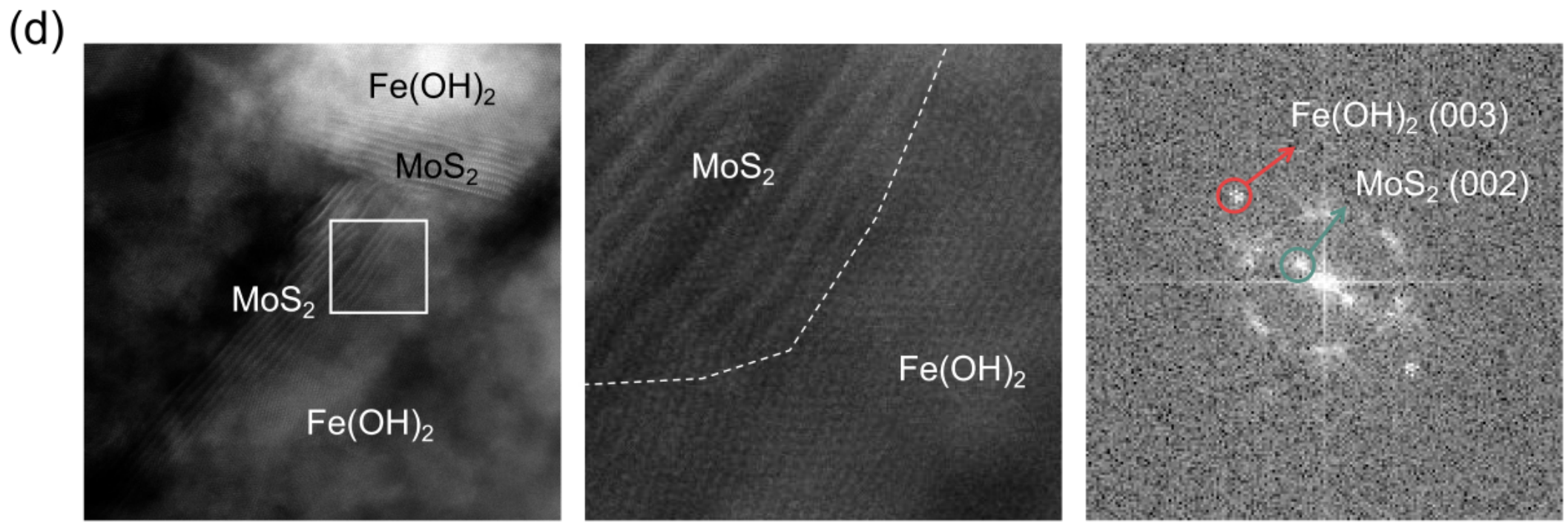

Figure 1(d). HRTEM image showing MoS_2 (002) and $Fe(OH)_2$ (003) in close contact at the interface.

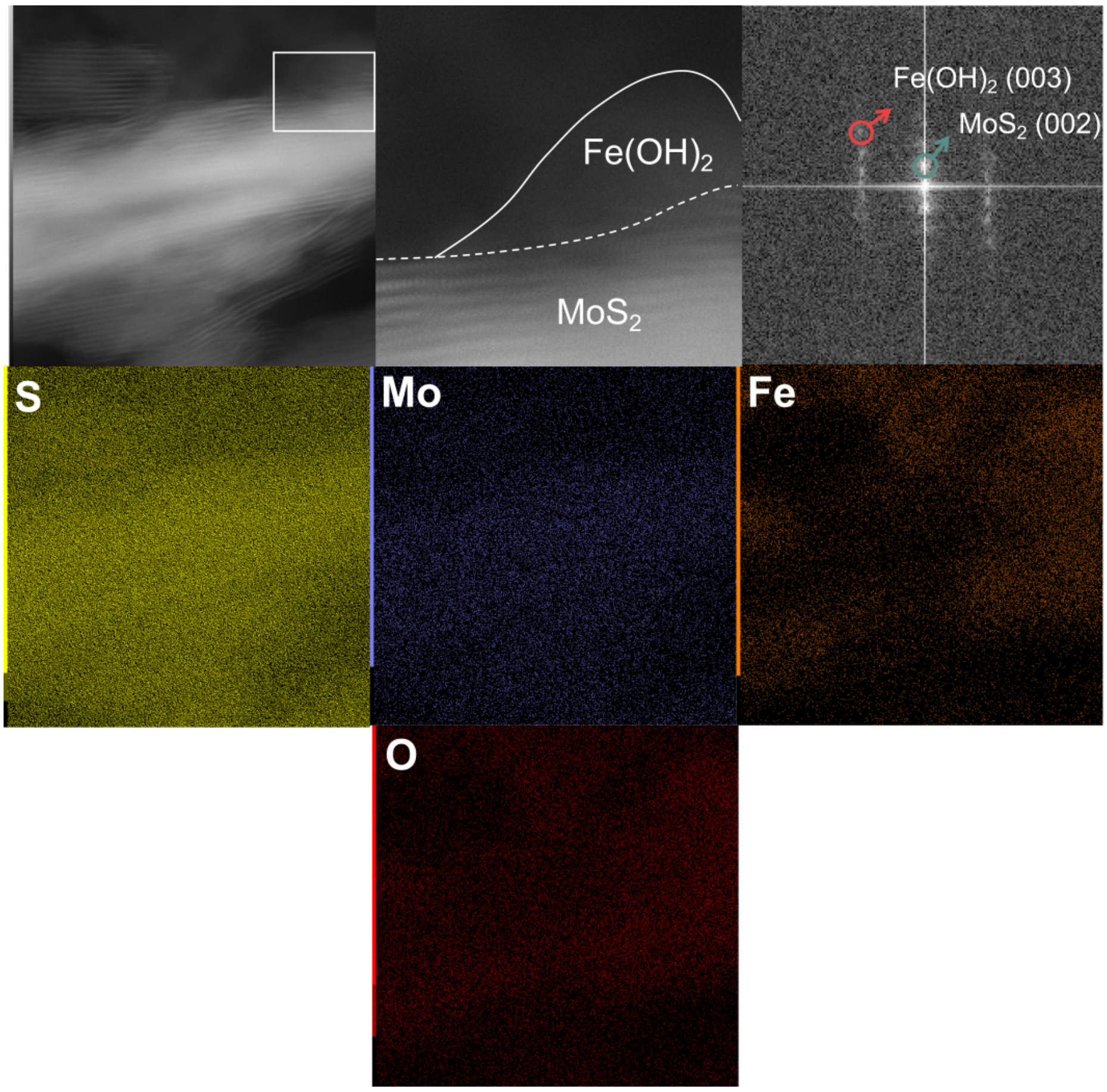

Figure S13. EDS mapping of the MoS_2/$Fe(OH)_2$ interface.

[revision in page 5, line 120-124]

High-resolution transmission electron microscopy (HRTEM) resolves lattice fringes corresponding to MoS_2 (002) and $Fe(OH)_2$ (003) in close contact at the interface (Fig. 1d), indicating intimate interfacial integration. This observation is further supported

by EDS mapping (Fig. S13), which shows Fe and O signals distributed in regions adjacent to and contacting the MoS_2 nanosheets (Mo and S))

Comment: (2) XAFS Analysis. In the R-space XAS spectrum for Fe, the features for the $Fe(OH)_2/MoS_2$ sample appear to be proportionally scaled compared to the pristine $Fe(OH)_2$ sample. Could this scaling be attributed to a change in particle size or coordination number? Please provide clarification or fitting results to distinguish between these effects.

Response: Thank you for this careful observation. We would like to clarify that the R-space XAS spectra shown in **Fig. 1h** are plotted directly from the processed EXAFS data without any manual intensity scaling between samples. The apparent amplitude difference therefore reflects intrinsic structural variations rather than graphical adjustment.

To further distinguish whether the observed amplitude change arises from particle size effects or coordination environment modification, we performed quantitative EXAFS fitting analysis for both pristine $Fe(OH)_2$ and $MoS_2/Fe(OH)_2$ samples. The fitting was conducted over an R-range of 0–6 Å using a standard Fe–O scattering model. The fitting results are summarized in **Table S2**.

For pristine $Fe(OH)_2$, the average Fe–O coordination number (CN) is fitted to be 6.72, with a bond distance of 2.01 Å and a Debye–Waller factor (σ^2) of 0.009 Å^2. In contrast, the $MoS_2/Fe(OH)_2$ sample exhibits a reduced coordination number of 5.26, a slightly increased Fe–O bond distance of 2.03 Å, and a σ^2 value of 0.013 Å^2.

These results indicate that the proportional reduction in R-space amplitude primarily originates from a decrease in the effective Fe–O coordination number. This reduced coordination is consistent with interfacial structural perturbation induced by coupling with MoS_2.

Table S2. Structural parameters obtained from EXAFS fitting in R space.

Sample	Path	CN	R(Å)	σ^2 (Å^2)	ΔE_0(eV)	R factor
$MoS_2/Fe(OH)_2$	Fe-O	5.26	2.03	0.013	-2.95	0.028
$Fe(OH)_2$	Fe-O	6.72	2.01	0.009	-4.72	0.024

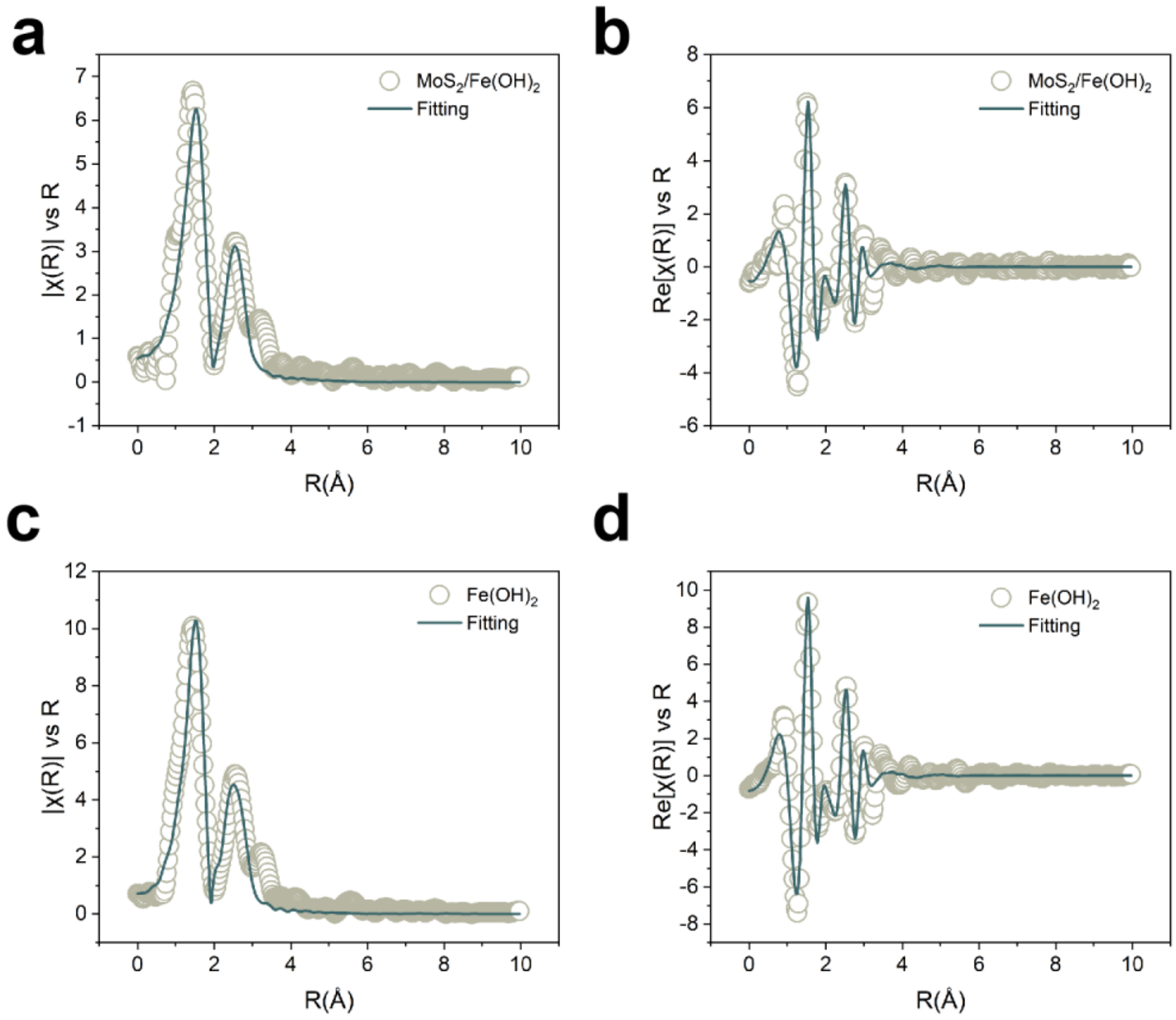

Figure S22. R-space EXAFS fitting results of $MoS_2/Fe(OH)_2$ and $Fe(OH)_2$. (a) |χ(R)| for $MoS_2/Fe(OH)_2$, (b) Corresponding real part, Reχ(R), for $MoS_2/Fe(OH)_2$, (c) |χ(R)| for /$Fe(OH)_2$ and (d) Corresponding real part, Reχ(R), for $Fe(OH)_2$.

[revision in page 6, line 141-145]

To elucidate the structural origin of this attenuation, we performed quantitative EXAFS fitting for pristine $Fe(OH)_2$ and $MoS_2/Fe(OH)_2$ (Fig. S22), with the fitting parameters summarized in Table S2. The fitting results indicate a reduced effective Fe-O coordination number (CN from 6.72 to 5.26), suggesting enhanced local structural under-coordination upon coupling with MoS_2. This phenomenon implied the inhibitory effect of the MoS_2 substrate on the independent bulk growth of $Fe(OH)_2$, prompting Fe species to participate in the construction of the heterogeneous interface in a more highly dispersed form.

Comment: (3) ESR Evidence (Figure 3c). The authors attribute the decrease in the DMPO-H ESR signal to more hydrogen being trapped on the surface. Is there direct in-situ spectroscopic evidence (e.g., in-situ Raman, IR, or XPS) to support the presence and nature of this trapped hydrogen state?

Response: Thank you for this insightful comment. To directly probe the nature of the trapped hydrogen suggested by ESR, we performed in-situ infrared spectroscopy under reaction conditions. Several distinct features were observed that support the stabilization of hydrogen at the $MoS_2/Fe(OH)_2$ interface:

1. ~1800 cm^{-1}: A peak corresponding to hydrated protons (H^+ in the hydrogen-bonded water network) gradually increases in intensity with higher MoS_2 loading. This feature reflects the role of solvated protons in facilitating effective proton transfer through H_3O^+, consistent with enhanced interfacial hydrogen flux and accelerated reaction kinetics.
2. ~2000 cm^{-1}: A new peak appears in the heterojunction samples but is absent in pure $Fe(OH)_2$. This feature is assignable to Mo-H vibrations, indicating the formation of hydrogen-bound Mo species in the presence of MoS_2. Since this signal is only observed in the heterojunction and becomes stronger with increasing MoS_2 loading, it suggests that H generated at $Fe(OH)_2$ is transferred to and stabilized on MoS_2. Combined with the defect-rich nature of MoS_{2-x}, these Mo-H species are likely associated with Mo sites adjacent to S vacancies, where the local electronic structure favors H trapping.
3. ~2500 cm^{-1}: A weaker absorption corresponding to S–H vibrations are also observed, suggesting that sulfur sites on MoS_2 provide additional, albeit less dominant, adsorption sites for H*.

These in-situ IR observations, together with the ESR results, provide direct spectroscopic evidence for the presence of surface-trapped hydrogen. Specifically, they demonstrate that H* is stabilized at Mo–H and S–H sites in the heterojunction and that hydrated protons in the interfacial water network facilitate directional proton transfer, supporting the proposed mechanism of effective H* migration and

utilization in deep hydrodechlorination.

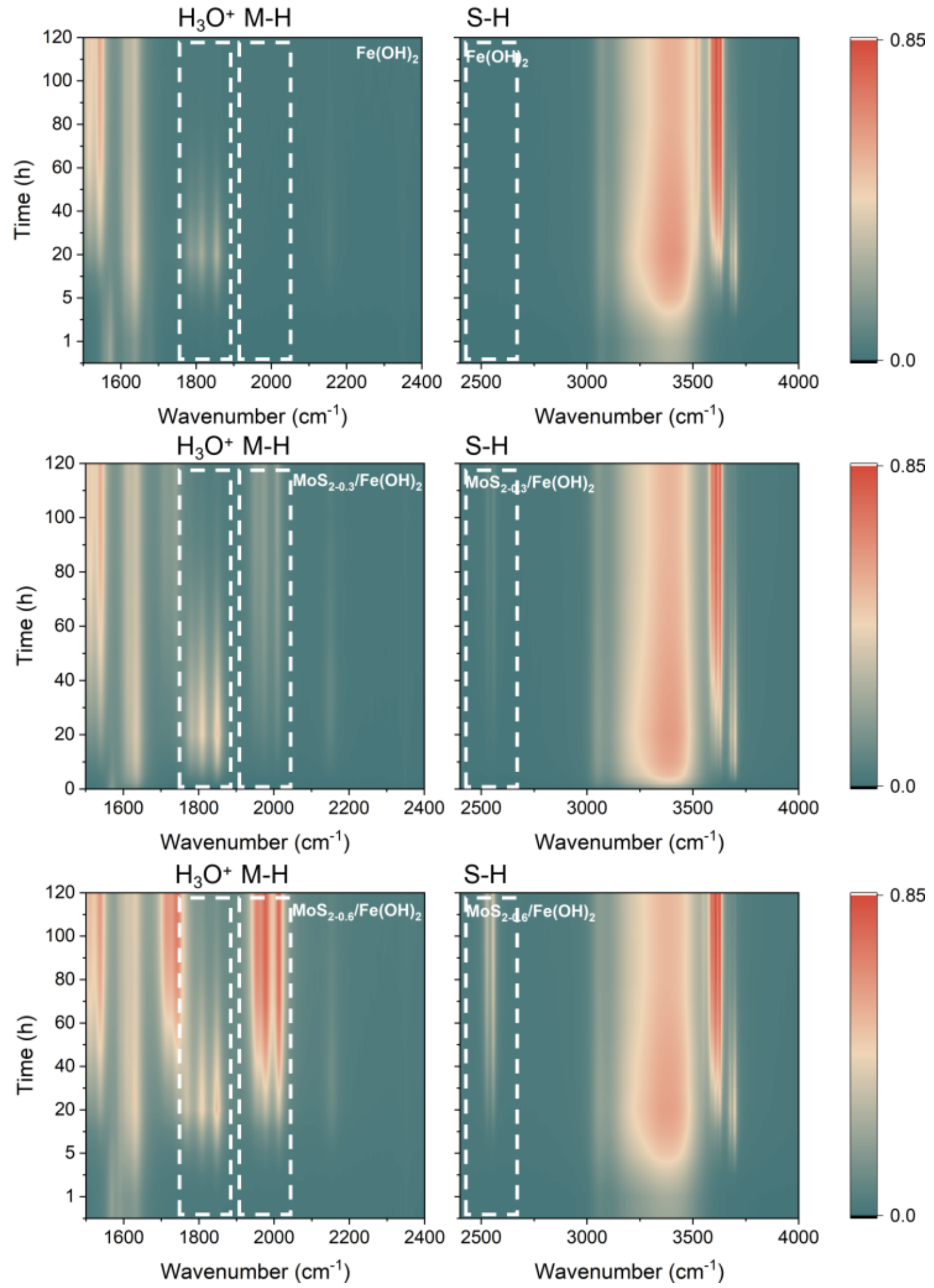

Figure 3(d). In situ IR spectra of $Fe(OH)_2$ and $MoS_2/Fe(OH)_2$ heterojunctions.

[revision in page 10-11, line 279-293]

To directly investigate the nature of the trapped hydrogen mentioned in the EPR results, we performed in situ infrared spectroscopy under reaction conditions (Fig. 3d). Several distinct features were observed in the experiment that support the stabilization of hydrogen at the $MoS_2/Fe(OH)_2$ interface. Specifically, at ~1800 cm^{-1}, we observed a peak corresponding to hydrated protons (H_3O^+), with its intensity increasing as the MoS_2 loading was increased. This indicates the role of solvated protons in facilitating proton transfer, thereby enhancing interfacial hydrogen flux and accelerating reaction kinetics. Additionally, at ~2000 cm^{-1}, a new peak appeared

in the heterojunction samples that was absent in pure $Fe(OH)_2$. This peak corresponds to metal-hydrogen (Mo-H) interactions, suggesting that H* is stabilized at Mo sites associated with S vacancies in MoS_2. The increasing intensity of this peak with MoS_2 loading further confirms that more H* is trapped at these defect sites. Simultaneously, at ~2500 cm^{-1}, we observed a weaker absorption corresponding to S-H vibrations, indicating that sulfur sites on MoS_2 provide additional adsorption sites for H*. In summary, these in situ infrared spectroscopy results, combined with the EPR findings, provide direct spectroscopic evidence for the presence of surface-trapped hydrogen, thus supporting the proposed mechanism of effective H* migration and utilization, particularly in deep hydrodechlorination reactions.

Comment: (4) Defect Density and Intrinsic Activity. How does the concentration of sulfur vacancies quantitatively affect the intrinsic catalytic activity of MoS_2 for chloroform hydrodechlorination? Is there a direct correlation, or does an optimal vacancy density exist?

Response: To quantitatively evaluate this relationship, we correlated the experimentally determined S-vacancy percentage (**Table S1**) with the pseudo-first-order rate constants compiled from **Tables S3-S7** (**Fig. S46**). Across the majority of experimental groups, the reaction rate increases monotonically with increasing vacancy density within the investigated range (0–9.05%). In a small subset of conditions, the rate increase becomes less pronounced when the vacancy density reaches ~6–9%, suggesting a tendency toward saturation rather than a strict linear scaling. Overall, these results support that sulfur-vacancy density serves as a positive descriptor for hydrodechlorination kinetics in the 0–9% window.

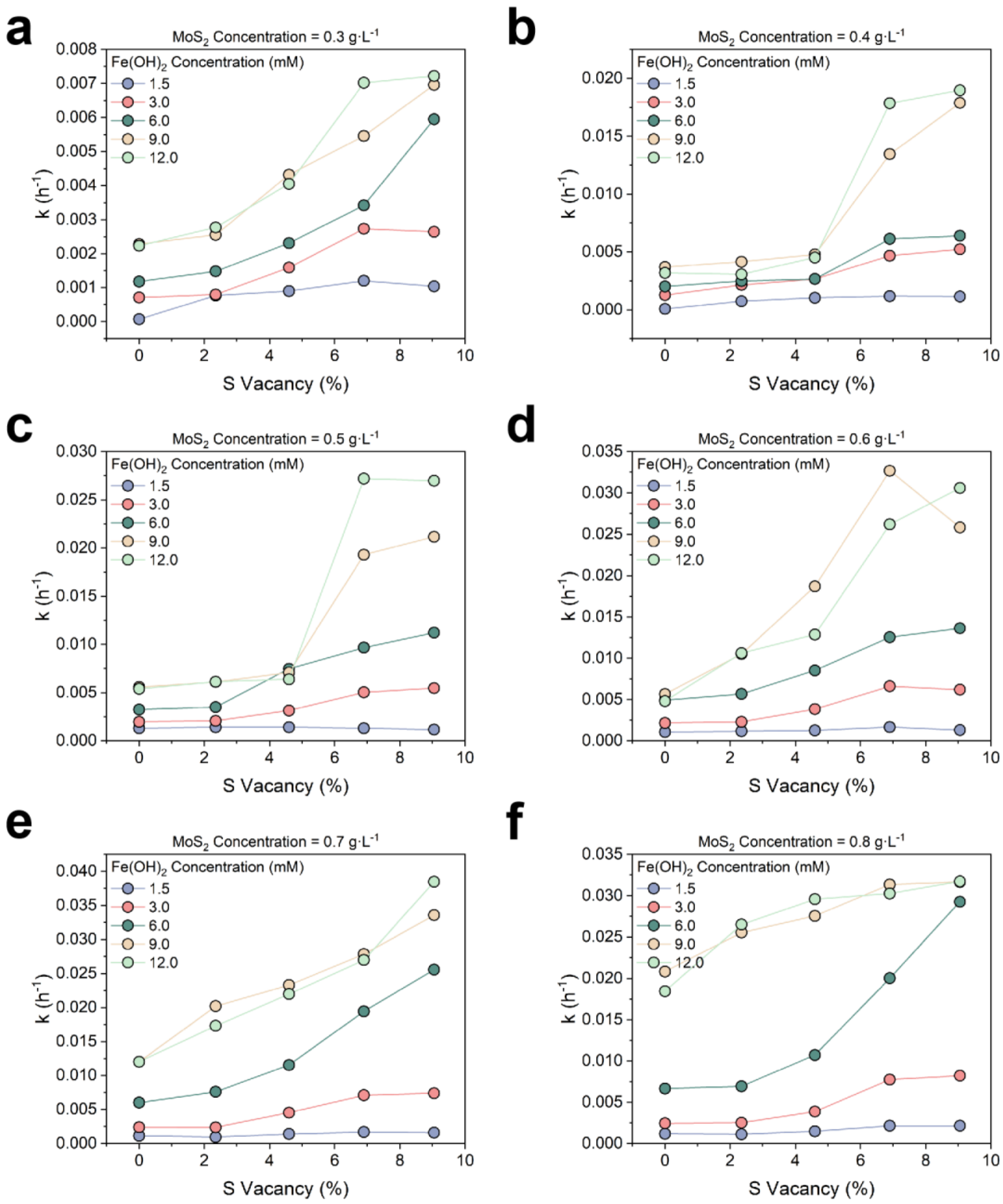

Figure S46. Correlation between sulfur-vacancy density and intrinsic pseudo-first-order rate constant (k_1) for chloroform degradation with different MoS_2 concentration. (a) 0.3 g·L^{-1}, (b) 0.4 g·L^{-1}, (c) 0.5 g·L^{-1}, (d) 0.6 g·L^{-1}, (e) 0.7 g·L^{-1} and (f) 0.8 g·L^{-1}.

[revision in page 13, line 342-347]

All experimental groups with the same sulfur-vacancy concentrations were plotted as a unified box plot, and the results (Fig. 3h) showed that increasing sulfur-vacancy density generally accelerates chloroform hydrodechlorination kinetics. The response plot of k_1 with sulfur-vacancy concentration under the same conditions (Tables S3-S7) further supported an overall monotonic dependence within the investigated vacancy range (0-9%), with a saturation-like tendency at higher vacancy densities in a small subset of experimental groups (Fig. S46).

Comment: (5) Impact on $Fe(OH)_2$ Function. Does the formation of the heterojunction with MoS_2 and the resulting internal electric field enhance the ability of $Fe(OH)_2$ to dissociate water and generate active hydrogen? If so, can this be experimentally quantified?

Response: Thank you for this insightful comment. To address this, we clarified in the revised manuscript (Results & Discussion, "Interfacial Geometry and S-Vacancies Govern the Fate of H*") how the formation of the $MoS_2/Fe(OH)_2$ heterojunction affects H* generation. H_2 evolution experiments across all concentration combinations (**Fig. S51**) show that H_2 production increases with increasing MoS_2 or $Fe(OH)_2$ loading, indicating that heterojunction coupling strength positively correlates with effective H* generation by $Fe(OH)_2$.

Additionally, our previous Mössbauer spectroscopy characterization has shown that the introduction of MoS_2 promotes electron transfer from $Fe(OH)_2$ to MoS_2, which alleviates the electronic asymmetry of Fe^{2+} in $Fe(OH)_2$. This results in a deep electronic reconstruction, enhancing the catalytic activity of $Fe(OH)_2$ and providing a more favorable structure for H* generation (**Figs. 1i and 1j**). Post-reaction Fe^{3+} measurements (**Figs. S44**) further reveal that, at fixed $Fe(OH)_2$ loading, higher MoS_2 loading leads to increased Fe^{3+}, suggesting that heterojunction formation may enhance the ability of $Fe(OH)_2$ to reduce protons.

Importantly, we emphasize that the intrinsic H* generation capacity of $Fe(OH)_2$ remains essentially constant when $Fe(OH)_2$ and MoS_2 loadings are fixed, as evidenced by the nearly unchanged post-reaction Fe^{3+} concentrations (**Figs. S43**). Therefore, the primary focus of this work is on how S-vacancies direct H* migration and pathway selectivity, rather than a significant enhancement of $Fe(OH)_2$ reactivity. These revisions are reflected in the revised text and figures.

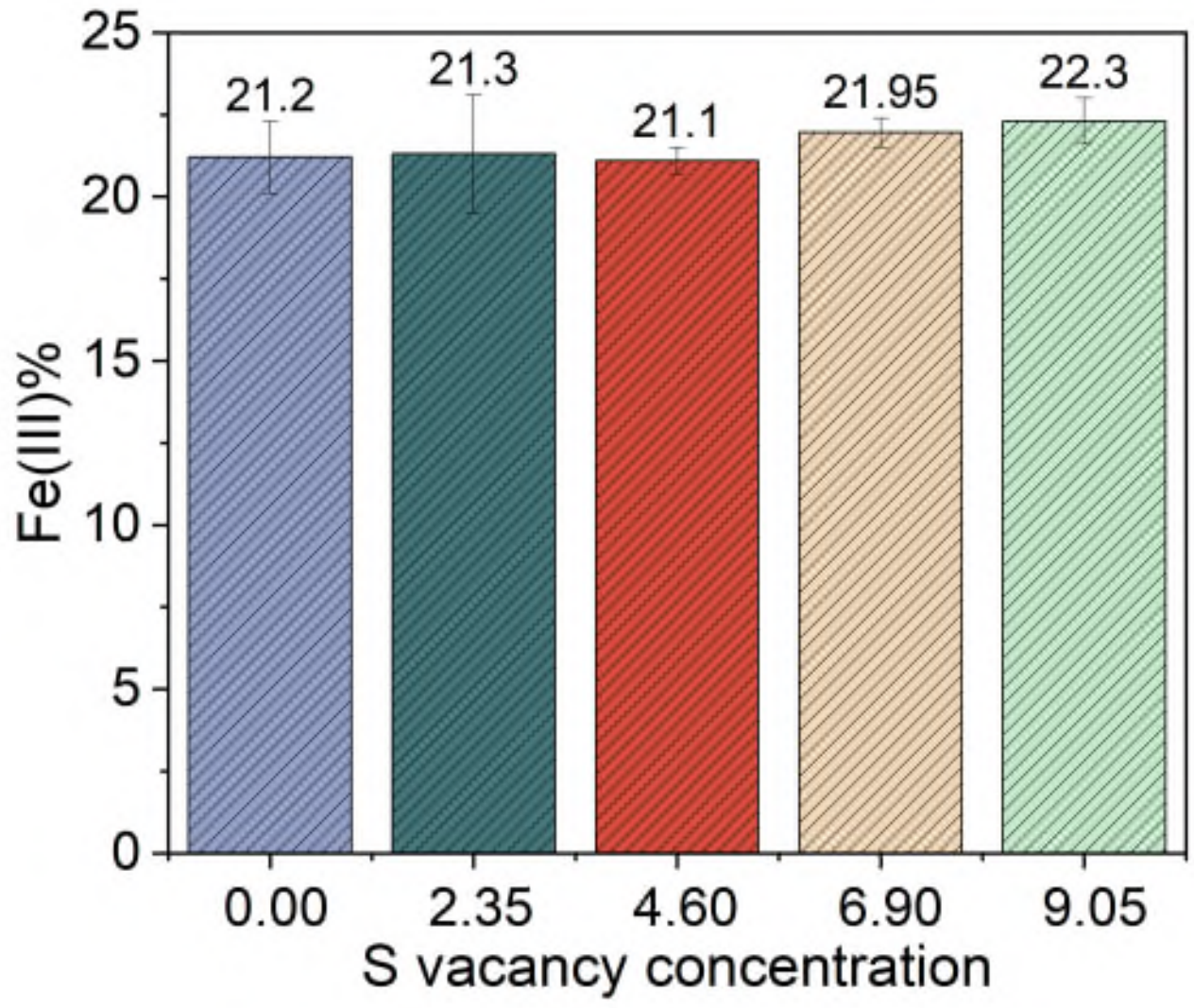

Figure S43. The concentration of Fe(III) after reaction under different initial S vacancy concentration.

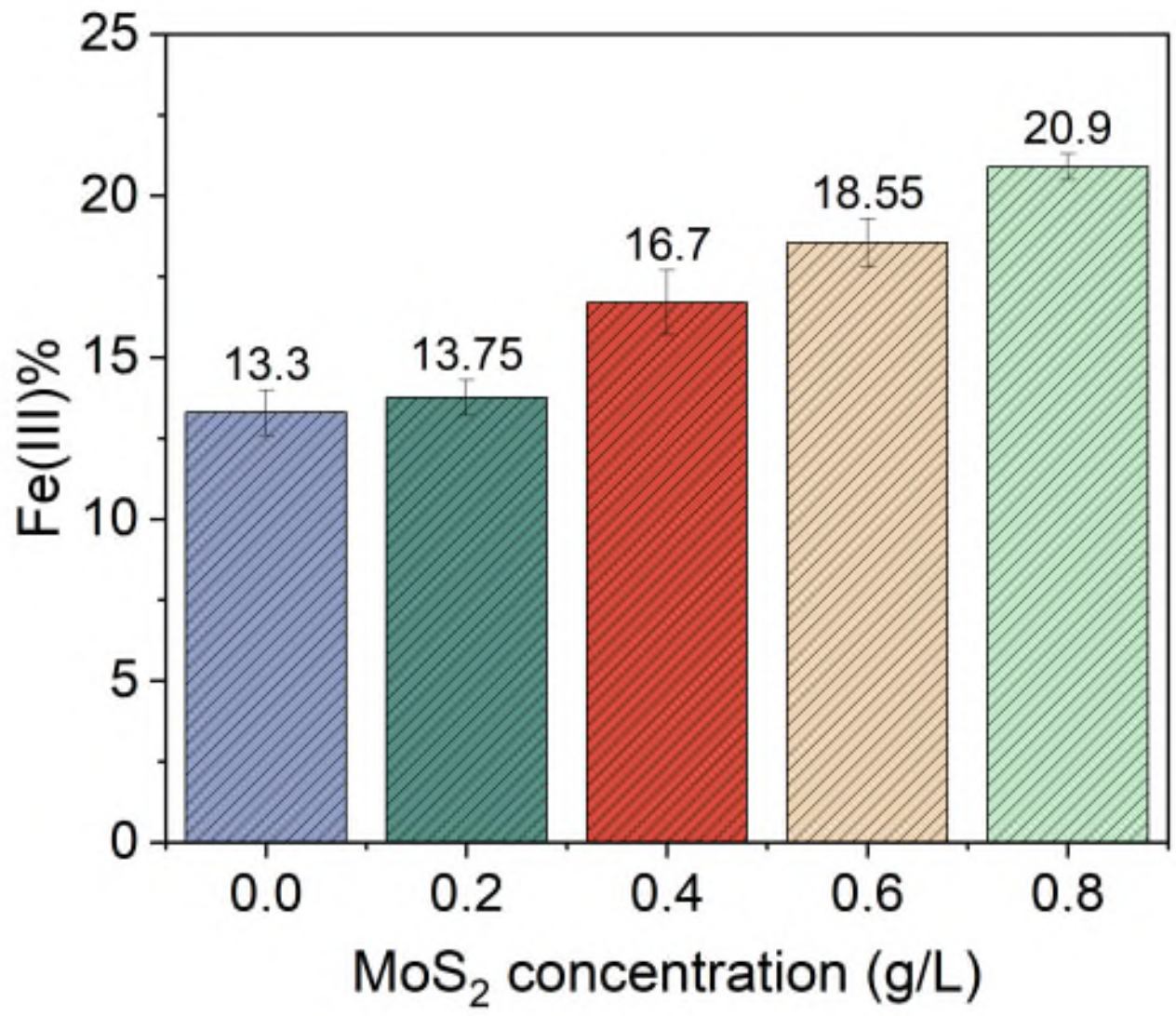

Figure S44. The concentration of Fe(III) after reaction under different initial MoS_2 concentrations.

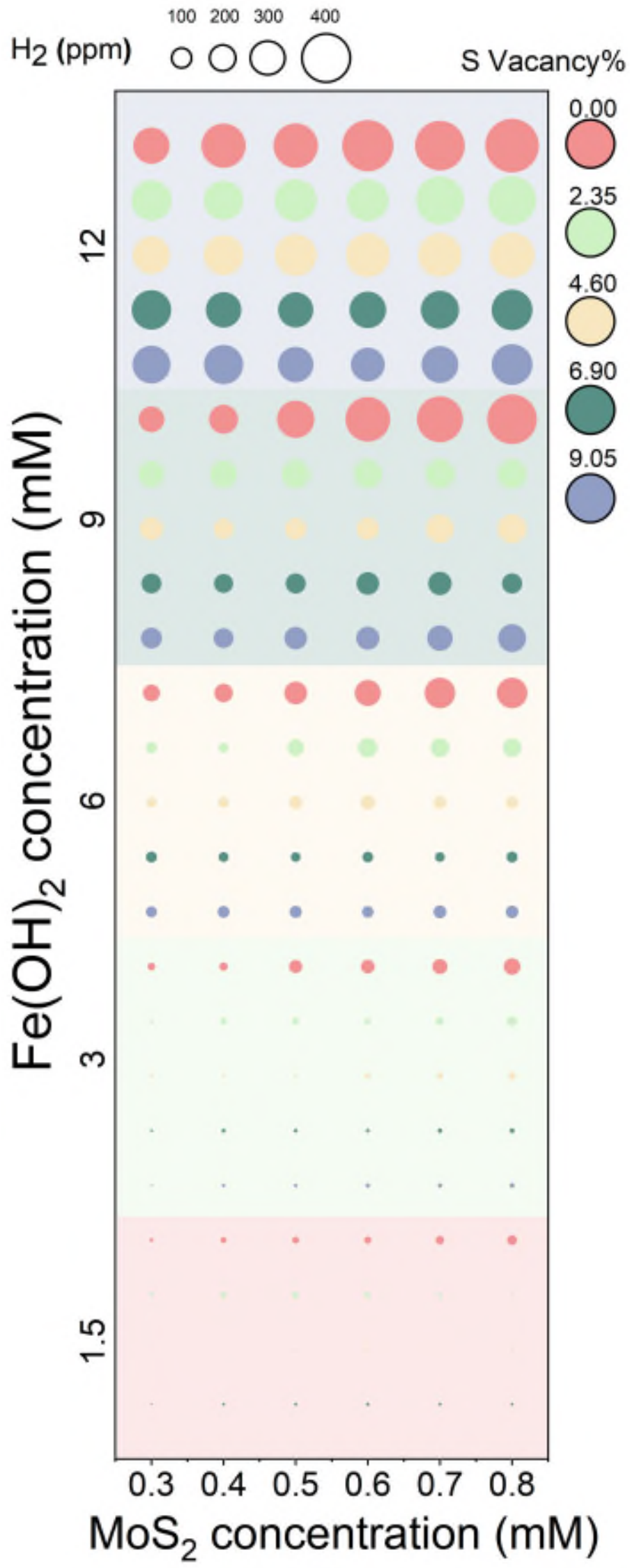

Figure S51. Hydrogen evolution experiments.

[revision in page 11, line 296-301]

Interfacial Geometry and S-Vacancies Govern the Fate of H*

1.Control experiments and heterojunction necessity

Control experiments showed that pristine $Fe(OH)_2$ alone exhibited negligible CF degradation activity (Fig. S47), while a physically mixed $Fe(OH)_2/MoS_2$ sample, prepared with identical composition and dosage as the heterojunction, displayed substantially suppressed CF conversion and almost no CH_4 formation (Fig. S50). These results establish that simple component addition is insufficient and that tight interfacial coupling is essential for enabling selective deep hydrodechlorination.

[revision in page 12, line 312-317 and 322-330]

2.Effect of MoS_2 loading and Fe^{3+} measurements

At low $MoS_2/Fe(OH)_2$ ratios, TEM images revealed that $Fe(OH)_2$ extensively covered the MoS_2 surfaces and exhibited severe aggregation (Fig. S15), reflecting degraded interfacial contact geometry. Under these conditions, H* remained largely confined within aggregated $Fe(OH)_2$ regions, where short H*-H* distances favored recombination to H_2 rather than transfer to MoS_2, leading to a shift in electron selectivity toward hydrogen evolution.

In contrast, at higher $MoS_2/Fe(OH)_2$ ratios, TEM images showed intimate contact between $Fe(OH)_2$ and MoS_2 surfaces, while MoS_2 retained ample exposed surface area enriched with coordinatively unsaturated sites (Fig. S14). Correspondingly, post-reaction Fe^{3+} concentrations increased with MoS_2 loading, suggesting that heterojunction formation enhances H* generation by $Fe(OH)_2$, likely due to an elevated Fe^{2+} reduction potential toward protons. Under this configuration, H* could be effectively captured and stabilized by vacancy-rich MoS_2. The increased population of stabilized H* facilitated interfacial hydrogen transfer, promoting deep hydrodechlorination and significantly boosting CH_4 electron selectivity while maintaining low DCM selectivity (Figs. 3f and 3g).

[revision in page 12, line 331-341]

3.Effect of S-vacancy density on H* generation

Having established the geometric requirements for efficient H* transfer, we next isolated the role of S-vacancy density at fixed $Fe(OH)_2$ loading (1.5 mM). When varying MoS_2 defect density, post-reaction Fe^{3+} concentrations remained nearly unchanged, indicating that the intrinsic H* generation capacity of $Fe(OH)_2$ is essentially constant. Interestingly, as shown in Fig. S51, at low S-vacancy concentrations (0%), H_2 production increased noticeably with MoS_2 loading, whereas at high S-vacancy densities (6-9%), H_2 evolution was largely insensitive to MoS_2 content. This suggests that the excess H* is effectively stabilized by MoS_2 S-vacancies. We attribute this behavior to two main factors: (i) at high MoS_2 loading,

$Fe(OH)_2$ aggregation is greatly reduced, mitigating confinement of H* within aggregated domains where short H*-H* distances favor recombination; (ii) Increasing the S-vacancy density alleviates electron accumulation at the interface, facilitates the subsequent migration of H*, and provides stabilized H* sites.

[revision in page 5 (Supplementary Information), line 135-139]

Text S8. The determination of Fe(II) concentration using a phenanthroline method

For the determination of total Fe(II), $MoS_2/Fe(OH)_2$ of molybdenite/Fe(III) were dissolved in 2 mL HCl (1 M) in the glove box. 100 μL of this solution was transferred to a volumetric flask containing 1.9 mL of phenanthroline solution and diluted 10 times by deionized water, and the total Fe(II) was then determined.

Comment: (6) Interfacial Contact and H* Migration. At different $MoS_2/Fe(OH)_2$ mass ratios, is the physical and electronic contact between the two phases consistent? Changes in the interface could alter the distance for H* spillover and migration, potentially affecting the overall kinetics. This should be considered when interpreting results from samples with different ratios.

Response: Thank you for highlighting this important point. We have explicitly addressed this in the revised manuscript (Results & Discussion, "Interfacial Geometry and S-Vacancies Govern the Fate of H*") by examining the effect of $MoS_2/Fe(OH)_2$ mass ratio on interfacial contact and H* migration.

TEM images show that at low $MoS_2/Fe(OH)_2$ ratios, $Fe(OH)_2$ extensively covers the MoS_2 surface and exhibits severe aggregation (**Fig. S15**), reflecting degradation of the interfacial contact geometry. Under these conditions, H* is largely confined within the aggregated $Fe(OH)_2$ domains, where the short H*-H* distances favor recombination to H_2 rather than transfer to MoS_2, leading to a shift in electron selectivity toward hydrogen evolution. In contrast, at higher $MoS_2/Fe(OH)_2$ ratios, TEM images reveal intimate contact between $Fe(OH)_2$ and MoS_2 surfaces, while MoS_2 retains abundant exposed surface area enriched with coordinatively unsaturated

sites (**Fig. S14**). This configuration enables H* to be efficiently captured and stabilized by the vacancy-rich MoS_2.

These structural variations directly influence H* spillover and migration distances, thereby affecting the overall reaction kinetics. The increased population of stabilized H* at higher MoS_2 loadings facilitates interfacial hydrogen transfer, promotes deep hydrodechlorination, and significantly enhances CH_4 electron selectivity while maintaining low DCM selectivity (**Figs. 3f and 3g**). Consequently, the observed differences in CF degradation rates and product distribution across samples with varying $MoS_2/Fe(OH)_2$ ratios can be rationalized based on interfacial contact and H* migration behavior, as now explicitly discussed in the revised manuscript.

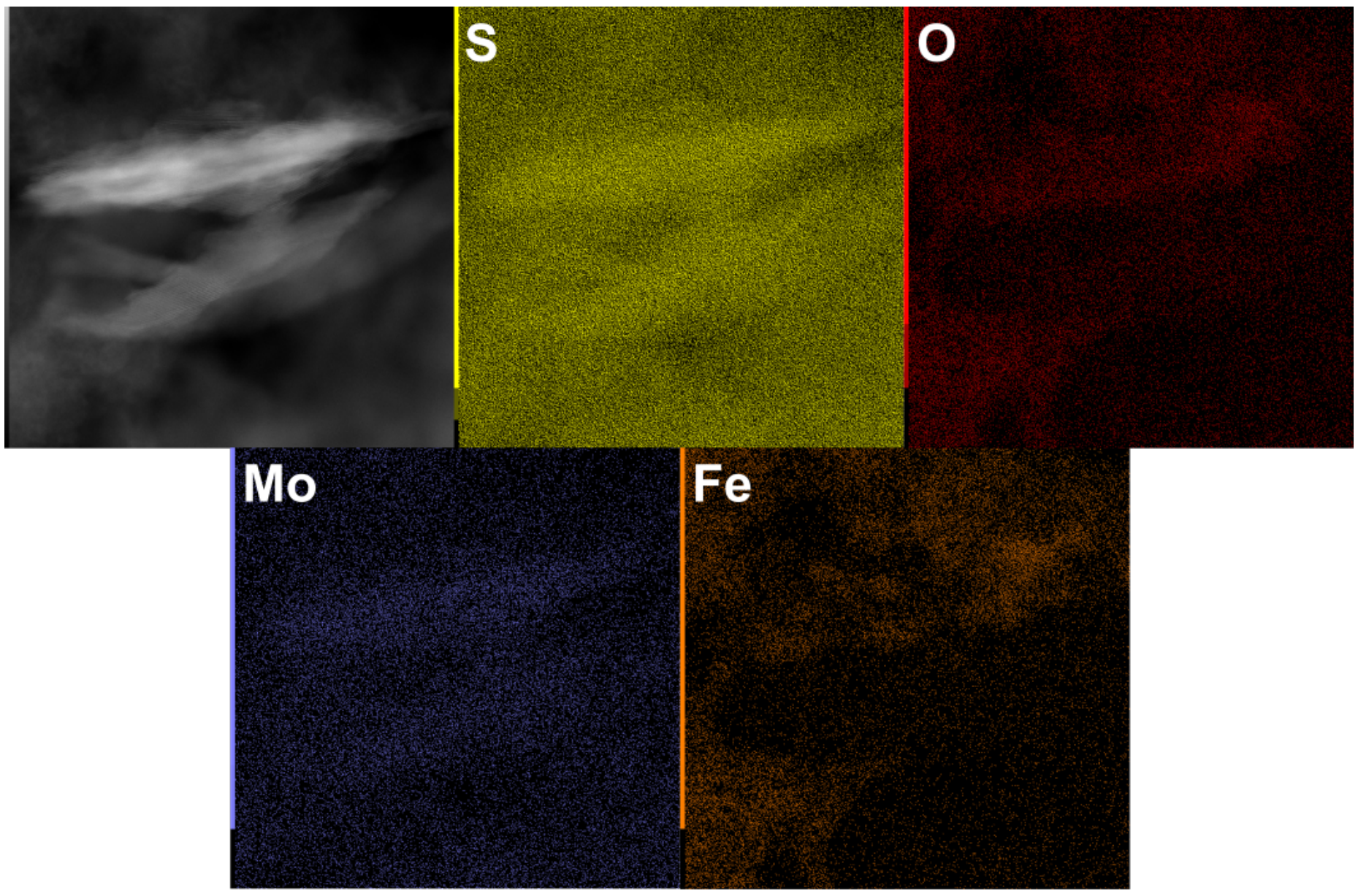

Figure S14. EDS mapping of $MoS_2/Fe(OH)_2$ (high MoS_2 loading).

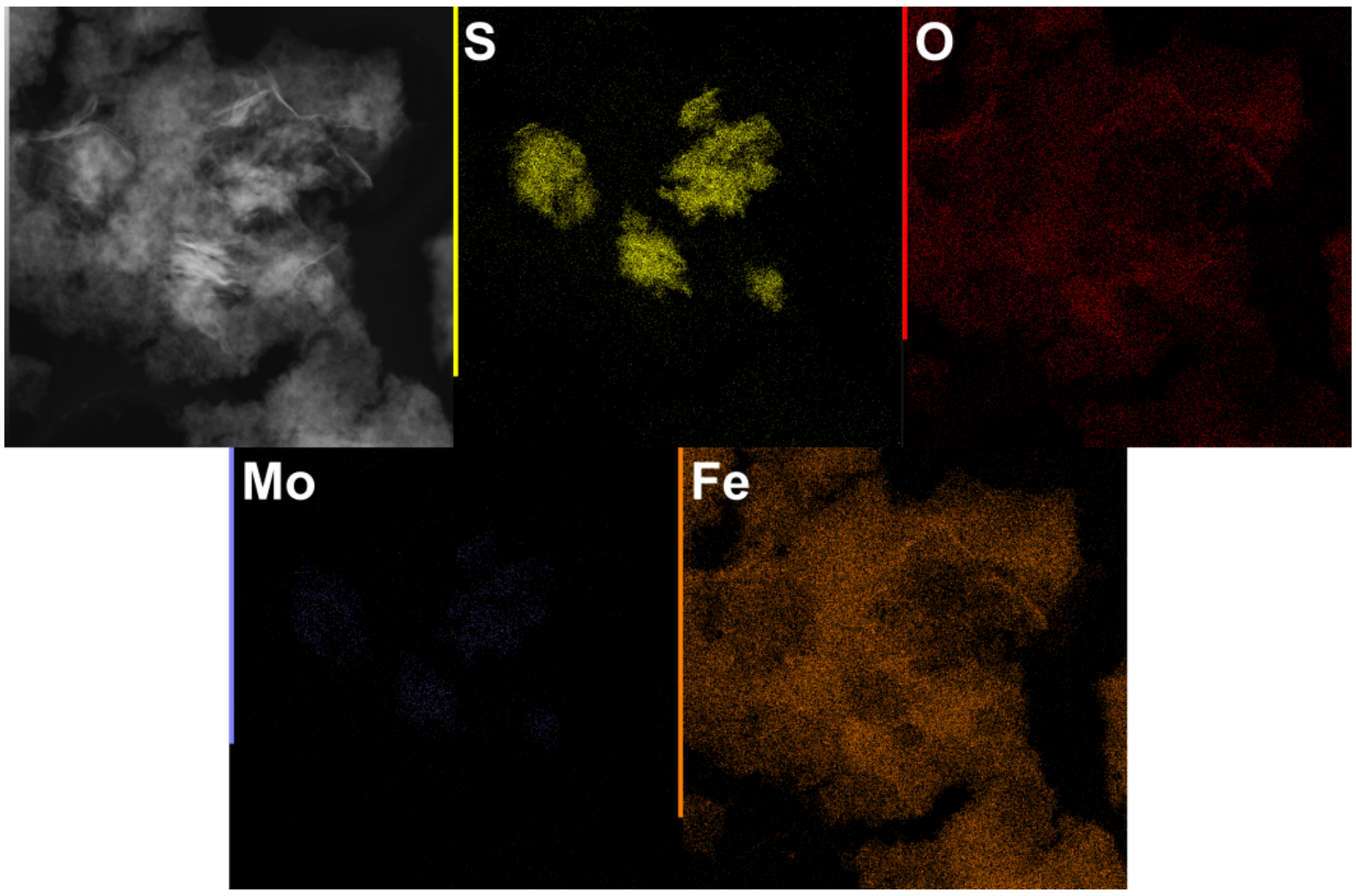

Figure S15. EDS mapping of $MoS_2/Fe(OH)_2$ (low MoS_2 loading).

[revision in page 12, line 312-330]

At low $MoS_2/Fe(OH)_2$ ratios, TEM images revealed that $Fe(OH)_2$ extensively covered the MoS_2 surfaces and exhibited severe aggregation (Fig. S15), reflecting degraded interfacial contact geometry. Under these conditions, H* remained largely confined within aggregated $Fe(OH)_2$ regions, where short H*-H* distances favored recombination to H_2 rather than transfer to MoS_2, leading to a shift in electron selectivity toward hydrogen evolution. When $Fe(OH)_2$ loading was further increased, H_2 electron selectivity remained above 90%, indicating that hydrogen evolution dominates and reduced interfacial coupling limits deep hydrodechlorination, even in the presence of abundant H*. In this scenario, due to the lack of effectively stabilized H*, deep hydrodechlorination was limited, and the products largely remained as the initial electron-transfer intermediates, dichloromethane (DCM), resulting in low methane (CH_4) yield (Figs. 3f and 3g).

In contrast, at higher $MoS_2/Fe(OH)_2$ ratios, TEM images showed intimate contact between $Fe(OH)_2$ and MoS_2 surfaces, while MoS_2 retained ample exposed surface area enriched with coordinatively unsaturated sites (Fig. S14). Correspondingly, post-reaction Fe^{3+} concentrations increased with MoS_2 loading, suggesting that heterojunction formation enhances H* generation by $Fe(OH)_2$, likely due to an elevated Fe^{2+} reduction potential toward protons. Under this configuration, H* could

be effectively captured and stabilized by vacancy-rich MoS_2. The increased population of stabilized H* facilitated interfacial hydrogen transfer, promoting deep hydrodechlorination and significantly boosting CH_4 electron selectivity while maintaining low DCM selectivity (Figs. 3f and 3g).

To Reviewer #2

Comment: Overall, this study has relatively high academic value. The authors establish a fairly complete framework of "structure regulation–interfacial processes–reaction pathways–selective outcomes" based on a defect $MoS_2/Fe(OH)_2$ heterostructure, and achieve an improvement in the selectivity toward deep hydrodechlorination of chloroform under a controllable sulfur-vacancy gradient. This work does not merely remain at the level of an applied study on environmental pollutant removal, but advances the discussion toward a debatable and potentially transferable interfacial mechanism and theoretical interpretation. To address the apparent contradiction between the stabilizing effect of S vacancies on the key reductive reactive species (active hydrogen) and the vacancy-induced increase in the interfacial Schottky barrier, the authors propose a mechanism in which the system can still maintain effective interfacial electron supply and promote a higher contribution of the deep dechlorination pathway.

The evidence is organized in a solid manner. Multiple probes, including electrochemical responses, EPR with trapping and quenching experiments, kinetic isotope effects, and electrolyte perturbations, are used to cross-validate key inferences, rendering the conclusions reasonably traceable and to a certain extent verifiable. Overall, the work establishes a fairly close connection between environmental chemistry questions and materials interfacial mechanisms, reflecting a cross-disciplinary character with relatively sufficient theoretical support. The overall style is also largely consistent with this journal's interest in "mechanistic elucidation and transferable concepts of broad significance." After improvements in several expressions and in the presentation of controls, publication is recommended.

Response: Thank you for reviewing our manuscript. We have revised the manuscript based on your insightful comments and suggestions. Please see our point-by-point responses below.

Comment: (1) Please add a short paragraph in the computational section of the Methods to clearly define how the Schottky barrier height (SBH) is specified and extracted. On this basis, please more clearly position the central contribution of this work: the defect heterostructure does not "eliminate the barrier," but rather regulates the interfacial charge distribution and the electron-transfer mode through defect-related quasi-continuous energy levels and interfacial coupling, thereby mitigating excessive electron accumulation on the MoS_2 side and improving the availability of electrons for subsequent reaction turnover. Accordingly, please refine the wording of "bypassing Schottky constraints" so that it is more accurately expressed as "alleviating SBH-induced charge accumulation and trapping effects / improving electron availability," and avoid a possible misinterpretation that "a larger barrier is paradoxically easier to overcome."

Response: We sincerely thank you for this thoughtful comment. Indeed, the original manuscript did not clearly point out the relationship between the defect-induced states, their effect on electron transfer mode, and the resulting SBH, which could lead to potential misinterpretation. As suggested, we have revised the manuscript to more accurately reflect the contribution of our work. Specifically, we have clarified that the defect heterostructure does not "eliminate" the Schottky barrier, but instead alleviates the SBH-induced charge accumulation and trapping effects, thereby improving the availability of electrons for subsequent reaction turnover.

In response to your request, we have added additional details regarding the SBH calculation in the Computational Methods section. SBH is determined by calculating the energy difference between the CBM of MoS_2 and the EF at the interface of $MoS_2/Fe(OH)_2$.

[revision in page 8, line 206-209]

Strong hybridization between vacancy-derived states and the MoS_2 conduction band led to the emergence of delocalized electronic features near the Fermi level, which was consistent with facilitated electron delivery to surface-active sites despite the presence of an elevated interfacial SBH (Fig. 2f). This deep coupling reduces the energy barrier effects caused by SBH, preventing electron trapping and enhancing electron availability for reaction turnover. It also alleviates excessive charge accumulation at the interface, facilitating H* turnover. Together, these electronic-structure trends provided a basis for the experimentally observed enhancement in interfacial H* utilization and hydrogen-transfer reactivity discussed below.

[revision in page 16-17, line 462-469]

Computational Methods

Density functional theory (DFT) calculations were performed to analyze band alignment, defect-induced states, and interfacial charge redistribution. Vacancy concentrations were systematically varied to examine their impact on conduction-band position, SBH, and electron transport pathways. The SBH was determined by calculating the energy difference between the CBM of MoS_2 and the Fermi level (E_F) at the interface of $MoS_2/Fe(OH)_2$. Charge density, projected density of states, and adsorption energetics were evaluated to correlate electronic reconstruction with hydrogen stabilization and mobility. Computational details and model parameters are provided in the Supporting Information.

Comment: (2) The definition of electron selectivity and the associated electron-counting scheme need to be fully transparent; otherwise, your central conclusion regarding "suppressing HER and redirecting electron flux to CH_4" is not readily verifiable. Please provide a self-contained section (either in the main text or the SI) that explicitly states the calculation formula for electron selectivity and the

electron-equivalent accounting/allocation, so that the analysis can be directly reproduced.

Response: We sincerely thank you for raising this important point. We agree that a transparent definition of electron selectivity and a clear electron-counting scheme are essential for the reproducibility of the analysis. To address this concern, we have added a self-contained section in the Supporting Information (**Supporting Information Text 7**) that explicitly defines the electron selectivity and the associated electron-equivalent accounting.

[revision in page 4 (Supplementary Information), line 125-133]

Text S7. Calculation of Electron Selectivity

In this study, electron selectivity is defined as the fraction of the total electrons directed towards each product, which is calculated based on the electron consumption of each product (chloroform to methane, chloroform to dichloromethane, and hydrogen). The electron selectivity for each product is then calculated using the following formula:

$$S_i = \frac{Y_i \times n_i}{\sum_i (Y_i \times n_i)}$$

Where:

S_i= Electron selectivity for Product i;

Y_i= Yield of Product i;

n_i= Electrons consumed per molecule of Product i, $n_{methane}$ = 6, $n_{dichloromethane}$ = 2, $n_{hydrogen}$ = 2.

Comment: (3) By what specific mechanism does $Fe(OH)_2$ generate active hydrogen (H*)? In addition, the manuscript assumes that, at the same $Fe(OH)_2$ concentration, different MoS_2/MoS_2-with-defects samples exhibit approximately comparable hydrogen-evolution capability; however, this statement is not directly supported at present. It would be helpful if the authors could provide direct or indirect experimental evidence demonstrating that the H* generation capability is indeed comparable under identical $Fe(OH)_2$ conditions.

Response: Thank you for this insightful comment. H* is generated on $Fe(OH)_2$ primarily through the reduction of protons by Fe^{2+}, during which a portion of Fe^{2+} is oxidized to Fe^{3+}. In addition, interfacial charge transfer within the $MoS_2/Fe(OH)_2$ heterojunction may further facilitate H* generation.

To demonstrate that the intrinsic H* generation capability of $Fe(OH)_2$ is comparable across different MoS_2 or S-vacancy samples at the same $Fe(OH)_2$ loading, we performed H_2 evolution experiments and measured post-reaction Fe^{3+} concentrations (**Figs. S44, S43 and S51**). These results show that, for a fixed $Fe(OH)_2$ concentration, the total H* generation is nearly identical among the different samples. Consequently, the observed differences in CF degradation kinetics and product selectivity are primarily attributed to the fate of H*—whether it recombines to form H_2 or is captured and stabilized by vacancy-rich MoS_2—rather than variations in the intrinsic H* generation activity of $Fe(OH)_2$.

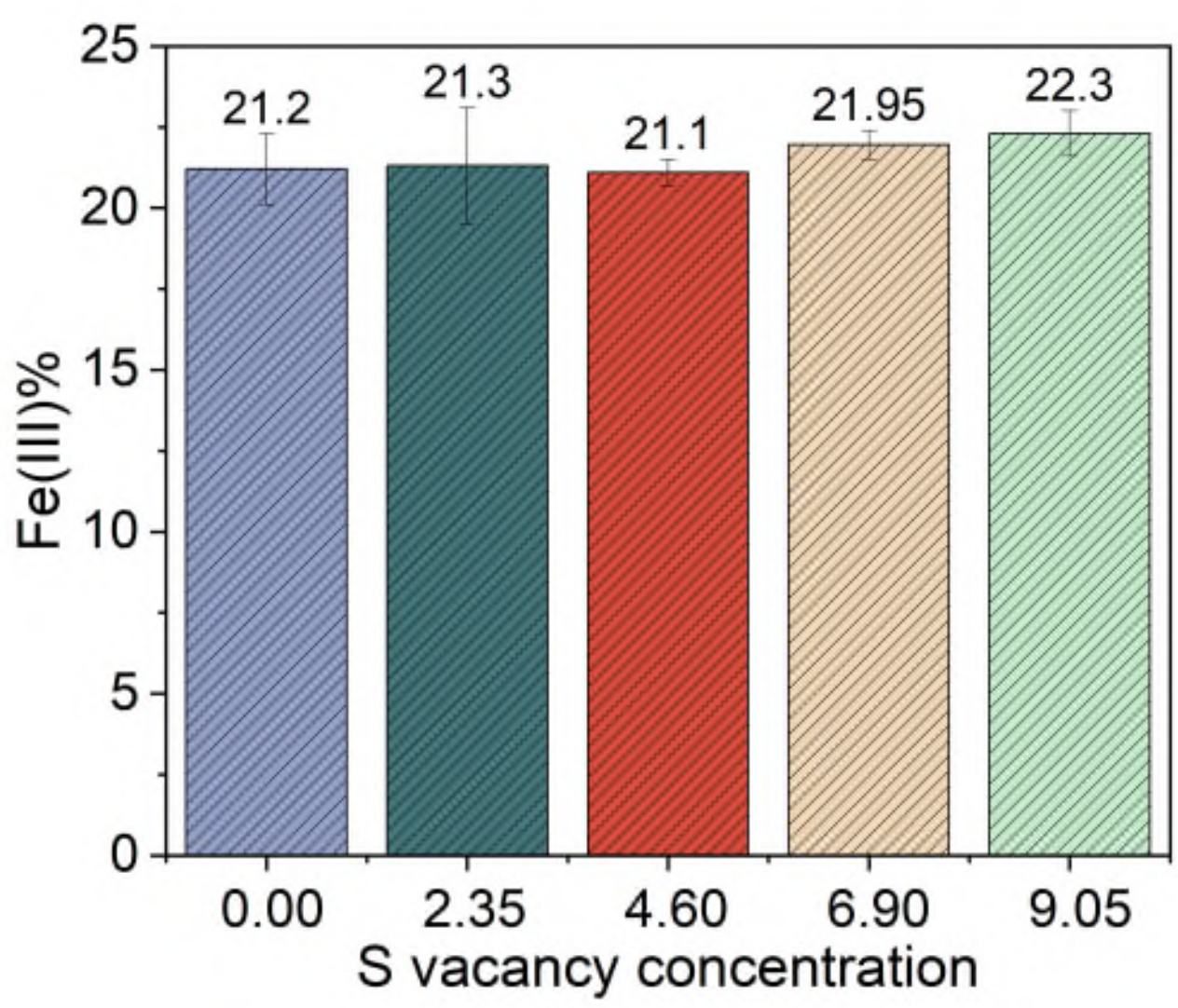

Figure S43. The concentration of Fe(III) after reaction under different initial S vacancy concentration.

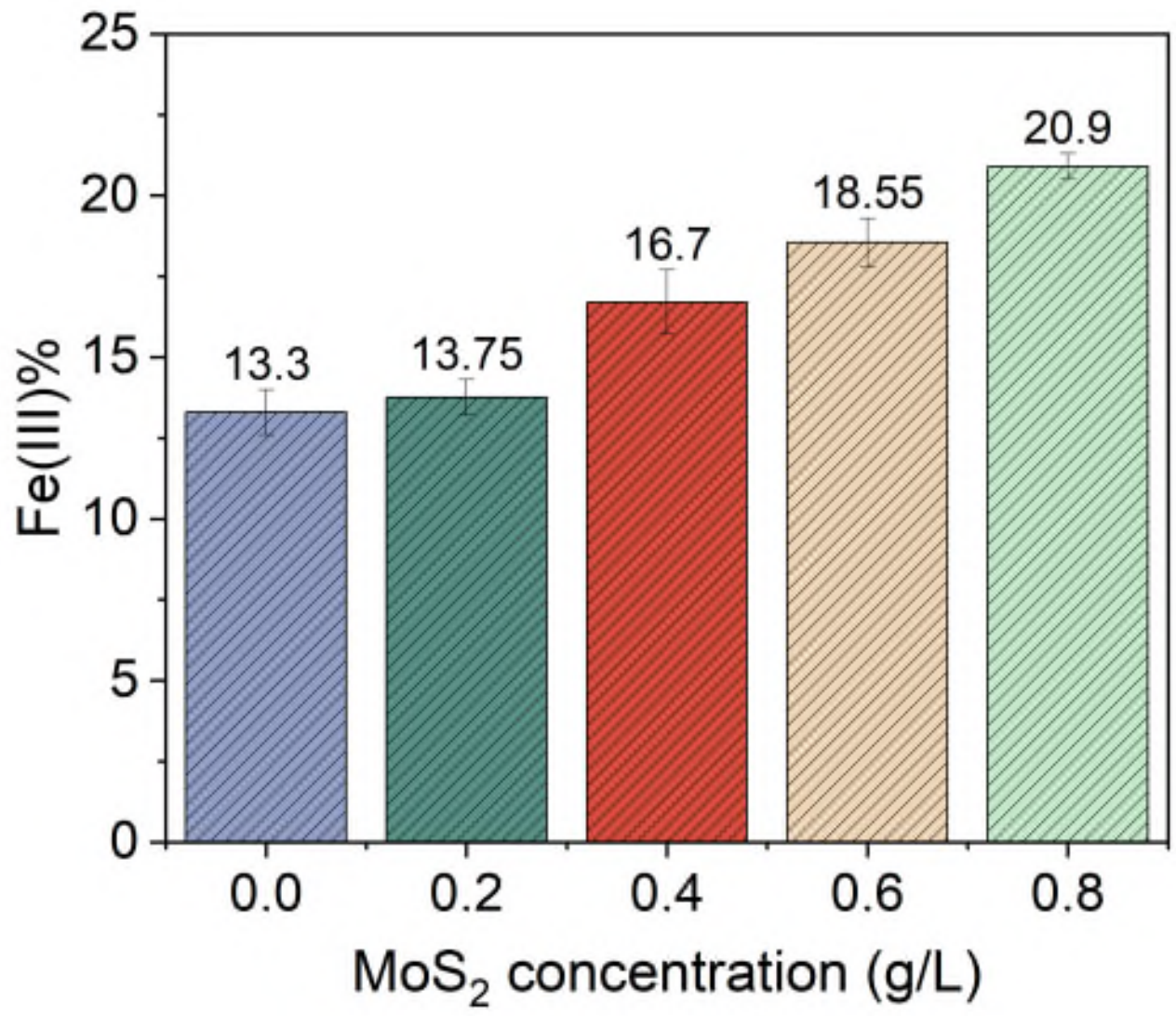

Figure S44. The concentration of Fe(III) after reaction under different initial MoS_2 concentration

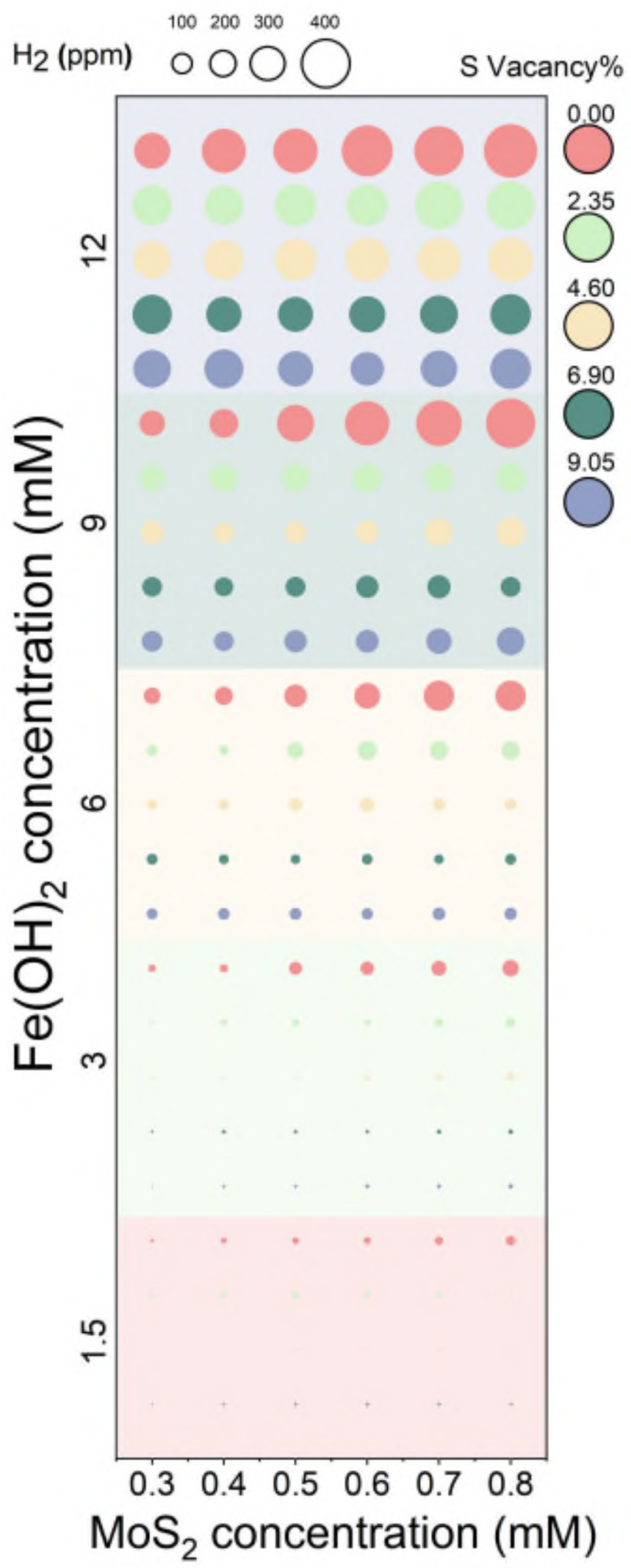

Figure S51. Hydrogen evolution experiments.

[revision in page 12, line 312-341]

At low $MoS_2/Fe(OH)_2$ ratios, TEM images revealed that $Fe(OH)_2$ extensively covered the MoS_2 surfaces and exhibited severe aggregation (Fig. S15), reflecting degraded interfacial contact geometry. Under these conditions, H* remained largely confined within aggregated $Fe(OH)_2$ regions, where short H*-H* distances favored recombination to H_2 rather than transfer to MoS_2, leading to a shift in electron selectivity toward hydrogen evolution. When $Fe(OH)_2$ loading was further increased, H_2 electron selectivity remained above 90%, indicating that hydrogen evolution dominates and reduced interfacial coupling limits deep hydrodechlorination, even in the presence of abundant H*. In this scenario, due to the lack of effectively stabilized H*, deep hydrodechlorination was limited, and the products largely remained as the initial electron-transfer intermediates, dichloromethane (DCM), resulting in low methane (CH_4) yield (Figs. 3f and 3g).

In contrast, at higher $MoS_2/Fe(OH)_2$ ratios, TEM images showed intimate contact between $Fe(OH)_2$ and MoS_2 surfaces, while MoS_2 retained ample exposed surface area enriched with coordinatively unsaturated sites (Fig. S14). Correspondingly, post-reaction Fe^{3+} concentrations increased with MoS_2 loading, suggesting that heterojunction formation enhances H* generation by $Fe(OH)_2$, likely due to an elevated Fe^{2+} reduction potential toward protons. Under this configuration, H* could be effectively captured and stabilized by vacancy-rich MoS_2. The increased population of stabilized H* facilitates interfacial hydrogen transfer, promoting deep hydrodechlorination and significantly boosting CH_4 electron selectivity while maintaining low DCM selectivity (Figs. 3f and 3g).

Having established the geometric requirements for efficient H* transfer, we next isolated the role of S-vacancy density at fixed $Fe(OH)_2$ loading (1.5 mM). When varying MoS_2 defect density, post-reaction Fe^{3+} concentrations remained nearly unchanged, indicating that the intrinsic H* generation capacity of $Fe(OH)_2$ is essentially constant. Interestingly, as shown in Fig. S51, at low S-vacancy concentrations (0%), H_2 production increased noticeably with MoS_2 loading, whereas

at high S-vacancy densities (6-9%), H_2 evolution was largely insensitive to MoS_2 content. This suggests that the excess H* is effectively stabilized by MoS_2 S-vacancies. We attribute this behavior to two main factors: (i) at high MoS_2 loading, $Fe(OH)_2$ aggregation is greatly reduced, mitigating confinement of H* within aggregated domains where short H*-H* distances favor recombination; (ii) Increasing the S-vacancy density alleviates electron accumulation at the interface, facilitates the subsequent migration of H*, and provides stabilized H* sites.

Comment: (4) The central conclusions of this manuscript rely on the premise that interfacial synergy in the heterojunction leads to performance enhancement and mechanistic advances. To more directly distinguish a genuine heterointerfacial effect from simple component additivity/physical mixing effects, it is recommended that the authors include a direct physical-mixture control prepared at the same composition ratio and dosage as the heterojunction sample.

Response: Thank you for the thoughtful suggestion. To more directly distinguish the genuine heterointerfacial effect from simple component additivity or physical mixing effects, we have prepared a physical-mixture control catalyst. Specifically, $Fe(OH)_2$ was dried overnight in an argon glove box, and then collected as a white powder. After grinding, it was physically mixed with MoS_2 at the same composition ratio and dosage as the heterojunction sample in the glove box. The results show that the CF degradation efficiency was significantly lower for the physical mixture, with almost no CH_4 product detected, suggesting that the interfacial synergy in the heterojunction plays a crucial role in enhancing the reaction performance. These results are presented in **Fig. S50**.

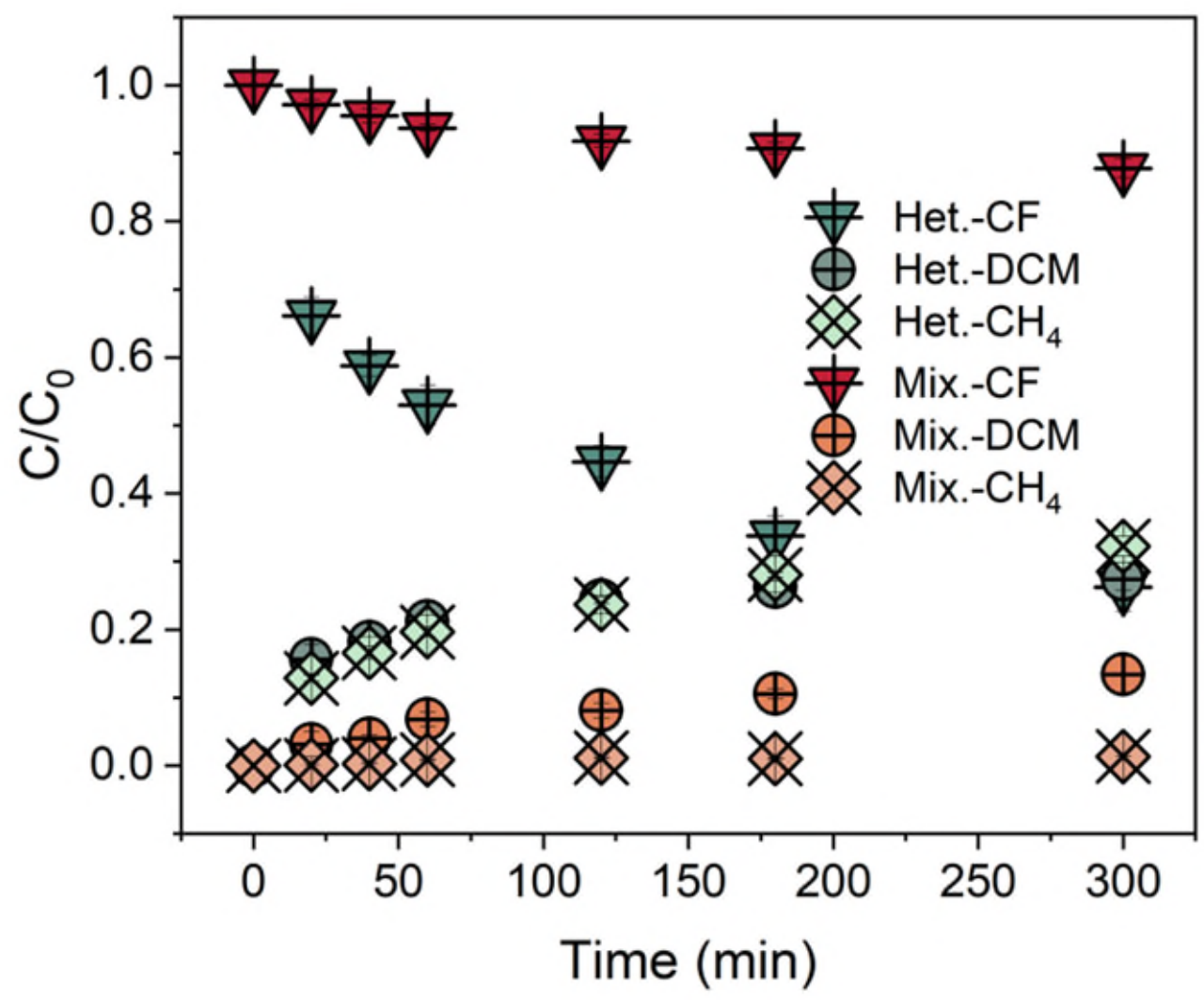

Figure S50. Comparison between $MoS_2/Fe(OH)_2$ heterojunction and physical-mixture control.

**[revision in page 11, line 296-301]**
Control experiments showed that pristine $Fe(OH)_2$ alone exhibited negligible CF degradation activity (Fig. S47), while a physically mixed $Fe(OH)_2/MoS_2$ sample, prepared with identical composition and dosage as the heterojunction, displayed substantially suppressed CF conversion and almost no CH_4 formation (Fig. S50). These results establish that simple component addition is insufficient and that tight interfacial coupling is essential for enabling selective deep hydrodechlorination.

Minor Comment: (1) References 13 and 36 are duplicates. Please verify and remove the duplicate entry or correct the numbering accordingly.

Response: Thank you for your suggestion. We have removed reference 36.

Minor Comment: (2) Reference 48 contains garbled/abnormal characters. Please check the original record and reformat the entry to ensure readability and traceability.

Response: Thank you for your suggestion. We have revised the reference section accordingly.

[revision in page 21, line 589-591]

50. Buxton, G. V., Greenstock, C. L., Helman, W. P. & Ross, A. B. Critical review of rate constants for reactions of hydrated electrons, hydrogen atoms and hydroxyl radicals (•OH/•O^-) in aqueous solution. *J. Phys. Chem. Ref.* Data **17**, 513–886 (1988).

Minor Comment: (3) The journal formatting for Angew. Chem. Int. Ed. and Phys. Rev. B (Condensed Matter–related section/abbreviation) is incorrect. Please standardize these journal titles using the official abbreviations or the format required by this journal.

Response: Thank you for your suggestion. We have revised the reference section accordingly.

[revision in page 18, line 481-482]

1. Zhang, X. et al. Reactive hydrogen species behaviors on Pd/TiN: in situ SERS guided regulation for chemoselective hydrogenation. *Angew. Chem. Int. Ed.* **64**, e202503279 (2025).

[revision in page 69 (Supplementary Information), line 417-419]

2. Kresse G.&Hafner J. Ab initio molecular dynamics for liquid metals. *Phys. Rev. B* 47, 558-561 (1993).
3. Blochl P. E. Projector augmented-wave method. *Phys. Rev. B* 50, 17953-17979 (1994).

Minor Comment: (4) In the SI, several figure captions/descriptions are missing a space between the figure label/index (e.g., Figure Sx or (a)/(b)) and the accompanying text. Please format these consistently to improve readability.

Response: We thank the reviewer for this careful observation. We have checked the Supplementary Information and corrected the formatting throughout to ensure a consistent space between each figure label/index (e.g., “Figure Sx”, “(a)/(b)”) and the accompanying caption text.

[revision in page 24 (Supplementary Information), line 194]

Figure S19. EIS spectra of (a) $MoS_2/Fe(OH)_2$-z and (b) $MoS_2/Fe(OH)_{2-y}$

Minor Comment: (5) In the SI, the pore size of the filter membrane is incorrectly written as 0.22 µM; it should be corrected to 0.22 µm.

Response: We thank the reviewer for pointing out this typographical error. The pore size of the filter membrane was incorrectly written as 0.22 µM in the Supporting Information. It has now been corrected to 0.22 µm in the revised SI.

[revision in page 3 (Supplementary Information), line 92]

To identify intermediate radicals, 2,3-dimethyl-2-butene (DMB) was employed as a spin trap for trichloromethyl radicals ($\cdot CCl_3$). The $MoS_2/Fe(OH)_2$ suspension was concentrated tenfold by centrifugation (12,000 rpm, 5 min), and the supernatant was replaced with anaerobic acetonitrile. DMB (200 mM) and CF (10 mM) were added to the reaction vial. After 1 h, the mixture was filtered (0.22 µm) and analyzed by GC/MS to detect the $\cdot CCl_3$ adduct ($TCCl_3$, 4,4,4-trichloro-2,3,3-trimethyl-1-butene).

To Reviewer #3

Comment: In this manuscript, the authors report a novel defect-engineered $MoS_2/Fe(OH)_2$ heterojunction catalyst for chloroform hydrodechlorination reaction. The long-standing kinetic trade-off between interfacial hydrogen stabilization and hydrogen mobility can be rationally overcome via defect-mediated electron-hydrogen coupling. Electronic structure analysis reveals that interfacial electric-field coupling drives strong hybridization between vacancy-derived states and the MoS_2 conduction band. This forms quasi-continuous defect-assisted tunneling channels that allow electrons to bypass the elevated Schottky barrier. This barrier-bypassing interfacial transport strategy of balancing reactive-hydrogen stabilization with mobility is interesting and innovative. Accordingly, in this context, the present study is suited for publication in Nature communication. As a whole, I would recommend this work for publication after the following issues are addressed.

Response: We sincerely thank you for the positive feedback and insightful summary of our work. We will carefully address the issues in the revised manuscript.

Comment: (1) The "reactive hydrogen" should be replaced by "active hydrogen" to more precise expression.

Response: We sincerely thank the reviewer for the valuable comment. In response to the suggestion, we have replaced "reactive hydrogen" with "active hydrogen" throughout the manuscript to ensure more precise terminology.

[revision in page 2, line 25]

Hydrogen-mediated transformations at heterointerfaces are often limited by a fundamental kinetic mismatch: the need for strong interfacial polarization to stabilize active hydrogen (H*) often creates high Schottky barriers that impede the necessary electron supply.

[revision in page 3, line 42]

Hydrogen-mediated catalytic reactions, including hydrogenation and hydrodehalogenation, critically depend on the formation, stabilization, and delivery of active hydrogen (H*).

[revision in page 3, line 48]

By extending the availability of H* beyond the original generation domains, spillover can deliver active hydrogen to locations with more favorable substrate adsorption and thereby enable selectivity that is difficult to achieve on a single-phase surface.

[revision in page 3, line 66]

This hydrogen trapping scenario hampers H* desorption, surface diffusion, and interfacial spillover, such that an apparently higher H* population does not translate into a higher flux of turnover-ready active hydrogen.

[revision in page 3, line 68]

These limitations motivate a central question in hydrogen-involving catalysis: how can one engineer a heterointerface to retain a high population of active hydrogen while maintaining a high turnover flux.

[revision in page 4, line 76]

We show that increasing the S-vacancies concentration in MoS_2 upshifts its conduction-band minimum and nominally increases the interfacial Schottky barrier, yet paradoxically enhances interfacial electron delivery and the effective flux of active hydrogen across the heterointerface.

[revision in page 10, line 266]

This trend (Fig. 3b) suggests that vacancy-rich interfaces favor retention of active hydrogen as surface/lattice species rather than as molecular H_2, consistent with the formation of an interfacial H reservoir.

[revision in page 15, line 431]

More broadly, this strategy of balancing active hydrogen stabilization with mobility establishes a general design principle for steering hydrogen-mediated pathways in complex heterogeneous catalytic systems.

[revision in page 16, line 457]

Active hydrogen species and radical intermediates were identified through electron paramagnetic resonance and radical trapping experiments, enabling distinction between single-electron transfer and hydrogen atom transfer pathways. Additional methodological details are provided in the Supporting Information.

Comment: (2) The discussion in the manuscript regarding the high selectivity of the chloroform hydrodechlorination reaction and its correlation with various

characterization results, especially the mechanism of the single-electron transfer (SET) transformation mediated by H*, is not sufficiently in-depth.

Response: We have substantially revised the sections “Vacancy-Induced Shift from SET to HAT” and “Interfacial Geometry and S-Vacancies Govern the Fate of H*” to provide a more comprehensive discussion of chloroform selectivity from two complementary perspectives: the heterojunction geometry and the role of MoS_2 S-vacancies.

In Interfacial Geometry and S-Vacancies Govern the Fate of H*, we focus on analyzing how variations in interfacial geometry and S-vacancy density influence selectivity. In Vacancy-Induced Shift from SET to HAT, we specifically examine and demonstrate the decisive role of S-vacancy density in determining the relative contribution of SET and HAT pathways.

The revisions in the Vacancy-Induced Shift from SET to HAT section significantly improve the logical flow, paragraph coherence, and overall conciseness, emphasizing that S-vacancies primarily govern pathway selectivity by controlling the fate of interfacial H*.

[revision in page 11-15, line 295-415]

Interfacial Geometry and S-Vacancies Govern the Fate of H*

Control experiments showed that pristine $Fe(OH)_2$ alone exhibited negligible CF degradation activity (Fig. S47), while a physically mixed $Fe(OH)_2/MoS_2$ sample, prepared with identical composition and dosage as the heterojunction, displayed substantially suppressed CF conversion and almost no CH_4 formation (Fig. S50). These results establish that simple component addition is insufficient and that tight interfacial coupling is essential for enabling selective deep hydrodechlorination.

Guided by this insight, we systematically investigated how the composition and structural characteristics of the $MoS_2/Fe(OH)_2$ interface govern H* pathways and electron selectivity. In particular, we focus on two independent yet synergistic factors: the interfacial contact geometry, which dictates the spatial accessibility and connectivity of H* transfer pathways, and the S-vacancy density of MoS_2, which

determines the interface's capacity to capture, stabilize, and mobilize transferable H* via continuous defect-derived electronic states. Together, these parameters define the productive interfacial hydrogen flux and thereby control both electron distribution and the selectivity toward deep CF hydrodechlorination products.

Increasing the loading of either component enhanced interfacial interactions and accelerated electron transfer (Fig. 3e), leading to an overall increase in CF degradation rate. In contrast, the product distribution exhibited pronounced sensitivity to the component ratio. At low $MoS_2/Fe(OH)_2$ ratios, TEM images revealed that $Fe(OH)_2$ extensively covered the MoS_2 surfaces and exhibited severe aggregation (Fig. S15), reflecting degraded interfacial contact geometry. Under these conditions, H* remained largely confined within aggregated $Fe(OH)_2$ regions, where short H*-H* distances favored recombination to H_2 rather than transfer to MoS_2, leading to a shift in electron selectivity toward hydrogen evolution. When $Fe(OH)_2$ loading was further increased, H_2 electron selectivity remained above 90%, indicating that hydrogen evolution dominates and reduced interfacial coupling limits deep hydrodechlorination, even in the presence of abundant H*. In this scenario, due to the lack of effectively stabilized H*, deep hydrodechlorination was limited, and the products largely remained as the initial electron-transfer intermediates, dichloromethane (DCM), resulting in low methane (CH_4) yield (Figs. 3f and 3g).

In contrast, at higher $MoS_2/Fe(OH)_2$ ratios, TEM images showed intimate contact between $Fe(OH)_2$ and MoS_2 surfaces, while MoS_2 retained ample exposed surface area enriched with coordinatively unsaturated sites (Fig. S14). Correspondingly, post-reaction Fe^{3+} concentrations increased with MoS_2 loading, suggesting that heterojunction formation enhances H* generation by $Fe(OH)_2$, likely due to an elevated Fe^{2+} reduction potential toward protons. Under this configuration, H* could be effectively captured and stabilized by vacancy-rich MoS_2. The increased population of stabilized H* facilitated interfacial hydrogen transfer, promoting deep hydrodechlorination and significantly boosting CH_4 electron selectivity while maintaining low DCM selectivity (Figs. 3f and 3g).

Having established the geometric requirements for efficient H* transfer, we next isolated the role of S-vacancy density at fixed $Fe(OH)_2$ loading (1.5 mM). When varying MoS_2 defect density, post-reaction Fe^{3+} concentrations remained nearly unchanged, indicating that the intrinsic H* generation capacity of $Fe(OH)_2$ is essentially constant. Interestingly, as shown in Fig. S51, at low S-vacancy concentrations (0%), H_2 production increased noticeably with MoS_2 loading, whereas at high S-vacancy densities (6-9%), H_2 evolution was largely insensitive to MoS_2 content. This suggests that the excess H* is effectively stabilized by MoS_2 S-vacancies. We attribute this behavior to two main factors: (i) at high MoS_2 loading, $Fe(OH)_2$ aggregation is greatly reduced, mitigating confinement of H* within aggregated domains where short H*-H* distances favor recombination; (ii) Increasing the S-vacancy density alleviates electron accumulation at the interface, facilitates the subsequent migration of H*, and provides stabilized H* sites.

All experimental groups with the same sulfur-vacancy concentrations were plotted as a unified box plot, and the results (Fig. 3h) showed that increasing sulfur-vacancy density generally accelerates chloroform hydrodechlorination kinetics. The response plot of k_1 with sulfur-vacancy concentration under the same conditions (Tables S3-S7) further supported an overall monotonic dependence within the investigated vacancy range (0-9%), with a saturation-like tendency at higher vacancy densities in a small subset of experimental groups (Fig. S46). Electronic selectivity results showed that, when the MoS_2 concentration is 0.8 g/L, as the sulfur-vacancy concentration increases, H_2 electron selectivity suppressed from 67.9% to 26.6%, while CH_4 selectivity increased from 16.0% to 56.3% and DCM selectivity showed only minor fluctuations, increasing slightly from 16.1% to 22.2% before decreasing to 17.1%. (Fig. 4a). These findings indicate that the primary function of S-vacancies is not merely to accelerate the overall reaction rate, but to inhibit electron diversion to hydrogen evolution, thereby redirecting reducing H* toward deep hydrodechlorination. This trend is further supported by adsorption-energy calculations (Fig. 4b). H* adsorption on OH sites of $Fe(OH)_2$ is energetically unfavorable (+0.58 eV), whereas H* binds strongly at S-vacancy sites on MoS_2 (−0.41

eV), providing a thermodynamic driving force for directional H* migration from $Fe(OH)_2$ toward MoS_2. The high affinity of H* for vacancy sites facilitates its stabilization and transfer at the interface, ensuring that reactive hydrogen is efficiently utilized for deep hydrodechlorination and contributing to the observed high CH_4 electron selectivity.

Collectively, these observations demonstrate that the interplay between interfacial contact geometry and S-vacancy density governs the fate of interfacial H*, establishing the necessary conditions for selective H* transfer and deep CF dechlorination.

Vacancy-Induced Shift from SET to HAT

Upon formation of the $MoS_2/Fe(OH)_2$ heterojunction, interfacial charge transfer, as well as the reduction of protons by Fe^{2+} during the reaction, can partially oxidize Fe^{2+} and may enable intervalence charge transfer between Fe^{2+} and Fe^{3+}, thereby enhancing the overall reducing capability of the $Fe(OH)_2$ phase. Under these conditions, CF reduction can proceed via two mechanistically plausible routes: a Fe-associated single-electron-transfer (SET) pathway, which generates chlorinated radical intermediates ($\cdot CCl_3$, $\cdot CHCl_2$) that may diffuse into the bulk solution, and an interfacial hydrogen-atom-transfer (HAT) pathway, in which stabilized H* exhibits continuous hydrogenation activity, enabling deep hydrodechlorination of CF.

We further demonstrated through mechanistic experiments that S-vacancies play a decisive role in determining the dominant CF reduction pathway. Mechanistic experiments were conducted at a well-dispersed $Fe(OH)_2$ concentration of 1.5 mM and a MoS_2 concentration of 0.8 g/L, ensuring strong heterojunction coupling and accessible S-vacancy sites.

Radical-trapping experiments provided the first indication of a vacancy-dependent shift. Following initial electron transfer, CF forms chlorinated radicals such as $\cdot CCl_3$ and $\cdot CHCl_2$. If these intermediates are not rapidly intercepted at the interface, they may escape into the bulk solution, where they can be captured by 2,3-dimethyl-2-butene (DMB).[46] At high S-vacancy concentrations, the system

contains a larger population of stabilized H*, resulting in lower concentrations of partially dechlorinated intermediates. Under these conditions, the HAT pathway dominates over SET, promoting sequential hydrogenation and deep CF dechlorination (Fig. S21).

Methanol-quenching experiments further probed the functional role of H*.[47] At low S-vacancy concentration (0.00%), the addition of 1 M methanol only slightly affected CF degradation and product distribution: CF degradation decreased from 46.4% to 43.6%, CH_4 yield from 9.8% to 4.9% and selectivity from 21.1% to 11.2% (Figs. 5a and 5b). By contrast, at high S-vacancy concentration (9.05%), methanol addition caused a much stronger suppression, decreasing CF degradation from 67.4% to 48.2%, CH_4 yield from 28.0% to 9.7% and selectivity from 41.5% to 20.0%. These results indicate that CF reduction in the high-vacancy system relies strongly on interfacial H*, particularly for the deep hydrodechlorination steps leading to CH_4 formation. In contrast, under low-vacancy conditions, the limited availability of transferable H* leaves the reaction closer to an SET-dominated regime.

Electrolyte modulation and kinetic-isotope-effect (KIE) measurements further supported this mechanistic transition. $NaClO_4$ perturbs the interfacial double-layer and hydrogen-bonding environment,[48, 49] affecting interfacial charge transfer and solvent reorganization at the hydroxyl-terminated $Fe(OH)_2$ surface. As a layered hydroxide, $Fe(OH)_2$ relies on its surface hydrogen-bond network to regulate local reorganization energy and gate interfacial electron transfer.[50, 51] Consequently, $Fe(OH)_2$-dominated SET processes are expected to be particularly sensitive to $NaClO_4$ addition. At low S-vacancy concentration, $NaClO_4$ decreased the apparent rate constant (k_1) from 0.00165 to 0.00120 min^{-1}, while the KIE increased only slightly from 1.11 to 1.15 (Fig. 5c). This weak isotope sensitivity indicates that hydrogen transfer contributes little to the rate-limiting step, consistent with a regime dominated by SET. In contrast, at high S-vacancy concentration, $NaClO_4$ also lowered the k_1 (0.00298 to 0.00234 min^{-1}), but the KIE increased markedly from 2.04 to 2.38 and was accompanied by enhanced CH_4 selectivity. These observations suggest that electrolyte-induced perturbations preferentially weaken $Fe(OH)_2$-associated electron

transfer, whereas the H*-mediated pathway remains comparatively robust. As a result, although the overall rate decreased, the relative contribution of HAT increased, promoting deeper hydrodechlorination.

Taken together, radical trapping, methanol quenching and KIE analyses consistently reveal a vacancy-induced rebalancing of the reaction network. Increasing S-vacancy density does not merely accelerate CF degradation; rather, it promotes the capture, stabilization and utilization of interfacial H*, suppresses radical escape and hydrogen evolution, and progressively shifts the dominant reaction regime from Fe(II)-associated SET toward interfacial H*-mediated HAT (Fig. 5e). This mechanistic transition provides the basis for the unusually high CH_4 selectivity of the $MoS_2/Fe(OH)_2$ heterojunction and underscores defect-regulated hydrogen management as a general design principle for selective reductive transformations in aqueous environments.
